# Supplementary material for: The genome of the giant Nomura’s jellyfish sheds light on the early evolution of active predation
Source: BMC Biol. 2019 Mar 29;17:28. doi: 10.1186/s12915-019-0643-7 (PMC6441219; doi:10.1186/s12915-019-0643-7)
Supplement: Supplementary file 1 — Additional Methods, Figures S1–S25, Tables S1–S20, S23, S24, S29, S38-S53. Figure S1. Species identification. Figure S2, Table S1. Estimating genome size and complexity. Figures S3–S5, Tables S2–S9. Sequencing and assembly. Figure S6. GC content distributions. Tables S10–S13. Gene and repeat annotations. Figure S7. Phylogenetic tree with divergence times. Tables S14–S20. Functional enrichment of gene family expansion and contraction. Figure S8. Domain expansion. Figure S9, Table S23. Fast-evolving gene. Figure S10. Gene age estimation. Figures S11 and S12, Table S24. Analyses of Otx and POU genes. Figure S13. Genomic context. Table S29. Transcriptome sequencing. Figures S14–S17, Tables S38–S40. Myosin type II gene analyses. Table S41. Homeobox domain. Figures S18–S21. Hox gene analyses. Figures S22 and S23, Table S42. Wnt gene analyses. Tables S43–S45. Transcriptome assembly. Tables S46–S49. Functional enrichment of development stages. Figures S24 and S25, Tables S50–S52. RXR and RAREs analyses. Table S53. Venom domain analysis. (DOCX 4760 kb) [file 12915_2019_643_MOESM1_ESM.docx]

Additional File 1

Hak-Min Kim, Jessica A. Weber, Nayoung Lee, Seung Gu Park, Yun Sung Cho, Youngjune Bhak, Nayun Lee, Yeonsu Jeon, Sungwon Jeon, Victor Luria, Amir Karger, Marc W. Kirschner, Ye Jin Jo, Seonock Woo, Kyoungsoon Shin, Oksung Chung, Jae-Chun Ryu, Hyung-Soon Yim, Jung-Hyun Lee, Jeremy S. Edwards, Andrea Manica, Jong Bhak, and Seungshic Yum

**Contents**

**1 Sample preparation**

- 1. **DNA and RNA sample preparation**
  2. **Species identification**

**2 Sequencing and assembly**

**2.1 Estimating genome size and complexity**

**2.2 Sequencing and assembly**

**3 Genome annotation**

**3.1 GC content**

**3.2 Gene annotation**

**3.3 Repeat annotation**

**4 Evolutionary analysis**

**4.1 Orthologous gene clustering and phylogenetic analysis**

**4.2 Gene family expansions and contractions.**

**4.3 Protein domain expansions**

**4.4 Fast-evolving genes**

**4.5 Gene age estimation**

**5 Genomic and transcriptomic signatures of jellyfish mobility**

**5.1 GC content at third-codon positions**

**5.2 Gene expression profiling of the jellyfish medusa bell and tentacles**

**5.3 Expansion of myosin heavy chain and light chain genes**

**6 Evolution of homeobox domain and *Wnt* gene subfamilies in cnidarians**

**6.1. Homeodomain evolution**

**6.2 ParaHox, Hox, and Hox related genes in cnidarians**

**6.3 *Wnt* gene subfamilies in cnidarians**

**7 Jellyfish developmental stage and retinoic acid signaling**

**7.1 RNA sequencing and assembly**

**7.2 Gene expression profiling and differentially expressed genes in developmental stages**

**7.3 Retinoic acid signaling in cnidarians**

**7.4 Retinoic acid response elements and nearby genes**

**8 Venom proteins in cnidarians**

**9 References**

**1 Sample preparation**

**1.1 DNA and RNA sample preparation**

The medusa from one *Nemopilema nomurai* individual was collected at Tongyeong Marine Science Station, KIOST (34.7699 N, 128.3828 E) on September 12, 2013. The surface water temperature was 24 °C. After transport to the laboratory, the medusa bell and tentacles were dissected; tissues were snap frozen in liquid nitrogen and stored at -75 °C. The polyps of *Sanderia malayensis* were provided by Aqua Planet Jeju Hanwha (Seogwipo, Korea). The polyps were fed daily with freshly hatched *Artemia* nauplii in the animal culture room, which was maintained at 24±1 °C. The metamorphosed ephyrae in the summer season were fed with *Aurelia* sp.1 medusae.

For DNA extraction, *Nemopilema* tissues were mortar-pulverized in liquid nitrogen and the powder was homogenized in a cell lysis solution [2% CTAB, 1.4 M NaCl, 100 mM Tris-Cl (pH 8.0), 20 mM EDTA, 1% β-mercaptoethanol], and incubated at 65°C for 1 hour (h). The same volume of a phenol:chloroform:isoamylalcohol (23:24:1) mixture was added to denature the proteins and the phases were separated by centrifugation at 12,000 rotations per minute (rpm) for 15 minutes (min) at room temperature. The aqueous phase was saved and incubated at 37°C for 1 h after RNase A (30 mg/ml) was added. The DNA was extracted with a phenol:chloroform:isoamyl alcohol (25:24:1) mixture, a chloroform:isoamyl alcohol (24:1) mixture was added, and the samples were centrifuged at 12,000 rpm for 15 min at room temperature. A 1/10 volume of 3 M sodium acetate (pH 5.2) and the same volume of 100% ethanol were added into the retained aqueous phase. The precipitated DNA was washed using 70% ethanol and re-suspended in an appropriate volume of ion-exchanged ultrapure water. The DNA quantity was verified by the PicoGreen method using Victor 3 fluorometry, and agarose gel electrophoresis.

RNA was extracted from jellyfish tissues, including the bell and tentacles of *Nemopilema* medusa; polyp, ephyra and medusa (bell, tentacles, and oral arms) stages of *Sanderia.* Each tissue was mortar-pulverized in liquid nitrogen. The tissue powder was then homogenized in 700 µl of lysis solution [35 mM EDTA, 0.7 M LiCl, 7% SDS, 200 mM Tris-Cl (pH 9.0)], and RNA was extracted with 700 µl of water-saturated phenol. A one-third volume of 8 M LiCl was added to the retained aqueous phase, which was maintained at 4°C for 2 h. RNA was precipitated after centrifugation at 14,000 rpm for 30 min followed by resuspension in 300 µl of DEPC-treated water. RNA was then re-precipitated with 1/10 volumes of 3 M sodium acetate (pH 5.2) and isopropanol. The precipitated RNA was rinsed with 70% ethanol (diluted in DEPC-treated water) and dissolved in an appropriate volume of DEPC-treated water (30–40 µl). The RNA quantity and integrity were analyzed using a NanoDrop ND-1000 spectrometer and an Agilent 2100 Bioanalyzer with RNA Integrity Number (RIN) value greater than 7.**1.2 Species identification**

Species identification of *Nemopilema* was confirmed by comparing the *MT-COI* gene of five species of jellyfish. We aligned *Nemopilema* Illumina short reads (~400 base pairs (bp) insert-size) to the *MT-COI* gene of *Chrysaora quinquecirrha* (NC_020459.1), *Cassiopea frondosa* (NC_016466.1), *Craspedacusta sowerbyi* (NC_018537.1), and *Aurelia aurita* (NC_008446.1) jellyfish with BWA-MEM aligner [40]. Consensus sequences for each jellyfish were generated using SAMtools [41]. The consensus sequence from *C. sowerbyi* was excluded due to low coverage. We conducted multiple sequence alignment using MUSCLE [42] and ran the MEGA v7 [43] neighbor joining phylogenetic tree (gamma distribution) with 1,000 bootstrap replicates. Three *Nemopilema* consensus sequences form a monophyletic clade within the *MT-COI* gene of *Nemopilema* (AB243416.1), which was downloaded from the NCBI database (Figure S1).


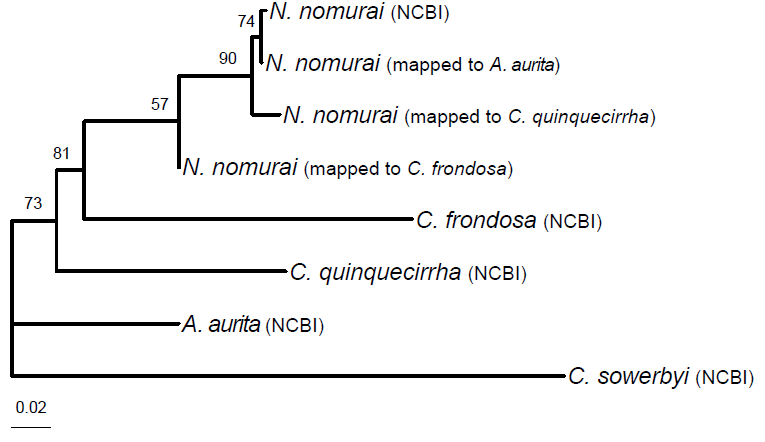


**Figure S1:** **Species identification for *Nemopilema nomurai* jellyfish.** Numbers on nodes denote bootstrap values based on 1,000 replicates. The scale bar indicates the branch lengths measured in the number of nucleotide substitutions per site.

**2 Sequencing and assembly**

**2.1 Estimating genome size and complexity**

Marine species, especially invertebrates, typically show high levels of genome heterozygosity [65], which affects the assembly quality. To determine the optimal sequencing strategies for the jellyfish assembly, we sequenced 18.5 Gb (86.8× coverage) of Illumina short (~400 bp insert size) paired-end reads to estimate the genomic complexity of *Nemopilema* using the *K*-mer analysis of the JELLYFISH program [66]. The distributions of four different *K-*mer frequency plots showed two peaks in the *Nemopilema* genome (Figure S2), indicating that it has a highly heterozygous genome. From a *K*-mer (*K*=17, 19, 21, and 23) analysis, the *Nemopilema* genome size was estimated to be 211~221 Mb (Table S1).

**
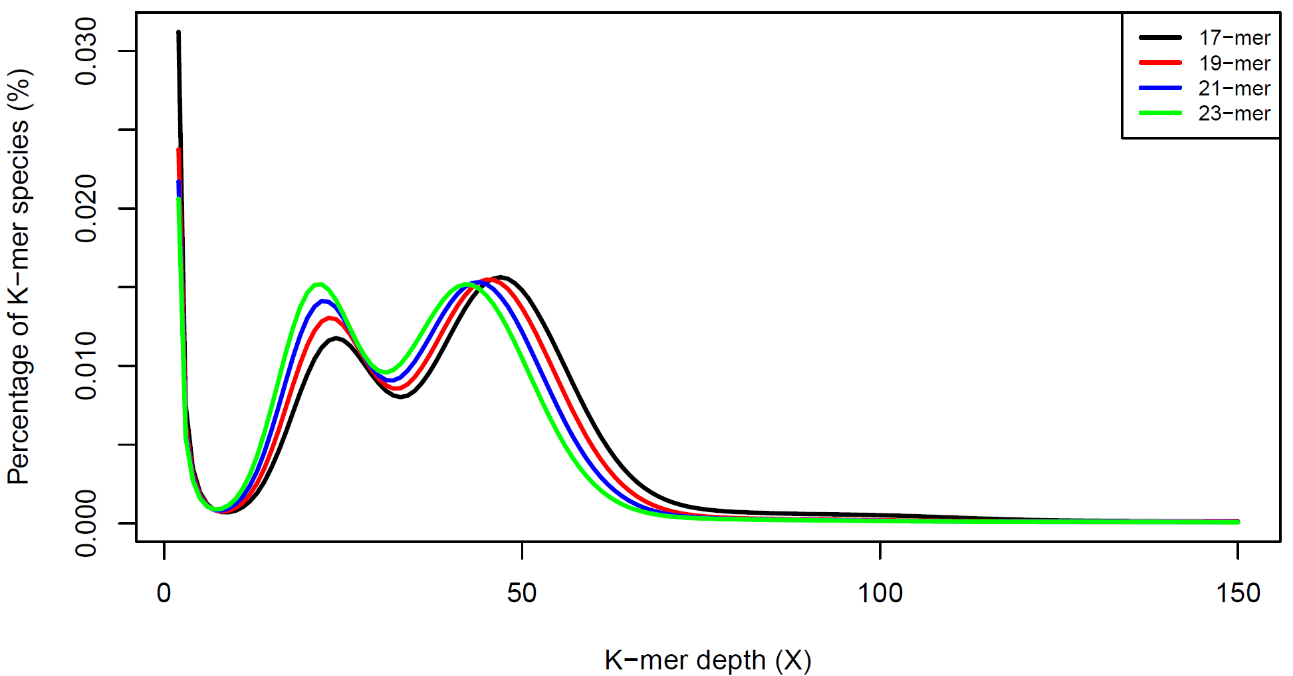
**

**Figure S2:** **Distribution of K-mer frequencies for 17-, 19-, 21-, and 23-mers using the error-corrected reads.**

**Table S1: *K*-mer analysis for estimating genome size.**

| ***K*-mer size** | **Total *K*-mer count** | **Peak depth** | **Estimated genome size** |
| --- | --- | --- | --- |
| 17 | 9,934,145,023 | 47 | 211,364,788 |
| 19 | 9,732,013,270 | 45 | 216,266,962 |
| 21 | 9,520,986,025 | 44 | 216,386,046 |
| 23 | 9,302,429,653 | 42 | 221,486,420 |

**2.2 Sequencing and assembly**

To minimize heterozygosity bias, we sequenced and assembled the jellyfish genome using the following sequencing data: Pacific Biosciences (PacBio) single molecule real time sequencing (SMRT) reads, Illumina TruSeq synthetic long reads (TSLRs), and Illumina mate-pair reads. First, the extracted genomic DNA was sequenced to a 179× average sequencing depth of coverage using a Pacific Biosciences RSII instrument with SMRT cell 8Pac V3 and DNA Polymerase Binding Kit P6 reagents (30 SMRT cells), as a major sequencing data source for a contig assembly. We obtained 11.4 Kb of median (N50) length of quality filtered PacBio subreads (Figure S3 and Table S4). We assembled multiple contig sets using the Falcon assembler [44] with the quality filtered PacBio SMRT subreads from a diverse set of read length cutoffs (5 Kb, 6 Kb, 7 Kb, 8 Kb, 9 Kb, 10 Kb, and 12 Kb; Figure S5 and Table S2). Second, to extend the contigs into scaffolds, we additionally generated a set of Illumina long mate-pair libraries (5 Kb, 10 Kb, 15 Kb, and 20 Kb; Table S5). PCR duplicated, sequencing and junction adaptor contaminated, and low quality (<Q20) reads were filtered out, leaving only highly accurate reads for genome assembly (Table S6). Additionally, short insert size and long insert size reads were trimmed into 90 bp and 50 bp, respectively, to remove low quality end sequences. We concatenated the contigs to scaffolds using SSPACE [67] and the gaps were filled by aligning the Illumina short paired-end sequences using GapCloser [46]. The scaffold set that was closest to the predicted genome size with the longest N50 length was selected and used for further analyses (Table S3). A total of 255 scaffolds were generated, totaling 213 Mb of sequence length with a N50 length of 2.71 Mb containing only 1.48 % of gaps. Just 92 scaffolds (N90 of 524Kb) successfully covered 90% of the jellyfish genome. However, genome assemblies constructed using PacBio SMRT reads often contain erroneous sequences (~15%), which are derived from low-quality SMRT reads [68]. Conversely, Illumina TSLRs are generated by local assembly of the high-quality short reads [69]. Therefore, we generated 1.92 Gb (~9× coverage) of Illumina TSLRs (Figure S4 and Table S7) to correct erroneous sequences in the PacBio long-read assembly and to close gap regions. To correct base-pair level errors, we performed three iterations of aligning the Illumina short paired-end sequence to the scaffolds using BWA-MEM [40] and calling variants using SAMtools [41]. Homozygous variants were substituted using an in-house script. The assembly quality was assessed by aligning the short sequence reads onto the final scaffolds (~99% of mapping rate; Table S8) and by comparing the assembly statistics of other metazoan species. The jellyfish assembly showed the longest assembly continuity among the cnidarian genomes (Table S9).


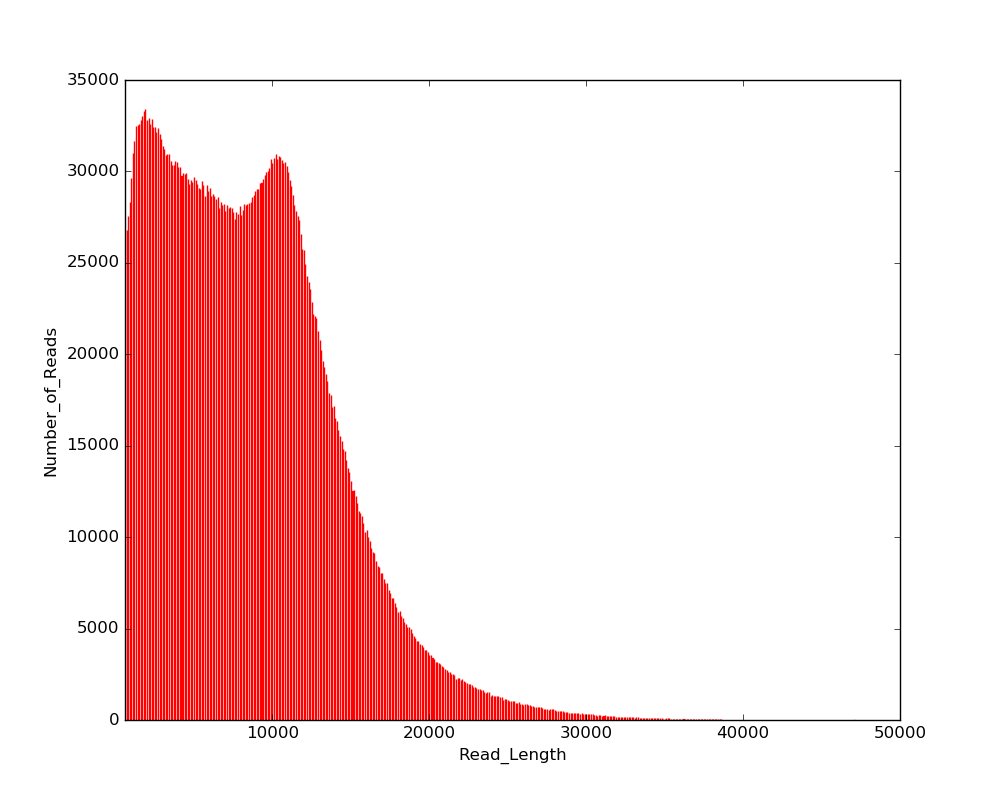


**Figure S3: Length distribution of PacBio SMRT reads.**


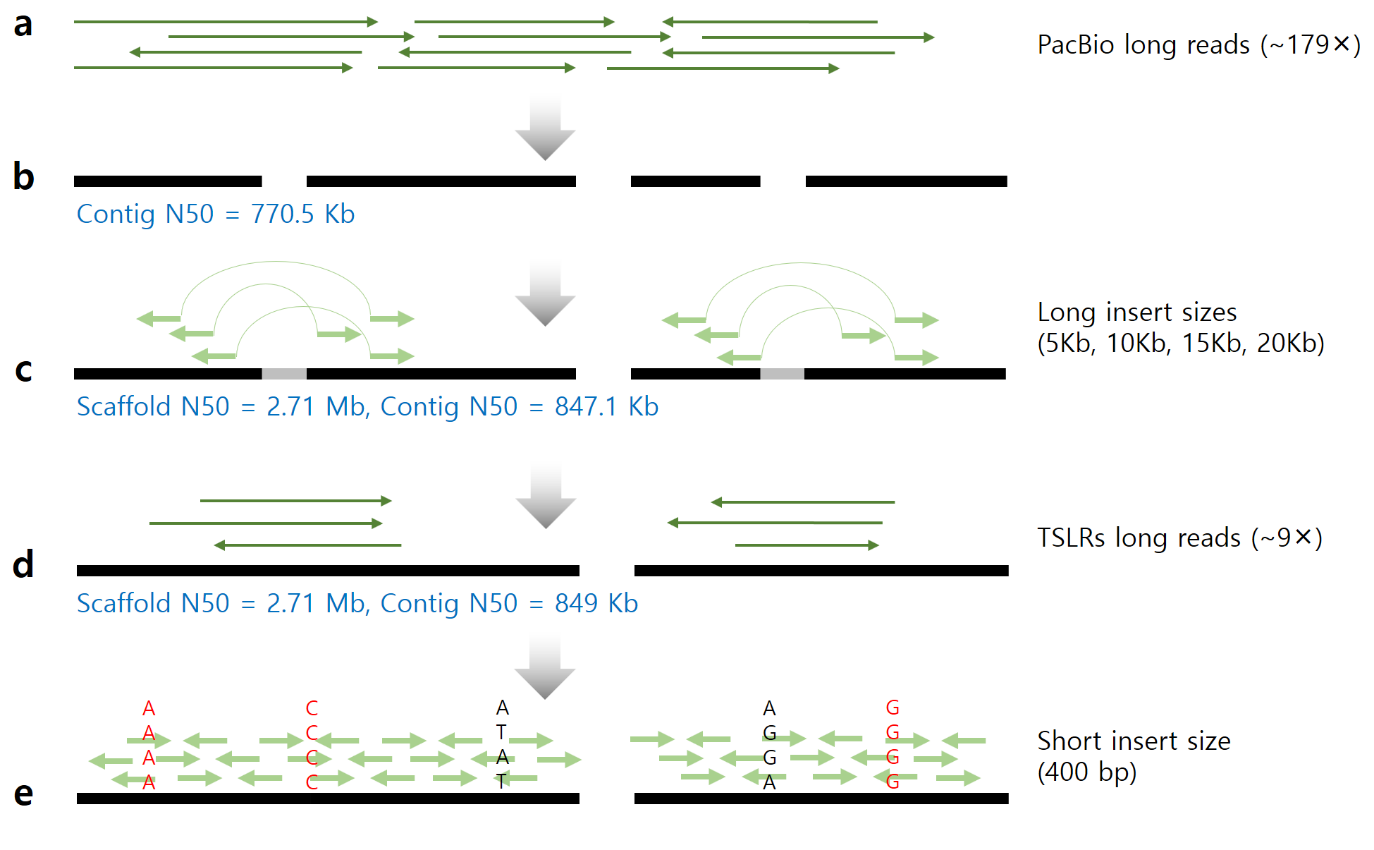


**Figure S4: Schematic overview of the *Nemopilema nomurai* genome assembly process. a,** Unassembled PacBio SMRT long reads. **b,** Contig assembly using PacBio long reads and the Falcon assembler. **c,** Scaffold assembly using the Illumina mate pair libraries. **d,** Gap closing using Illumina TruSeq synthetic long reads (TSLR). **e,** Substitution of common variants using the short insert library. Red denotes common variant that is substituted in the genome.


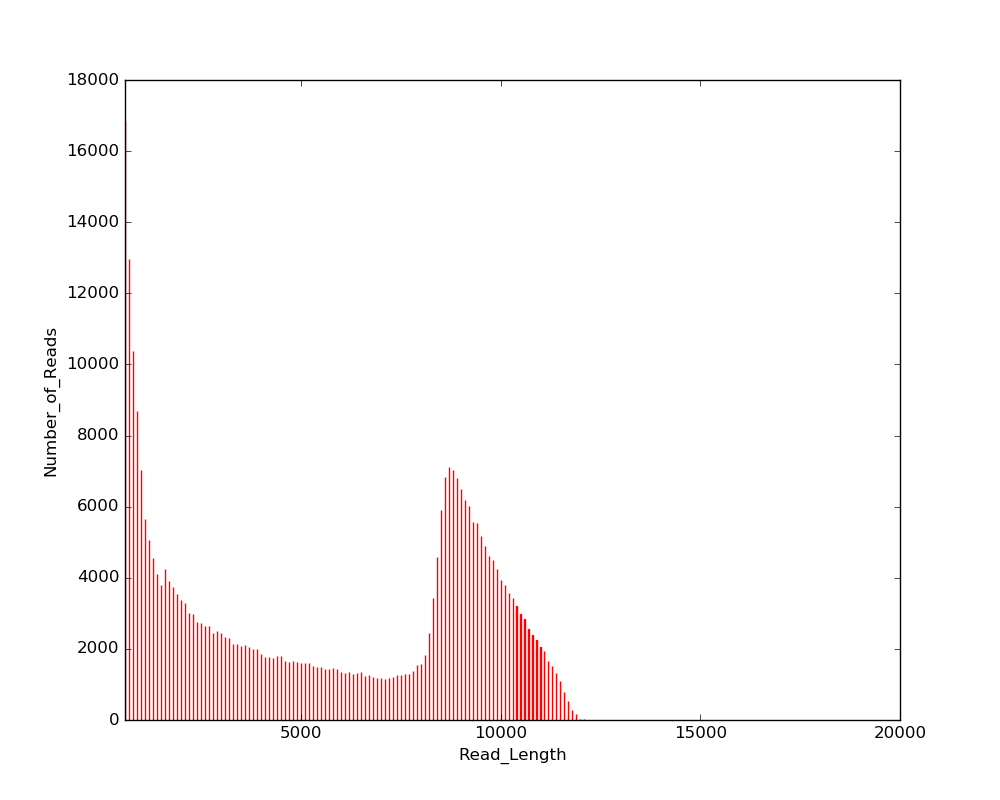


**Figure S5: Length distribution of Illumina TruSeq synthetic long reads.**

**Table S2: Contig assembly statistics using PacBio SMRT reads.**

|  | **PacBio long read length cutoffs** | | | | | | |
| --- | --- | --- | --- | --- | --- | --- | --- |
|  | **5Kb** | **6Kb** | **7Kb** | **8Kb** | **9Kb** | **10Kb** | **12Kb** |
| # of sequences | 2,519 | 2,453 | 2,078 | 1,570 | 1,456 | 1,140 | 1,237 |
| Total bases | 221,141,034 | 221,771,871 | 217,392,668 | 211,465,427 | 209,338,243 | 203,154,934 | 195,823,825 |
| Longest sequence | 3,777,904 | 3,622,163 | 3,634,349 | 5,223,426 | 4,088,286 | 4,357,459 | 2,392,030 |
| Shortest sequence | 2 | 9 | 2 | 10 | 14 | 10 | 26 |
| N50 | 609,640 | 570,382 | 669,977 | 794,113 | 770,490 | 952,382 | 490,833 |
| GC % | 38.02% | 37.99% | 38.07% | 38.17% | 38.21% | 38.20% | 38.25% |
| N bases | 0.00% | 0.00% | 0.00% | 0.00% | 0.00% | 0.00% | 0.00% |

**Table S3: Scaffold assembly statistics using PacBio SMRT reads and Illumina mate-pair reads.**

|  | **PacBio long read length cutoffs** | | | | | | |
| --- | --- | --- | --- | --- | --- | --- | --- |
|  | **5Kb** | **6Kb** | **7Kb** | **8Kb** | **9Kb** | **10Kb** | **12Kb** |
| Number of sequences | 527 | 464 | 465 | 287 | 255 | 185 | 321 |
| Total bases | 228,171,285 | 228,617,968 | 222,893,641 | 215,793,878 | 213,630,333 | 206,423,756 | 199,029,964 |
| Longest sequence | 7,076,075 | 5,650,389 | 6,910,851 | 6,464,488 | 8,551,441 | 11,878,115 | 3,985,671 |
| Shortest sequence | 2 | 9 | 2 | 10 | 14 | 10 | 26 |
| N50 | 2,266,714 | 2,149,743 | 1,759,166 | 2,209,994 | 2,711,397 | 3,064,082 | 1,204,326 |
| GC % | 38.04% | 38.00% | 38.08% | 38.18% | 38.23% | 38.22% | 38.26% |
| N bases | 2.53% | 2.45% | 1.98% | 1.53% | 1.48% | 1.14% | 1.14% |

**Table S4: PacBio SMRT sequence statistics.**

| Number of sequences | 4,592,385 ea |
| --- | --- |
| Total bases | 38,170,953,026 bp |
| Average length | 8,311.79 bp |
| Longest length | 50,973 bp |
| Shortest length | 35 bp |
| N50 | 11,383 bp |
| GC contents | 38.60% |
| N bases | 0.00% |

**Table S5: Illumina sequence statistics.**

| **Insert-size** | **Library** | **Total number of reads** | **Read length (bp)** | **Total bases (bp)** | **Depth  (×, divided by 213Mb)** | **Total depth (×)** |
| --- | --- | --- | --- | --- | --- | --- |
| 400bp | L1_1 | 92,425,518 | 100 | 9,242,551,800 | 43.4 | 86.8 |
|  | L1_2 | 92,425,518 | 100 | 9,242,551,800 | 43.4 |  |
| 5Kb | L1_1 | 133,617,621 | 101 | 13,495,379,721 | 63.4 | 126.7 |
|  | L1_2 | 133,617,621 | 101 | 13,495,379,721 | 63.4 |  |
| 10Kb | L1_1 | 112,497,349 | 101 | 11,362,232,249 | 53.3 | 106.7 |
|  | L1_2 | 112,497,349 | 101 | 11,362,232,249 | 53.3 |  |
| 15Kb | L1_1 | 70,627,241 | 100 | 7,062,724,100 | 33.2 | 66.3 |
|  | L1_2 | 70,627,241 | 100 | 7,062,724,100 | 33.2 |  |
| 20Kb | L1_1 | 71,850,585 | 100 | 7,185,058,500 | 33.7 | 67.5 |
|  | L1_2 | 71,850,585 | 100 | 7,185,058,500 | 33.7 |  |
| Total | - | 962,036,628 | - | 96,695,892,740 | 454 | 454 |

**Table S6: Filtered Illumina sequence statistics.**

| **Insert-size** | **Library** | **Total number of reads** | **Read length (bp)** | **Total bases (bp)** | **Depth  (×, divided by 213Mb)** | **Total depth (×)** |
| --- | --- | --- | --- | --- | --- | --- |
| 400bp | L1_1 | 86,434,438 | 90 | 7,779,099,420 | 40.58 | 81.16 |
|  | L1_2 | 86,434,438 | 90 | 7,779,099,420 | 40.58 |  |
| 5Kb | L1_1 | 21,407,082 | 50 | 1,070,354,100 | 10.05 | 20.10 |
|  | L1_2 | 21,407,082 | 50 | 1,070,354,100 | 10.05 |  |
| 10Kb | L1_1 | 16,094,130 | 50 | 804,706,500 | 7.56 | 15.11 |
|  | L1_2 | 16,094,130 | 50 | 804,706,500 | 7.56 |  |
| 15Kb | L1_1 | 9,090,529 | 50 | 454,526,450 | 4.27 | 8.54 |
|  | L1_2 | 9,090,529 | 50 | 454,526,450 | 4.27 |  |
| 20Kb | L1_1 | 9,965,208 | 50 | 498,260,400 | 4.68 | 9.36 |
|  | L1_2 | 9,965,208 | 50 | 498,260,400 | 4.68 |  |
| Total | - | 285,982,774 | - | 21,213,893,740 | 134.3 | 134.3 |

**Table S7: Illumina TruSeq Synthetic Long Reads statistics.**

| Number of sequences | 345,790 ea |
| --- | --- |
| Total bases | 1,922,851,266 bp |
| Average length | 5,560.75 bp |
| Longest length | 20,642 bp |
| Shortest length | 500 bp |
| N50 | 8,880 bp |
| GC contents | 38.04% |
| N bases | 0.00% |

**Table S8: Assembly quality assessment by mapping Illumina reads to *Nemopilema* assembly.**

| Assembly | Mapping rate | | | | |
| --- | --- | --- | --- | --- | --- |
|  | 400bp | 5Kb | 10Kb | 15Kb | 20Kb |
| *Nemopilema* | 99.74% | 99.14% | 99.06% | 99.02% | 98.71% |

**Table S9: Assembly statistics of nine metazoans and choanoflagellate.**

| Phylum | Species | NCBI version | # of  sequences | Total  bases (bp) | Longest  length (bp) | Shortest  length (bp) | Scaffold  N50 (bp) | Contig  N50 (bp) | GC  ratio | Gap  proportion |
| --- | --- | --- | --- | --- | --- | --- | --- | --- | --- | --- |
| Cnidaria | *Nemopilema nomurai* | N/A | 255 | 213,630,333 | 8,551,441 | 288 | 2,711,397 | 849,297 | 38.23% | 1.48% |
|  | *Aurelia aurita* | N/A | 25454 | 757,170,055 | 1,038,510 | 1001 | 121,658 | 14,693 | 37.48% | 12.85% |
|  | *Hydra vulgaris* | Hydra_ RP_1.0 | 20,916 | 852,170,992 | 908,834 | 2,000 | 96,317 | 10,112 | 27.57% | 7.83% |
|  | *Clytia hemisphaerica* | N/A | 7,644 | 445,210,140 | 2,888,473 | 501 | 366,311 | 3,860 | 35.34% | 16.63% |
|  | *Nematostella vectensis* | ASM 20922v1 | 10,804 | 356,613,585 | 3,256,212 | 626 | 472,588 | 19,244 | 40.64% | 16.61% |
|  | *Acropora digitifera* | Adig_1.1 | 2,421 | 447,497,157 | 2,549,845 | 2,003 | 483,559 | 10,915 | 39.04% | 15.24% |
| Placozoa | *Trichoplax adhaerens* | v1.0 | 1,414 | 105,631,681 | 13,260,704 | 1,000 | 5,978,658 | 190,696 | 32.74% | 10.30% |
| Porifera | *Amphimedon queenslandica* | v1.0 | 13,398 | 166,699,561 | 1,888,931 | 633 | 120,365 | 11,710 | 35.83% | 13.10% |
| Ctenophora | *Mnemiopsis leidyi* | MneLei Aug-11 | 5,100 | 155,865,547 | 1,222,598 | 987 | 187,314 | 11,817 | 38.86% | 3.55% |
| Holozoa | *Monosiga brevicollis* | v1.0 | 219 | 41,709,928 | 3,607,471 | 1,005 | 1,073,601 | 48,633 | 54.81% | 7.16% |

**3 Genome annotation**

**3.1 GC content**

Genome-wide GC distributions vary between species, which can result in sequencing biases. We examined the GC content distributions among the four cnidarian genomes along with the human. We used a 500 bp sliding window along the genomes, with a 250 bp overlap between two adjacent windows. In general, species that are more closely related are expected to possess similar distribution curves. However, cnidarian species showed very different GC content distributions (Figure S6). The GC content of *Nemopilema nomurai* is a slightly lower than *Acropora* *digitifera* and *Nematostella vectensis*, but much higher than *Hydra vulgaris*.


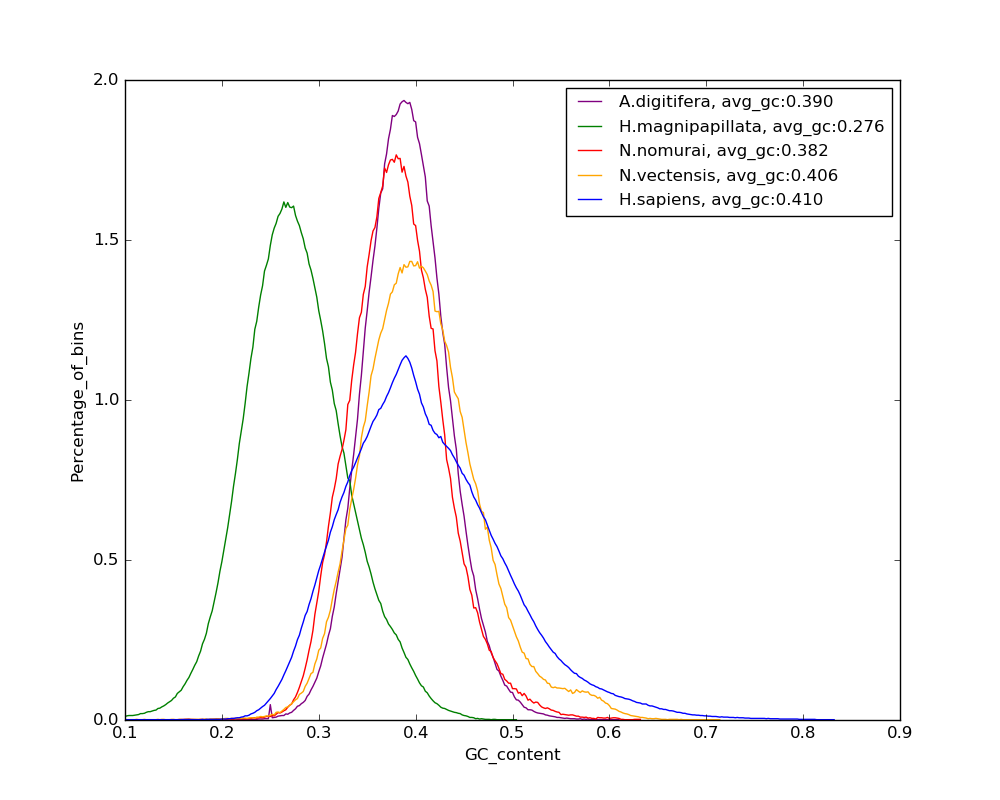
 **Figure S6: GC content distributions among cnidarian genomes.3.2 Gene annotation**

For the protein-coding gene prediction, we applied both homology-based and evidence-based gene prediction methods. For the homology gene prediction, we searched for sea anemone, hydra, sponge, human, mouse, and fruit fly protein sequences from NCBI database, and Cnidaria protein sequences from NCBI Entrez protein database using TblastN [70] with an *E*-value cutoff of 1E-5. The matched sequences were clustered using GenBlastA [71] and filtered by coverage and identity of >40% criterion. Gene models were predicted using the Exonerate program [72] and exon hints were extracted using the exonerate2hints.pl script of the AUGUSTUS program [47]. For the evidence-based gene prediction, we aligned the bell and tentacle RNA-seq reads to the repeat masked jellyfish genome assembly using the TopHat program (v2.0.9) [73]. To remove redundantly aligned reads, we filtered the alignment results with the --uniq option using the filterBam command of AUGUSTUS. Intron hints were generated using the bam2hints command of AUGUSTUS. Jellyfish protein-coding genes were determined using AUGUSTUS with the exon and intron hints with >=30 amino acids criteria (Table S10). Finally, we filtered the protein-coding genes that had breaks in the three-letter codon frame, premature stop codons, and ambiguous bases in the CDS (Table S11). The genome assembly and gene annotation completeness were assessed by the commonly used single-copy ortholog mapping approach [7] (Table S12).

**Table S10: Statistics of pre-filtered protein-coding gene properties in metazoans and holozoan.**

| Species | | # of protein-coding genes | | Avg. CDS length (bp) | | Avg. exon  count | | Avg. intron  length (bp) | Avg. third codon GC ratio (%) | |
| --- | --- | --- | --- | --- | --- | --- | --- | --- | --- | --- |
| *N. nomurai* | 19,525 | | 1,456.6 | | 7.6 | | 689.8 | | | 0.444 |
| *A. aurita* | 33,131 | | 1027.3 | | 3.7 | | 1811.7 | | | 0.391 |
| *H. vulgaris* | 20,042 | | 1,255.3 | | 6.0 | | 2,600.0 | | | 0.263 |
| *N. vectensis* | 24,773 | | 1,004.2 | | 5.3 | | 797.5 | | | 0.494 |
| *A. digitifera* | 26,060 | | 1,337.0 | | 6.2 | | 1,103.6 | | | 0.420 |
| *T. adhaerens* | 11,518 | | 1,359.4 | | 8.4 | | 283.3 | | | 0.310 |
| *A. queenslandica* | 13,624 | | 1,470.4 | | 8.0 | | 263.9 | | | 0.379 |
| *M. leidyi* | 16,548 | | 1,384.5 | | 5.5 | | 896.5 | | | 0.480 |
| *M. brevicollis* | 9,171 | | 1,801.1 | | 7.5 | | 170.1 | | | 0.650 |
| *C. elegans* | 20,257 | | 1,233.6 | | 6.1 | | 307.2 | | | 0.405 |
| *D. rerio* | 26,382 | | 1,680.1 | | 9.4 | | 2,792.5 | | | 0.547 |
| *D. melanogaster* | 13,906 | | 1,608.6 | | 4.0 | | 972.9 | | | 0.639 |
| *H. sapiens* | 19,856 | | 1,738.1 | | 9.9 | | 5,456.3 | | | 0.599 |

**Table S11: Statistics of post-filtered protein-coding gene properties in metazoans and holozoan.**

| Species | # of protein-coding genes | Avg. CDS length (bp) | Avg. exon count | Avg. intron  length (bp) | Avg. third codon  GC ratio (%) |
| --- | --- | --- | --- | --- | --- |
| *N. nomurai* | 18,962 | 1,441.3 | 7.5 | 691.0 | 0.444 |
| *A. aurita* | 25,174 | 1,173.9 | 4.2 | 1806.1 | 0.375 |
| *H. vulgaris* | 17,331 | 1,220.2 | 5.5 | 2,612.1 | 0.246 |
| *N. vectensis* | 24,567 | 1,003.0 | 5.3 | 795.6 | 0.494 |
| *A. digitifera* | 25,295 | 1,315.0 | 6.0 | 1,118.8 | 0.420 |
| *T. adhaerens* | 11,491 | 1,359.6 | 8.4 | 283.3 | 0.310 |
| *A. queenslandica* | 12,811 | 1,478.9 | 8.0 | 263.6 | 0.376 |
| *M. leidyi* | 15,922 | 1,385.0 | 5.5 | 884.8 | 0.480 |
| *M. brevicollis* | 9,153 | 1,801.0 | 7.5 | 169.3 | 0.650 |
| *C. elegans* | 20,256 | 1,233.5 | 6.1 | 307.2 | 0.405 |
| *D. rerio* | 25,654 | 1,680.8 | 9.4 | 2,796.9 | 0.547 |
| *D. melanogaster* | 13,864 | 1,603.7 | 4.0 | 973.7 | 0.639 |
| *H. sapiens* | 19,797 | 1,735.8 | 9.9 | 5,472.2 | 0.599 |

**Table S12: Gene-set quality assessment using a single-copy ortholog mapping approach.**

| Species | Complete | | | Duplicate | | | Fragment | | | Missing | | | Total BUSCO genes |
| --- | --- | --- | --- | --- | --- | --- | --- | --- | --- | --- | --- | --- | --- |
|  | Count | | % | Count | | % | Count | | % | Count | | % |  |
| *N. nomurai* | 409 | 95.30% | | 150 | 35.00% | | 12 | 2.80% | | 8 | 1.90% | | 429 |
| *A. aurita* | 323 | 75.30% | | 139 | 32.40% | | 48 | 11.20% | | 58 | 13.50% | | 429 |
| *H. vulgaris* | 401 | 93.50% | | 129 | 30.10% | | 16 | 3.70% | | 12 | 2.80% | | 429 |
| *A. digitifera* | 342 | 79.70% | | 122 | 28.40% | | 65 | 15.20% | | 22 | 5.10% | | 429 |
| *N. vectensis* | 383 | 89.30% | | 133 | 31.00% | | 29 | 6.80% | | 17 | 4.00% | | 429 |
| *T. adhaerens* | 397 | 92.50% | | 101 | 23.50% | | 22 | 5.10% | | 10 | 2.30% | | 429 |
| *A. queenslandica* | 390 | 90.90% | | 124 | 28.90% | | 24 | 5.60% | | 15 | 3.50% | | 429 |
| *M. leidyi* | 371 | 86.50% | | 88 | 20.50% | | 32 | 7.50% | | 26 | 6.10% | | 429 |
| *M. brevicollis* | 349 | 81.40% | | 86 | 20.00% | | 34 | 7.90% | | 46 | 10.70% | | 429 |
| *C. elegans* | 417 | 97.20% | | 105 | 24.50% | | 4 | 0.90% | | 8 | 1.90% | | 429 |
| *D. rerio* | 424 | 98.80% | | 156 | 36.40% | | 4 | 0.90% | | 1 | 0.20% | | 429 |
| *D. melanogaster* | 425 | 99.10% | | 133 | 31.00% | | 0 | 0.00% | | 4 | 0.90% | | 429 |
| *H. sapiens* | 426 | 99.30% | | 145 | 33.80% | | 2 | 0.50% | | 1 | 0.20% | | 429 |

**3.3 Repeat annotation**

To annotate the repetitive elements in the jellyfish genome, we searched the genome for tandem repeats using the Tandem Repeats Finder database [48]. Transposable elements (TEs) were identified using both homology-based and *ab initio*-based approaches. The Repbase [74] database version 19.03 was used for the homology-based approach to identify repeats using RepeatMasker (version 4.0.5) [49] and RMBlast (version 2.2.28). For the *ab initio*-based approach, we used RepeatModeler version 1.0.7 [75]. All predicted repetitive elements were merged prior to statistical analyses using in-house scripts. These methods were then adapted to predict repeat elements in the other published cnidarian genomes (*Nematostella*, *Acropora*, and *Hydra*) (Table S13).

**Table S13: Repeat annotation of cnidarians.**

| Repeat type | *Nemopilema  nomurai* | *Nematostella vectensis* | *Acropora digitifera* | *Hydra vulgaris* |
| --- | --- | --- | --- | --- |
| DNA | 5,440,773 | 55,668,977 | 10,897,434 | 173,628,759 |
| LINE | 2,291,406 | 7,410,687 | 9,107,195 | 122,090,336 |
| LTR | 1,740,085 | 8,222,409 | 7,747,783 | 7,453,663 |
| Low complexity | 269,113 | 380,420 | 637,357 | 6,093,798 |
| Retroposon | - | 1,508 | - | 1,959 |
| SINE | 136,032 | 10,839 | 49,143 | 15,574 |
| Satellite | 33,340 | 9,089,245 | 148,098 | 130,167 |
| Simple repeat | 2,641,456 | 4,807,011 | 4,452,246 | 37,670,762 |
| Tandem repeat | 19,010,792 | 40,720,293 | 10,842,313 | 55,194,832 |
| Unknown | 27,423,499 | 3,852,181 | 973,386 | 29,189 |
| Unspecified | - | 2,621,786 | 1,206,277 | 2,967,316 |
| Total TE | 45,007,573 | 119,934,142 | 42,310,111 | 365,319,848 |
| Genome size | 213,630,333 | 356,613,585 | 447,497,157 | 852,170,992 |
| % of repeat elements | 21.07% | 33.63% | 9.45% | 42.87% |

**4 Evolutionary analysis**

**4.1 Orthologous gene clustering and phylogenetic analysis**

Orthologous gene clustering of protein-coding genes from 13 metazoans (*Nemopilema nomurai*, *Aurelia aurita*, *Hydra vulgaris*, *Clytia hemisphaerica*, *Acropora digitifera*, *Nematostella vectensis*, *Caenorhabditis elegans*, *Danio rerio*, *Drosophila melanogaster*, *Homo sapiens*, *Trichoplax adhaerens*, *Amphimedon queenslandica*, and *Mnemiopsis leidyi*) and one unicellular holozoan (*Monosiga brevicollis*: as an out-group) was conducted using the OrthoMCL (version 2.0.9) program [53]. We found 150 single-copy gene families in the 14 species. To infer the jellyfish phylogeny, we used protein sequences of 150 single-copy gene families, and the PROTGAMMAJTT model in the RAxML (version 8.2.8) program [54]. We also estimated the divergence time using the MEGA7 program [43] with the phylogenetic topology. The divergence date of the zebrafish-human node was constrained to 435 million years ago (MYA) and the choanoflagellate -human was constrained to 1,023 MYA based on the TimeTree database [76]. As expected, *Nemopilema* and *Aurelia* formed a monophyletic clade (Figure S7).


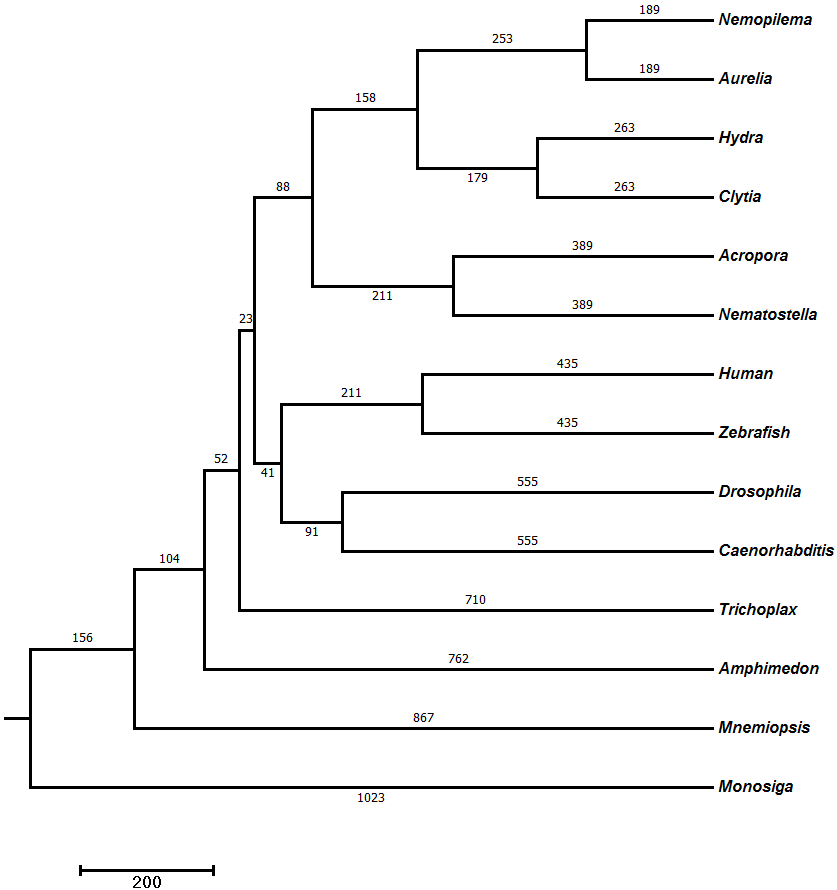


**Figure S7: Phylogenetic tree with divergence times using 150 single-copy genes.** Numbers on branches denote branch length (divergence time, MYA).

**4.2 Gene family expansions and contractions**

Changes in the size of gene families can result from adaptive evolution of species, relaxation of selection pressures, or stochastic effects [77]. To examine changes in gene families of *Nemopilema*, a gene family expansion and contraction analysis was conducted using the CAFÉ program [55] with the estimated divergence times and phylogenetic tree topology. We used a *P*-value < 0.05 criterion for significantly changed gene families. Functional enrichment tests for expanded and contracted gene families were performed using the DAVID Bioinformatics Resources program [78]. Cnidaria gene family expansions and contractions were defined by comparing the common ancestor of Cnidaria with common ancestor of Cnidaria-Bilateria (Tables S14 and S15). Scyphozoa gene family expansions and contractions were defined by comparing the common ancestor of Scyphozoa with common ancestor of Scyphozoa-Hydrozoa (Tables S16 and S17). Similarly, the expansion and contraction of gene families in *Nemopilema* were defined by comparing the *Nemopilema* branch with the *Nemopilema*-*Aurelia* common ancestor (Tables S18 and S19). Additionally, we found 1,589 orthologous gene families that are specific to scyphozoans (Table S20).

**Table S14: GO enrichment of genes that were contracted in Cnidaria compared to the common ancestor of Cnidaria and Bilateria.**

| Term | Count | *P*-value | FDR |
| --- | --- | --- | --- |
| GO:0005882~intermediate filament | 41 | 3.98E-49 | 4.59E-46 |
| GO:0050911~detection of chemical stimulus involved in sensory perception of smell | 52 | 1.50E-38 | 2.16E-35 |
| GO:0004984~olfactory receptor activity | 52 | 3.16E-37 | 3.71E-34 |
| GO:0005198~structural molecule activity | 42 | 6.96E-36 | 8.18E-33 |
| GO:0004930~G-protein coupled receptor activity | 52 | 1.05E-26 | 1.23E-23 |
| GO:0045095~keratin filament | 26 | 1.45E-26 | 1.67E-23 |
| GO:0007165~signal transduction | 60 | 2.68E-24 | 3.86E-21 |
| GO:0016021~integral component of membrane | 131 | 2.30E-23 | 2.66E-20 |
| GO:0007186~G-protein coupled receptor signaling pathway | 50 | 2.78E-21 | 4.00E-18 |
| GO:0005886~plasma membrane | 109 | 2.41E-19 | 2.77E-16 |
| GO:0009813~flavonoid biosynthetic process | 13 | 2.76E-19 | 3.97E-16 |
| GO:0052696~flavonoid glucuronidation | 13 | 1.39E-18 | 2.00E-15 |
| GO:0015020~glucuronosyltransferase activity | 13 | 1.73E-16 | 2.55E-13 |
| GO:0071556~integral component of lumenal side of endoplasmic reticulum membrane | 13 | 2.16E-16 | 2.55E-13 |
| GO:0042605~peptide antigen binding | 12 | 6.67E-15 | 7.82E-12 |
| GO:0012507~ER to Golgi transport vesicle membrane | 14 | 2.38E-14 | 2.74E-11 |
| GO:0019882~antigen processing and presentation | 14 | 2.40E-14 | 3.45E-11 |
| GO:0042612~MHC class I protein complex | 9 | 5.08E-14 | 5.86E-11 |
| GO:0042613~MHC class II protein complex | 10 | 1.56E-12 | 1.80E-09 |
| GO:0032395~MHC class II receptor activity | 9 | 1.65E-12 | 1.94E-09 |
| GO:0005234~extracellular-glutamate-gated ion channel activity | 9 | 1.09E-11 | 1.28E-08 |
| GO:0060333~interferon-gamma-mediated signaling pathway | 13 | 1.73E-11 | 2.49E-08 |
| GO:0004970~ionotropic glutamate receptor activity | 8 | 1.47E-10 | 1.72E-07 |
| GO:0035235~ionotropic glutamate receptor signaling pathway | 9 | 1.23E-10 | 1.77E-07 |
| GO:0008544~epidermis development | 13 | 1.55E-10 | 2.24E-07 |
| GO:0002504~antigen processing and presentation of peptide or polysaccharide antigen via MHC class II | 8 | 3.24E-10 | 4.66E-07 |
| GO:0002474~antigen processing and presentation of peptide antigen via MHC class I | 9 | 9.26E-10 | 1.33E-06 |
| GO:0016758~transferase activity, transferring hexosyl groups | 9 | 1.29E-09 | 1.52E-06 |
| GO:0030666~endocytic vesicle membrane | 11 | 4.80E-09 | 5.54E-06 |
| GO:0034332~adherens junction organization | 9 | 5.72E-09 | 8.23E-06 |
| GO:0052695~cellular glucuronidation | 7 | 1.24E-08 | 1.79E-05 |
| GO:0002480~antigen processing and presentation of exogenous peptide antigen via MHC class I, TAP-independent | 6 | 1.92E-08 | 2.76E-05 |
| GO:0031090~organelle membrane | 11 | 7.35E-08 | 8.48E-05 |
| GO:0015277~kainate selective glutamate receptor activity | 5 | 8.56E-08 | 1.00E-04 |
| GO:0007156~homophilic cell adhesion via plasma membrane adhesion molecules | 13 | 1.93E-07 | 2.78E-04 |
| GO:0007608~sensory perception of smell | 13 | 2.06E-07 | 2.97E-04 |
| GO:0030658~transport vesicle membrane | 8 | 2.59E-07 | 2.98E-04 |
| GO:0052689~carboxylic ester hydrolase activity | 8 | 3.33E-07 | 3.91E-04 |
| GO:0030669~clathrin-coated endocytic vesicle membrane | 8 | 4.47E-07 | 5.16E-04 |
| GO:0007215~glutamate receptor signaling pathway | 6 | 4.34E-07 | 6.24E-04 |
| GO:0005200~structural constituent of cytoskeleton | 11 | 5.79E-07 | 6.79E-04 |
| GO:0007565~female pregnancy | 10 | 6.13E-07 | 8.83E-04 |
| GO:0006955~immune response | 19 | 1.07E-06 | 0.002 |
| GO:0070062~extracellular exosome | 61 | 1.61E-06 | 0.002 |
| GO:0004971~AMPA glutamate receptor activity | 4 | 6.02E-06 | 0.007 |
| GO:0045211~postsynaptic membrane | 13 | 7.71E-06 | 0.009 |
| GO:0016045~detection of bacterium | 5 | 9.64E-06 | 0.014 |
| GO:0005231~excitatory extracellular ligand-gated ion channel activity | 4 | 1.49E-05 | 0.018 |
| GO:0007010~cytoskeleton organization | 11 | 1.25E-05 | 0.018 |
| GO:0042043~neurexin family protein binding | 5 | 1.58E-05 | 0.019 |
| GO:0002381~immunoglobulin production involved in immunoglobulin mediated immune response | 4 | 1.32E-05 | 0.019 |
| GO:0097110~scaffold protein binding | 7 | 1.80E-05 | 0.021 |
| GO:0045104~intermediate filament cytoskeleton organization | 5 | 1.81E-05 | 0.026 |
| GO:0042633~hair cycle | 5 | 1.81E-05 | 0.026 |
| GO:0008152~metabolic process | 11 | 1.81E-05 | 0.026 |
| GO:0009986~cell surface | 20 | 2.28E-05 | 0.026 |
| GO:0007158~neuron cell-cell adhesion | 5 | 2.39E-05 | 0.034 |
| GO:0006805~xenobiotic metabolic process | 8 | 2.49E-05 | 0.036 |
| GO:0050804~modulation of synaptic transmission | 5 | 3.10E-05 | 0.045 |

**Table S15: GO enrichment of genes that were expanded in Cnidaria compared to the common ancestor of Cnidaria and Bilateria.**

| Term | Count | *P*-value | FDR |
| --- | --- | --- | --- |
| GO:0005887~integral component of plasma membrane | 50 | 5.34E-23 | 5.93E-20 |
| GO:0005003~ephrin receptor activity | 11 | 1.89E-21 | 2.24E-18 |
| GO:0055085~transmembrane transport | 25 | 9.30E-21 | 1.38E-17 |
| GO:0005328~neurotransmitter:sodium symporter activity | 12 | 3.34E-20 | 3.95E-17 |
| GO:0043225~anion transmembrane-transporting ATPase activity | 10 | 1.46E-18 | 1.73E-15 |
| GO:0004062~aryl sulfotransferase activity | 10 | 5.82E-18 | 6.90E-15 |
| GO:0030286~dynein complex | 11 | 8.81E-17 | 1.22E-13 |
| GO:0003777~microtubule motor activity | 15 | 2.66E-16 | 2.66E-13 |
| GO:0050427~3'-phosphoadenosine 5'-phosphosulfate metabolic process | 10 | 1.86E-16 | 3.33E-13 |
| GO:0051923~sulfation | 9 | 9.29E-16 | 1.31E-12 |
| GO:0048013~ephrin receptor signaling pathway | 14 | 4.08E-14 | 6.05E-11 |
| GO:0005524~ATP binding | 40 | 9.95E-14 | 1.18E-10 |
| GO:0042626~ATPase activity, coupled to transmembrane movement of substances | 11 | 3.52E-13 | 4.17E-10 |
| GO:0098656~anion transmembrane transport | 10 | 4.88E-13 | 7.22E-10 |
| GO:0005886~plasma membrane | 64 | 5.83E-12 | 6.48E-09 |
| GO:0006836~neurotransmitter transport | 9 | 8.06E-12 | 1.19E-08 |
| GO:0016021~integral component of membrane | 72 | 1.35E-11 | 1.51E-08 |
| GO:0005858~axonemal dynein complex | 7 | 1.80E-11 | 2.01E-08 |
| GO:0007018~microtubule-based movement | 12 | 1.36E-11 | 2.01E-08 |
| GO:0016887~ATPase activity | 15 | 3.26E-11 | 3.86E-08 |
| GO:0008146~sulfotransferase activity | 9 | 1.36E-10 | 1.62E-07 |
| GO:0046920~alpha-(1->3)-fucosyltransferase activity | 6 | 8.08E-10 | 9.57E-07 |
| GO:0018108~peptidyl-tyrosine phosphorylation | 13 | 1.06E-09 | 1.57E-06 |
| GO:0008417~fucosyltransferase activity | 6 | 1.81E-09 | 2.14E-06 |
| GO:0005005~transmembrane-ephrin receptor activity | 6 | 1.81E-09 | 2.14E-06 |
| GO:0042355~L-fucose catabolic process | 6 | 4.38E-09 | 6.48E-06 |
| GO:0036065~fucosylation | 6 | 7.98E-09 | 1.18E-05 |
| GO:0060285~cilium-dependent cell motility | 6 | 7.98E-09 | 1.18E-05 |
| GO:0005874~microtubule | 15 | 2.68E-08 | 2.98E-05 |
| GO:0008202~steroid metabolic process | 8 | 2.20E-08 | 3.26E-05 |
| GO:0006810~transport | 16 | 3.06E-08 | 4.53E-05 |
| GO:0005004~GPI-linked ephrin receptor activity | 5 | 7.55E-08 | 8.94E-05 |
| GO:0050294~steroid sulfotransferase activity | 4 | 1.29E-06 | 0.002 |
| GO:0005332~gamma-aminobutyric acid:sodium symporter activity | 4 | 1.29E-06 | 0.002 |
| GO:0015812~gamma-aminobutyric acid transport | 4 | 3.61E-06 | 0.005 |
| GO:0006487~protein N-linked glycosylation | 6 | 8.46E-06 | 0.013 |
| GO:0008046~axon guidance receptor activity | 4 | 1.11E-05 | 0.013 |
| GO:0031290~retinal ganglion cell axon guidance | 5 | 9.14E-06 | 0.014 |
| GO:0009986~cell surface | 15 | 1.94E-05 | 0.022 |
| GO:0007156~homophilic cell adhesion via plasma membrane adhesion molecules | 9 | 1.88E-05 | 0.028 |
| GO:0009812~flavonoid metabolic process | 4 | 1.99E-05 | 0.029 |
| GO:0006814~sodium ion transport | 7 | 2.58E-05 | 0.038 |
| GO:0007155~cell adhesion | 14 | 2.69E-05 | 0.040 |
| GO:0005509~calcium ion binding | 17 | 3.49E-05 | 0.041 |
| GO:0006584~catecholamine metabolic process | 4 | 2.97E-05 | 0.044 |

**Table S16: GO enrichment of genes that were contracted in Scyphozoa compared to the common ancestor of Scyphozoa and Hydrozoa*.***

| Term | Count | *P*-value | FDR |
| --- | --- | --- | --- |
| GO:0000786~nucleosome | 48 | 1.85E-74 | 2.13E-71 |
| GO:0046982~protein heterodimerization activity | 50 | 3.41E-40 | 4.17E-37 |
| GO:0006334~nucleosome assembly | 33 | 1.02E-39 | 1.43E-36 |
| GO:0000788~nuclear nucleosome | 19 | 1.47E-26 | 1.69E-23 |
| GO:0006342~chromatin silencing | 19 | 2.93E-26 | 4.13E-23 |
| GO:0045653~negative regulation of megakaryocyte differentiation | 14 | 6.43E-24 | 9.06E-21 |
| GO:0003677~DNA binding | 59 | 6.05E-22 | 7.39E-19 |
| GO:0016233~telomere capping | 14 | 8.25E-22 | 1.16E-18 |
| GO:0006336~DNA replication-independent nucleosome assembly | 14 | 7.33E-21 | 1.03E-17 |
| GO:0032200~telomere organization | 14 | 1.40E-20 | 1.98E-17 |
| GO:0070062~extracellular exosome | 72 | 1.22E-19 | 1.41E-16 |
| GO:0006335~DNA replication-dependent nucleosome assembly | 14 | 2.33E-19 | 3.29E-16 |
| GO:0006352~DNA-templated transcription, initiation | 14 | 1.50E-18 | 2.12E-15 |
| GO:0000183~chromatin silencing at rDNA | 14 | 2.30E-18 | 3.24E-15 |
| GO:0006303~double-strand break repair via nonhomologous end joining | 16 | 3.98E-18 | 5.60E-15 |
| GO:0000228~nuclear chromosome | 15 | 9.21E-18 | 1.06E-14 |
| GO:0043225~anion transmembrane-transporting ATPase activity | 10 | 1.07E-17 | 1.31E-14 |
| GO:0051290~protein heterotetramerization | 14 | 1.59E-17 | 2.23E-14 |
| GO:0034080~CENP-A containing nucleosome assembly | 14 | 2.25E-17 | 3.18E-14 |
| GO:1904837~beta-catenin-TCF complex assembly | 14 | 2.25E-17 | 3.18E-14 |
| GO:0045815~positive regulation of gene expression, epigenetic | 15 | 1.22E-16 | 1.55E-13 |
| GO:0045814~negative regulation of gene expression, epigenetic | 14 | 2.07E-16 | 3.11E-13 |
| GO:0042393~histone binding | 17 | 7.15E-15 | 8.68E-12 |
| GO:0000784~nuclear chromosome, telomeric region | 17 | 1.64E-14 | 1.88E-11 |
| GO:0000790~nuclear chromatin | 19 | 4.74E-14 | 5.46E-11 |
| GO:0002227~innate immune response in mucosa | 10 | 3.54E-13 | 4.99E-10 |
| GO:0098656~anion transmembrane transport | 10 | 2.39E-12 | 3.37E-09 |
| GO:0042626~ATPase activity, coupled to transmembrane movement of substances | 11 | 3.09E-12 | 3.77E-09 |
| GO:0031047~gene silencing by RNA | 14 | 1.15E-11 | 1.62E-08 |
| GO:0044267~cellular protein metabolic process | 14 | 2.53E-11 | 3.56E-08 |
| GO:0019731~antibacterial humoral response | 10 | 1.07E-10 | 1.51E-07 |
| GO:0019904~protein domain specific binding | 16 | 3.22E-10 | 3.93E-07 |
| GO:0031012~extracellular matrix | 18 | 5.65E-10 | 6.51E-07 |
| GO:0050830~defense response to Gram-positive bacterium | 11 | 2.84E-09 | 4.00E-06 |
| GO:0005391~sodium:potassium-exchanging ATPase activity | 6 | 1.06E-08 | 1.30E-05 |
| GO:0036376~sodium ion export from cell | 6 | 1.05E-08 | 1.48E-05 |
| GO:0005615~extracellular space | 34 | 2.18E-08 | 2.51E-05 |
| GO:0030007~cellular potassium ion homeostasis | 6 | 3.26E-08 | 4.60E-05 |
| GO:0010248~establishment or maintenance of transmembrane electrochemical gradient | 6 | 5.27E-08 | 7.42E-05 |
| GO:0010107~potassium ion import | 7 | 1.16E-07 | 1.63E-04 |
| GO:0043234~protein complex | 17 | 4.32E-07 | 4.97E-04 |
| GO:0006883~cellular sodium ion homeostasis | 6 | 4.57E-07 | 6.44E-04 |
| GO:0016705~oxidoreductase activity, acting on paired donors, with incorporation or reduction of molecular oxygen | 8 | 5.51E-07 | 6.73E-04 |
| GO:0055078~sodium ion homeostasis | 5 | 1.63E-06 | 0.002 |
| GO:0004181~metallocarboxypeptidase activity | 6 | 3.02E-06 | 0.004 |
| GO:0008499~UDP-galactose:beta-N-acetylglucosamine beta-1,3-galactosyltransferase activity | 5 | 3.52E-06 | 0.004 |
| GO:0072562~blood microparticle | 10 | 5.83E-06 | 0.007 |
| GO:0090131~mesenchyme migration | 4 | 6.10E-06 | 0.009 |
| GO:0015991~ATP hydrolysis coupled proton transport | 6 | 7.23E-06 | 0.010 |
| GO:0031090~organelle membrane | 8 | 8.91E-06 | 0.010 |
| GO:0004497~monooxygenase activity | 7 | 1.01E-05 | 0.012 |
| GO:0001895~retina homeostasis | 6 | 2.24E-05 | 0.032 |
| GO:0015280~ligand-gated sodium channel activity | 4 | 3.37E-05 | 0.041 |
| GO:0006690~icosanoid metabolic process | 4 | 3.35E-05 | 0.047 |

**Table S17: GO enrichment of genes that were expanded in Scyphozoa compared to the common ancestor of Scyphozoa and Hydrozoa*.***

| Term | Count | *P*-value | FDR |
| --- | --- | --- | --- |
| GO:0008202~steroid metabolic process | 19 | 9.34E-24 | 1.47E-20 |
| GO:0008395~steroid hydroxylase activity | 16 | 5.52E-23 | 7.49E-20 |
| GO:0005887~integral component of plasma membrane | 64 | 1.46E-21 | 1.83E-18 |
| GO:0005506~iron ion binding | 26 | 1.47E-21 | 1.99E-18 |
| GO:0020037~heme binding | 24 | 3.44E-20 | 4.66E-17 |
| GO:0006805~xenobiotic metabolic process | 20 | 7.92E-20 | 1.25E-16 |
| GO:0005003~ephrin receptor activity | 11 | 4.97E-19 | 6.75E-16 |
| GO:0070330~aromatase activity | 14 | 1.12E-18 | 1.52E-15 |
| GO:0016705~oxidoreductase activity, acting on paired donors, with incorporation or reduction of molecular oxygen | 17 | 3.28E-18 | 4.45E-15 |
| GO:0004497~monooxygenase activity | 17 | 4.48E-18 | 6.07E-15 |
| GO:0005328~neurotransmitter:sodium symporter activity | 12 | 1.52E-17 | 2.06E-14 |
| GO:0019373~epoxygenase P450 pathway | 12 | 2.24E-17 | 3.54E-14 |
| GO:0004867~serine-type endopeptidase inhibitor activity | 19 | 7.54E-17 | 1.55E-13 |
| GO:0019825~oxygen binding | 15 | 1.72E-16 | 3.00E-13 |
| GO:0004062~aryl sulfotransferase activity | 10 | 8.55E-16 | 1.21E-12 |
| GO:0031090~organelle membrane | 17 | 3.34E-15 | 4.15E-12 |
| GO:0010951~negative regulation of endopeptidase activity | 19 | 8.62E-15 | 1.37E-11 |
| GO:0030286~dynein complex | 11 | 2.00E-14 | 2.49E-11 |
| GO:0008392~arachidonic acid epoxygenase activity | 10 | 1.89E-14 | 2.56E-11 |
| GO:0050427~3'-phosphoadenosine 5'-phosphosulfate metabolic process | 10 | 2.60E-14 | 4.10E-11 |
| GO:0051923~sulfation | 9 | 7.45E-14 | 1.17E-10 |
| GO:0006810~transport | 27 | 1.77E-13 | 2.79E-10 |
| GO:0003777~microtubule motor activity | 15 | 5.53E-13 | 7.50E-10 |
| GO:0016712~oxidoreductase activity, acting on paired donors, with incorporation or reduction of molecular oxygen, reduced flavin or flavoprotein as one donor, and incorporation of one atom of oxygen | 9 | 2.11E-12 | 2.86E-09 |
| GO:0005886~plasma membrane | 93 | 8.44E-12 | 1.05E-08 |
| GO:0005234~extracellular-glutamate-gated ion channel activity | 9 | 1.39E-11 | 1.88E-08 |
| GO:0048013~ephrin receptor signaling pathway | 14 | 4.11E-11 | 6.48E-08 |
| GO:0055114~oxidation-reduction process | 31 | 4.57E-11 | 7.21E-08 |
| GO:0005452~inorganic anion exchanger activity | 9 | 6.26E-11 | 8.49E-08 |
| GO:0004970~ionotropic glutamate receptor activity | 8 | 1.81E-10 | 2.46E-07 |
| GO:0008509~anion transmembrane transporter activity | 7 | 2.16E-10 | 2.93E-07 |
| GO:0035235~ionotropic glutamate receptor signaling pathway | 9 | 2.89E-10 | 4.56E-07 |
| GO:0005858~axonemal dynein complex | 7 | 4.53E-10 | 5.66E-07 |
| GO:0008331~high voltage-gated calcium channel activity | 7 | 5.34E-10 | 7.24E-07 |
| GO:0016887~ATPase activity | 17 | 6.04E-10 | 8.20E-07 |
| GO:0006836~neurotransmitter transport | 9 | 6.01E-10 | 9.49E-07 |
| GO:0017144~drug metabolic process | 9 | 8.46E-10 | 1.33E-06 |
| GO:0042738~exogenous drug catabolic process | 7 | 2.84E-09 | 4.47E-06 |
| GO:0005524~ATP binding | 46 | 4.42E-09 | 5.99E-06 |
| GO:0007018~microtubule-based movement | 12 | 4.37E-09 | 6.89E-06 |
| GO:0008146~sulfotransferase activity | 9 | 1.01E-08 | 1.37E-05 |
| GO:0051453~regulation of intracellular pH | 9 | 1.05E-08 | 1.65E-05 |
| GO:0046920~alpha-(1->3)-fucosyltransferase activity | 6 | 1.24E-08 | 1.68E-05 |
| GO:0016021~integral component of membrane | 99 | 1.47E-08 | 1.84E-05 |
| GO:0009308~amine metabolic process | 6 | 1.47E-08 | 2.32E-05 |
| GO:0005005~transmembrane-ephrin receptor activity | 6 | 2.76E-08 | 3.74E-05 |
| GO:0008417~fucosyltransferase activity | 6 | 2.76E-08 | 3.74E-05 |
| GO:0097267~omega-hydroxylase P450 pathway | 6 | 3.28E-08 | 5.18E-05 |
| GO:0018108~peptidyl-tyrosine phosphorylation | 14 | 5.48E-08 | 8.65E-05 |
| GO:0015701~bicarbonate transport | 9 | 5.64E-08 | 8.90E-05 |
| GO:0042355~L-fucose catabolic process | 6 | 6.50E-08 | 1.03E-04 |
| GO:0015277~kainate selective glutamate receptor activity | 5 | 9.66E-08 | 1.31E-04 |
| GO:0060285~cilium-dependent cell motility | 6 | 1.18E-07 | 1.86E-04 |
| GO:0036065~fucosylation | 6 | 1.18E-07 | 1.86E-04 |
| GO:0006600~creatine metabolic process | 6 | 1.18E-07 | 1.86E-04 |
| GO:0015301~anion:anion antiporter activity | 7 | 2.26E-07 | 3.06E-04 |
| GO:0004111~creatine kinase activity | 5 | 2.87E-07 | 3.90E-04 |
| GO:0007268~chemical synaptic transmission | 16 | 3.01E-07 | 4.74E-04 |
| GO:0016098~monoterpenoid metabolic process | 5 | 3.30E-07 | 5.21E-04 |
| GO:0002933~lipid hydroxylation | 5 | 3.30E-07 | 5.21E-04 |
| GO:0005004~GPI-linked ephrin receptor activity | 5 | 6.64E-07 | 9.01E-04 |
| GO:0005891~voltage-gated calcium channel complex | 7 | 8.53E-07 | 0.001 |
| GO:0007215~glutamate receptor signaling pathway | 6 | 7.37E-07 | 0.001 |
| GO:0009812~flavonoid metabolic process | 5 | 1.51E-06 | 0.002 |
| GO:0098656~anion transmembrane transport | 7 | 1.51E-06 | 0.002 |
| GO:0005789~endoplasmic reticulum membrane | 28 | 2.54E-06 | 0.003 |
| GO:0006584~catecholamine metabolic process | 5 | 2.69E-06 | 0.004 |
| GO:0002020~protease binding | 10 | 3.26E-06 | 0.004 |
| GO:0005874~microtubule | 16 | 3.61E-06 | 0.005 |
| GO:0035725~sodium ion transmembrane transport | 9 | 3.15E-06 | 0.005 |
| GO:0005509~calcium ion binding | 25 | 4.87E-06 | 0.007 |
| GO:0008510~sodium:bicarbonate symporter activity | 4 | 6.59E-06 | 0.009 |
| GO:0050294~steroid sulfotransferase activity | 4 | 6.59E-06 | 0.009 |
| GO:0004971~AMPA glutamate receptor activity | 4 | 6.59E-06 | 0.009 |
| GO:0005332~gamma-aminobutyric acid:sodium symporter activity | 4 | 6.59E-06 | 0.009 |
| GO:0034875~caffeine oxidase activity | 4 | 6.59E-06 | 0.009 |
| GO:0005245~voltage-gated calcium channel activity | 7 | 7.24E-06 | 0.010 |
| GO:0070989~oxidative demethylation | 5 | 1.03E-05 | 0.016 |
| GO:0005231~excitatory extracellular ligand-gated ion channel activity | 4 | 1.63E-05 | 0.022 |
| GO:0007155~cell adhesion | 19 | 1.59E-05 | 0.025 |
| GO:0015171~amino acid transmembrane transporter activity | 7 | 1.89E-05 | 0.026 |
| GO:0015812~gamma-aminobutyric acid transport | 4 | 1.81E-05 | 0.029 |
| GO:0030425~dendrite | 15 | 3.79E-05 | 0.047 |

**Table S18: GO enrichment of genes that were contracted in *Nemopilema* *nomurai* compared to the common ancestor of *Nemopilema* and *Aurelia.***

| Term | Count | *P*-value | FDR |
| --- | --- | --- | --- |
| GO:0000786~nucleosome | 51 | 6.83E-76 | 8.45E-73 |
| GO:0006334~nucleosome assembly | 36 | 2.17E-41 | 3.19E-38 |
| GO:0046982~protein heterodimerization activity | 52 | 2.73E-38 | 3.38E-35 |
| GO:0000788~nuclear nucleosome | 19 | 7.48E-25 | 9.26E-22 |
| GO:0006342~chromatin silencing | 19 | 1.74E-24 | 2.56E-21 |
| GO:0070062~extracellular exosome | 89 | 3.65E-24 | 4.52E-21 |
| GO:0045653~negative regulation of megakaryocyte differentiation | 14 | 1.20E-22 | 1.77E-19 |
| GO:0000183~chromatin silencing at rDNA | 17 | 1.47E-22 | 2.17E-19 |
| GO:0016233~telomere capping | 14 | 1.53E-20 | 2.25E-17 |
| GO:0045814~negative regulation of gene expression, epigenetic | 17 | 4.96E-20 | 7.30E-17 |
| GO:0045815~positive regulation of gene expression, epigenetic | 18 | 6.51E-20 | 9.59E-17 |
| GO:0006336~DNA replication-independent nucleosome assembly | 14 | 1.35E-19 | 1.99E-16 |
| GO:0032200~telomere organization | 14 | 2.58E-19 | 3.79E-16 |
| GO:0032982~myosin filament | 12 | 4.91E-19 | 6.08E-16 |
| GO:0016887~ATPase activity | 24 | 1.04E-18 | 1.29E-15 |
| GO:0006335~DNA replication-dependent nucleosome assembly | 14 | 4.24E-18 | 6.25E-15 |
| GO:0051290~protein heterotetramerization | 15 | 5.85E-18 | 8.61E-15 |
| GO:0003677~DNA binding | 59 | 1.58E-17 | 1.95E-14 |
| GO:0006352~DNA-templated transcription, initiation | 14 | 2.71E-17 | 3.99E-14 |
| GO:1904837~beta-catenin-TCF complex assembly | 14 | 4.00E-16 | 6.55E-13 |
| GO:0034080~CENP-A containing nucleosome assembly | 14 | 4.00E-16 | 6.55E-13 |
| GO:0006303~double-strand break repair via nonhomologous end joining | 15 | 3.47E-15 | 5.06E-12 |
| GO:0000228~nuclear chromosome | 14 | 6.68E-15 | 8.24E-12 |
| GO:0030286~dynein complex | 11 | 6.97E-15 | 8.66E-12 |
| GO:0042393~histone binding | 18 | 7.65E-15 | 9.48E-12 |
| GO:0005859~muscle myosin complex | 10 | 1.29E-14 | 1.61E-11 |
| GO:0015701~bicarbonate transport | 13 | 2.33E-14 | 3.42E-11 |
| GO:0031047~gene silencing by RNA | 17 | 4.78E-14 | 7.04E-11 |
| GO:0003777~microtubule motor activity | 15 | 7.53E-14 | 9.31E-11 |
| GO:0044267~cellular protein metabolic process | 17 | 1.28E-13 | 1.88E-10 |
| GO:0000146~microfilament motor activity | 10 | 1.63E-13 | 2.01E-10 |
| GO:0000784~nuclear chromosome, telomeric region | 17 | 4.44E-13 | 5.49E-10 |
| GO:0000790~nuclear chromatin | 19 | 1.83E-12 | 2.26E-09 |
| GO:0002227~innate immune response in mucosa | 10 | 2.56E-12 | 3.77E-09 |
| GO:0003774~motor activity | 13 | 2.98E-12 | 3.69E-09 |
| GO:0031012~extracellular matrix | 22 | 4.93E-12 | 6.10E-09 |
| GO:0005524~ATP binding | 45 | 1.06E-10 | 1.32E-07 |
| GO:0005858~axonemal dynein complex | 7 | 2.41E-10 | 2.98E-07 |
| GO:0019731~antibacterial humoral response | 10 | 7.47E-10 | 1.10E-06 |
| GO:0007018~microtubule-based movement | 12 | 9.42E-10 | 1.39E-06 |
| GO:0005516~calmodulin binding | 16 | 1.10E-09 | 1.37E-06 |
| GO:0006936~muscle contraction | 13 | 1.53E-09 | 2.25E-06 |
| GO:0016459~myosin complex | 10 | 2.09E-09 | 2.58E-06 |
| GO:0004089~carbonate dehydratase activity | 7 | 3.08E-09 | 3.82E-06 |
| GO:0030049~muscle filament sliding | 9 | 5.31E-09 | 7.81E-06 |
| GO:0050830~defense response to Gram-positive bacterium | 11 | 2.31E-08 | 3.40E-05 |
| GO:0019904~protein domain specific binding | 15 | 3.32E-08 | 4.11E-05 |
| GO:0008271~secondary active sulfate transmembrane transporter activity | 6 | 4.83E-08 | 5.97E-05 |
| GO:0015116~sulfate transmembrane transporter activity | 6 | 4.83E-08 | 5.97E-05 |
| GO:0019531~oxalate transmembrane transporter activity | 6 | 4.83E-08 | 5.97E-05 |
| GO:0019532~oxalate transport | 6 | 5.71E-08 | 8.41E-05 |
| GO:0060285~cilium-dependent cell motility | 6 | 5.71E-08 | 8.41E-05 |
| GO:1902358~sulfate transmembrane transport | 6 | 9.70E-08 | 1.43E-04 |
| GO:0030017~sarcomere | 8 | 1.39E-07 | 1.72E-04 |
| GO:0015106~bicarbonate transmembrane transporter activity | 6 | 3.03E-07 | 3.75E-04 |
| GO:0030016~myofibril | 7 | 3.69E-07 | 4.57E-04 |
| GO:0030048~actin filament-based movement | 6 | 7.26E-07 | 0.001 |
| GO:0005874~microtubule | 16 | 9.74E-07 | 0.001 |
| GO:0043234~protein complex | 18 | 1.57E-06 | 0.002 |
| GO:0051453~regulation of intracellular pH | 7 | 2.02E-06 | 0.003 |
| GO:0015301~anion:anion antiporter activity | 6 | 3.18E-06 | 0.004 |
| GO:0030898~actin-dependent ATPase activity | 5 | 7.25E-06 | 0.009 |
| GO:0090131~mesenchyme migration | 4 | 1.17E-05 | 0.017 |
| GO:0006730~one-carbon metabolic process | 6 | 1.49E-05 | 0.022 |
| GO:0001725~stress fiber | 7 | 2.02E-05 | 0.025 |
| GO:0004035~alkaline phosphatase activity | 4 | 2.10E-05 | 0.026 |
| GO:0003779~actin binding | 13 | 3.21E-05 | 0.040 |
| GO:0015108~chloride transmembrane transporter activity | 4 | 3.65E-05 | 0.045 |

**Table S19: GO enrichment of genes that were expanded in *Nemopilema nomurai* compared to the common ancestor of *Nemopilema* and *Aurelia.***

| Term | Count | *P*-value | FDR |
| --- | --- | --- | --- |
| GO:0005328~neurotransmitter:sodium symporter activity | 17 | 1.13E-29 | 1.51E-26 |
| GO:0005887~integral component of plasma membrane | 65 | 3.61E-23 | 4.37E-20 |
| GO:0005003~ephrin receptor activity | 11 | 4.49E-19 | 6.00E-16 |
| GO:0006836~neurotransmitter transport | 14 | 6.72E-19 | 1.03E-15 |
| GO:0009813~flavonoid biosynthetic process | 13 | 7.60E-19 | 1.17E-15 |
| GO:0004222~metalloendopeptidase activity | 21 | 2.96E-18 | 3.96E-15 |
| GO:0052696~flavonoid glucuronidation | 13 | 3.81E-18 | 5.87E-15 |
| GO:0043225~anion transmembrane-transporting ATPase activity | 10 | 1.97E-16 | 3.00E-13 |
| GO:0015020~glucuronosyltransferase activity | 13 | 2.21E-16 | 3.00E-13 |
| GO:0005886~plasma membrane | 100 | 3.21E-16 | 4.00E-13 |
| GO:0004062~aryl sulfotransferase activity | 10 | 7.80E-16 | 1.03E-12 |
| GO:0008202~steroid metabolic process | 14 | 1.95E-15 | 3.08E-12 |
| GO:0050427~3'-phosphoadenosine 5'-phosphosulfate metabolic process | 10 | 2.07E-14 | 3.20E-11 |
| GO:0008237~metallopeptidase activity | 16 | 2.86E-14 | 3.81E-11 |
| GO:0005578~proteinaceous extracellular matrix | 24 | 3.65E-14 | 4.42E-11 |
| GO:0051923~sulfation | 9 | 6.10E-14 | 9.40E-11 |
| GO:0006805~xenobiotic metabolic process | 15 | 4.40E-13 | 6.78E-10 |
| GO:0098656~anion transmembrane transport | 11 | 1.24E-12 | 1.91E-09 |
| GO:0055085~transmembrane transport | 22 | 1.82E-12 | 2.80E-09 |
| GO:0016021~integral component of membrane | 106 | 2.23E-12 | 2.70E-09 |
| GO:0070006~metalloaminopeptidase activity | 10 | 2.60E-12 | 3.47E-09 |
| GO:0048013~ephrin receptor signaling pathway | 14 | 3.02E-11 | 4.66E-08 |
| GO:0006508~proteolysis | 28 | 6.48E-11 | 9.98E-08 |
| GO:0042626~ATPase activity, coupled to transmembrane movement of substances | 11 | 7.24E-11 | 9.66E-08 |
| GO:0015824~proline transport | 7 | 7.72E-11 | 1.19E-07 |
| GO:0015816~glycine transport | 7 | 2.29E-10 | 3.53E-07 |
| GO:0016758~transferase activity, transferring hexosyl groups | 9 | 1.52E-09 | 2.03E-06 |
| GO:0015171~amino acid transmembrane transporter activity | 10 | 3.39E-09 | 4.53E-06 |
| GO:0035524~proline transmembrane transport | 6 | 4.94E-09 | 7.61E-06 |
| GO:0016042~lipid catabolic process | 12 | 5.70E-09 | 8.78E-06 |
| GO:0006865~amino acid transport | 9 | 6.80E-09 | 1.05E-05 |
| GO:0008146~sulfotransferase activity | 9 | 9.33E-09 | 1.25E-05 |
| GO:0009986~cell surface | 25 | 1.01E-08 | 1.22E-05 |
| GO:0046920~alpha-(1->3)-fucosyltransferase activity | 6 | 1.18E-08 | 1.57E-05 |
| GO:0003333~amino acid transmembrane transport | 8 | 1.31E-08 | 2.02E-05 |
| GO:0005229~intracellular calcium activated chloride channel activity | 7 | 1.81E-08 | 2.41E-05 |
| GO:0052695~cellular glucuronidation | 7 | 2.04E-08 | 3.14E-05 |
| GO:0004435~phosphatidylinositol phospholipase C activity | 8 | 2.07E-08 | 2.76E-05 |
| GO:0008417~fucosyltransferase activity | 6 | 2.63E-08 | 3.51E-05 |
| GO:0005005~transmembrane-ephrin receptor activity | 6 | 2.63E-08 | 3.51E-05 |
| GO:0031090~organelle membrane | 11 | 4.69E-08 | 5.66E-05 |
| GO:0042355~L-fucose catabolic process | 6 | 5.75E-08 | 8.86E-05 |
| GO:0006600~creatine metabolic process | 6 | 1.04E-07 | 1.61E-04 |
| GO:0036065~fucosylation | 6 | 1.04E-07 | 1.61E-04 |
| GO:0004111~creatine kinase activity | 5 | 2.76E-07 | 3.69E-04 |
| GO:0015193~L-proline transmembrane transporter activity | 5 | 2.76E-07 | 3.69E-04 |
| GO:0018108~peptidyl-tyrosine phosphorylation | 13 | 3.32E-07 | 5.11E-04 |
| GO:0005004~GPI-linked ephrin receptor activity | 5 | 6.38E-07 | 8.52E-04 |
| GO:0009308~amine metabolic process | 5 | 1.37E-06 | 0.002 |
| GO:0005506~iron ion binding | 12 | 2.01E-06 | 0.003 |
| GO:0031290~retinal ganglion cell axon guidance | 6 | 2.43E-06 | 0.004 |
| GO:0006584~catecholamine metabolic process | 5 | 2.44E-06 | 0.004 |
| GO:0005524~ATP binding | 39 | 4.56E-06 | 0.006 |
| GO:0043171~peptide catabolic process | 6 | 5.34E-06 | 0.008 |
| GO:0034113~heterotypic cell-cell adhesion | 6 | 5.34E-06 | 0.008 |
| GO:0005332~gamma-aminobutyric acid:sodium symporter activity | 4 | 6.40E-06 | 0.009 |
| GO:0050294~steroid sulfotransferase activity | 4 | 6.40E-06 | 0.009 |
| GO:0061588~calcium activated phospholipid scrambling | 4 | 6.80E-06 | 0.010 |
| GO:0008305~integrin complex | 6 | 1.11E-05 | 0.013 |
| GO:0007160~cell-matrix adhesion | 9 | 1.27E-05 | 0.020 |
| GO:0004177~aminopeptidase activity | 6 | 1.42E-05 | 0.019 |
| GO:0015812~gamma-aminobutyric acid transport | 4 | 1.68E-05 | 0.026 |
| GO:0061591~calcium activated galactosylceramide scrambling | 4 | 1.68E-05 | 0.026 |
| GO:0061590~calcium activated phosphatidylcholine scrambling | 4 | 1.68E-05 | 0.026 |
| GO:0006810~transport | 16 | 2.13E-05 | 0.033 |
| GO:0007229~integrin-mediated signaling pathway | 9 | 2.56E-05 | 0.039 |

**Table S20: GO enrichment of genes that were specific to scyphozoan lineage.**

| Term | Count | *P*-value | FDR |
| --- | --- | --- | --- |
| GO:0005216~ion channel activity | 47 | 1.80E-11 | 4.33E-09 |
| GO:0022838~substrate-specific channel activity | 47 | 1.80E-11 | 4.33E-09 |
| GO:0022803~passive transmembrane transporter activity | 47 | 2.71E-11 | 4.33E-09 |
| GO:0015267~channel activity | 47 | 2.71E-11 | 4.33E-09 |
| GO:0006811~ion transport | 45 | 1.32E-10 | 1.69E-08 |
| GO:0015318~inorganic molecular entity transmembrane transporter activity | 55 | 9.10E-10 | 9.69E-08 |
| GO:0015075~ion transmembrane transporter activity | 55 | 6.77E-09 | 6.18E-07 |
| GO:0005230~extracellular ligand-gated ion channel activity | 26 | 1.40E-08 | 1.12E-06 |
| GO:0022857~transmembrane transporter activity | 61 | 1.84E-08 | 1.31E-06 |
| GO:0015276~ligand-gated ion channel activity | 26 | 3.81E-08 | 2.21E-06 |
| GO:0022834~ligand-gated channel activity | 26 | 3.81E-08 | 2.21E-06 |
| GO:0022839~ion gated channel activity | 26 | 2.78E-07 | 1.48E-05 |
| GO:0022836~gated channel activity | 26 | 3.31E-07 | 1.63E-05 |
| GO:0005215~transporter activity | 62 | 4.96E-07 | 2.26E-05 |
| GO:0006810~transport | 74 | 1.11E-06 | 4.73E-05 |
| GO:0051234~establishment of localization | 74 | 1.29E-06 | 5.15E-05 |
| GO:0051179~localization | 74 | 2.63E-06 | 9.89E-05 |
| GO:0016020~membrane | 82 | 2.53E-05 | 8.98E-04 |
| GO:0046983~protein dimerization activity | 24 | 3.51E-05 | 1.18E-03 |
| GO:0055085~transmembrane transport | 36 | 6.45E-05 | 2.06E-03 |
| GO:0006541~glutamine metabolic process | 4 | 7.91E-05 | 2.41E-03 |
| GO:0051260~protein homooligomerization | 10 | 1.27E-04 | 3.69E-03 |
| GO:0051259~protein complex oligomerization | 10 | 1.73E-04 | 4.81E-03 |
| GO:0006814~sodium ion transport | 8 | 2.41E-04 | 6.26E-03 |
| GO:0009064~glutamine family amino acid metabolic process | 4 | 2.45E-04 | 6.26E-03 |
| GO:0005272~sodium channel activity | 8 | 5.65E-04 | 1.39E-02 |
| GO:1901605~alpha-amino acid metabolic process | 4 | 1.15E-03 | 2.72E-02 |
| GO:0065003~protein-containing complex assembly | 12 | 1.70E-03 | 3.81E-02 |
| GO:0008066~glutamate receptor activity | 8 | 1.97E-03 | 3.81E-02 |
| GO:0030594~neurotransmitter receptor activity | 8 | 1.97E-03 | 3.81E-02 |
| GO:0022824~transmitter-gated ion channel activity | 8 | 1.97E-03 | 3.81E-02 |
| GO:0022835~transmitter-gated channel activity | 8 | 1.97E-03 | 3.81E-02 |
| GO:0004970~ionotropic glutamate receptor activity | 8 | 1.97E-03 | 3.81E-02 |
| GO:0043933~protein-containing complex subunit organization | 12 | 2.37E-03 | 4.45E-02 |

**4.3 Protein domain expansions**

A protein domain is a conserved unit of a given protein sequence. The domain regions were predicted from the protein sequences using the InterProScan-5.13.52.0 program [56] with ProDom, Hamap, SMART, SUPERFAMILY, PRINTS, PANTHER, Gene3D, PIRSF, Pfam, ProSiteProfiles, TIGRFAM, ProSitePatterns, and Coils databases (Additional file 2: Table S21). To identify protein domains that are specifically expanded in the *Nemopilema* lineage, we conducted Fisher’s exact test for Pfam categories comparing in-group counts (*Nemopilema*) to average counts in the outgroups (all other species in the analysis). This test was iterated over all domains, and the *P*-values obtained were corrected with a 5% false discovery rate (FDR) to identify the significantly expanded domains in *Nemopilema*. To visualize these expanded domains, counts were normalized by Z-score (row) and significantly expanded domains were plotted using the heatmap function in R. We found 20 significantly expanded protein domains in the *Nemopilema* genome. Among them, Astacin (PF01400) and CUB (PF00431) domains are known to be associated with activation of growth factors [79] and regulating development [80], respectively (Figure S8). Also expanded in *Nemopilema* is the ShK domain-like (PF01549), which is related to Cnidaria toxin [81]*.* These expanded domains were also abundantly found in the previously published *Aurelia aurita* transcriptome study [82].

**
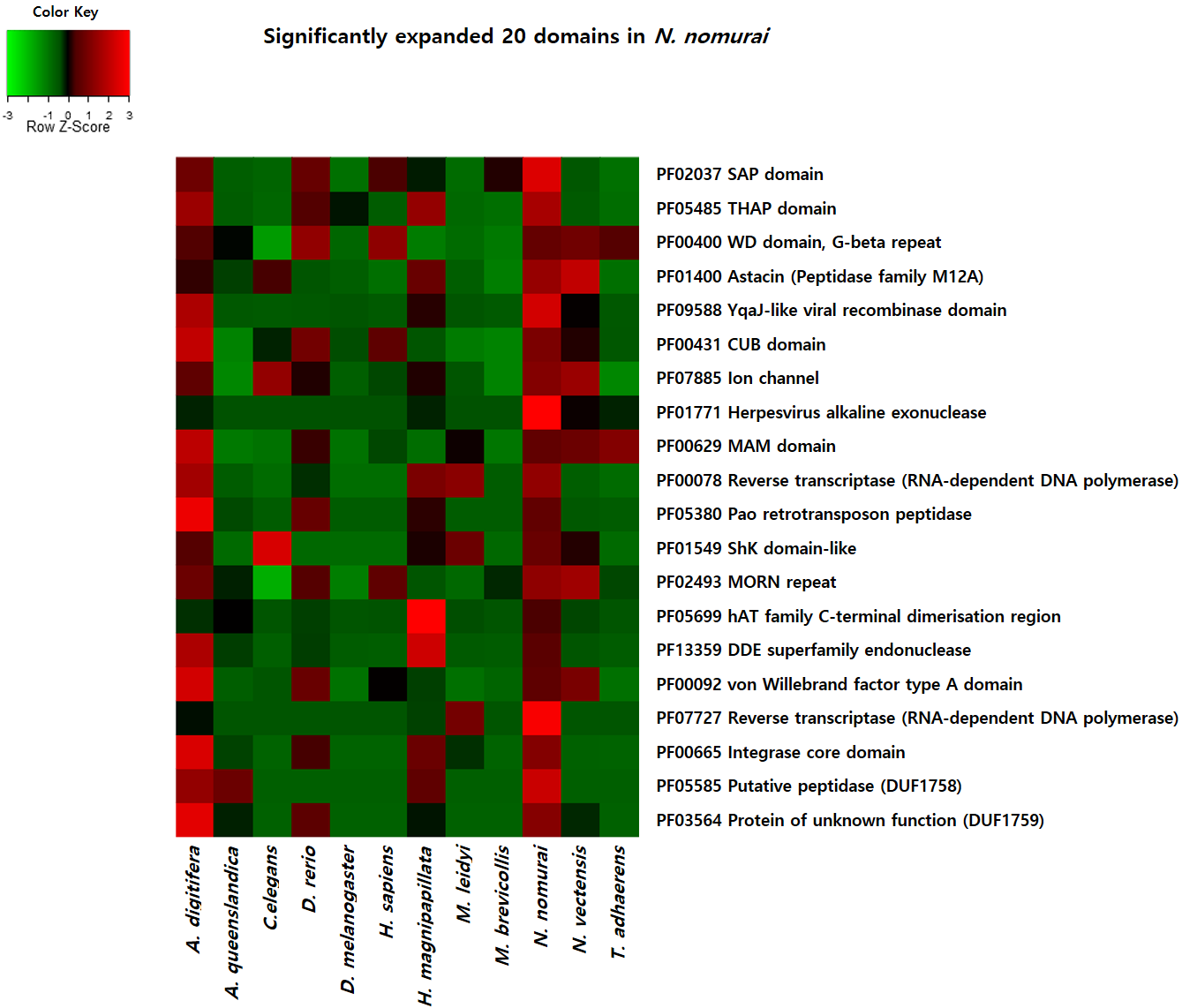
**

**Figure S8: Expanded domains in *Nemopilema nomurai* based on Pfam domain annotation.**

**4.4 Fast-evolving genes**

To identify fast-evolving genes, which show elevated substitution rates in their amino-acid sequences, we used the orthologous gene clusters (see Additional file 1: 4.1) and required each of the sequences from *Nemopilema*, *Acropora*, *Nematostella*, *Hydra*, and human (as an out-group) to be present in the same cluster. We filtered out the clusters that contained multi-copy genes in cnidarians. We conducted multiple sequence alignments using MUSCLE [42] and inferred maximum-likelihood phylogenies using the generalized time-reversible (GTR) model [83] in the program FastTree [62]. We identified 177 gene families which showed elevated substitution rates in *Nemopilema* amino-acid sequences (Additional file 3: Table S22). Functional analyses of these genes revealed that RNA processing related functions were enriched in *Nemopilema* (Table S23). RNA processing events allow a diverse array of proteins to be synthesized from a single gene and contributes to the evolution and the diversity of most eukaryotic organisms [84]. Additionally, the *MYL6B* gene in *Nemopilema* showed the most elevated substitution rate (Figure S9), which is related to smooth muscle and non-muscle cells and is highly conserved in mammalian species [85].

**
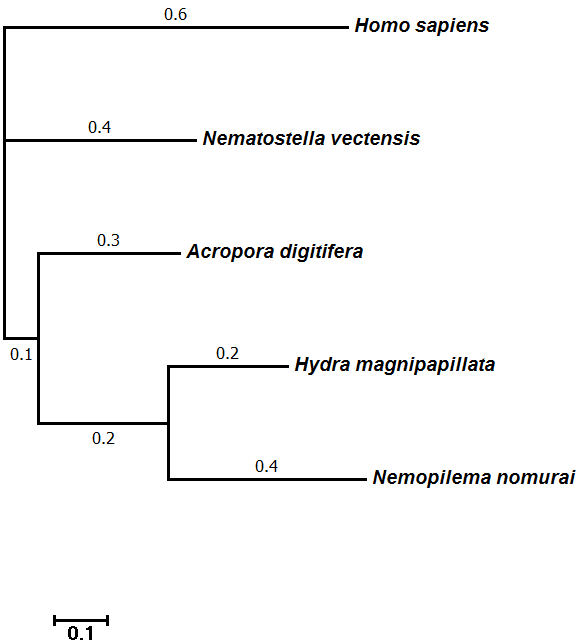
**

**Figure S9: Phylogenetic tree of the fast-evolving *MYL6B* gene in *Nemopilema nomurai*.** The scale bar indicates the branch lengths measured in the number of amino acid substitutions per site.

**Table S23: GO enrichment that were fast-evolving genes in *Nemopilema nomurai.***

| Term | Count | *P*-value | FDR |
| --- | --- | --- | --- |
| GO:0003723~RNA binding | 27 | 5.10E-09 | 6.69E-06 |
| GO:0070013~intracellular organelle lumen | 41 | 5.16E-09 | 6.52E-06 |
| GO:0043233~organelle lumen | 41 | 1.00E-08 | 1.26E-05 |
| GO:0031974~membrane-enclosed lumen | 41 | 1.76E-08 | 2.22E-05 |
| GO:0031981~nuclear lumen | 34 | 1.46E-07 | 1.84E-04 |
| GO:0030529~ribonucleoprotein complex | 18 | 2.31E-06 | 2.92E-03 |
| GO:0006396~RNA processing | 19 | 6.63E-06 | 1.03E-02 |
| GO:0016071~mRNA metabolic process | 15 | 1.64E-05 | 2.55E-02 |
| GO:0005654~nucleoplasm | 21 | 7.28E-05 | 9.20E-02 |
| GO:0006397~mRNA processing | 13 | 7.64E-05 | 1.19E-01 |
| GO:0008380~RNA splicing | 12 | 1.12E-04 | 1.75E-01 |
| GO:0005730~nucleolus | 18 | 1.16E-04 | 1.46E-01 |
| GO:0006351~transcription, DNA-dependent | 12 | 1.44E-04 | 2.23E-01 |
| GO:0032774~RNA biosynthetic process | 12 | 1.62E-04 | 2.51E-01 |
| GO:0043228~non-membrane-bounded organelle | 40 | 2.35E-04 | 2.97E-01 |
| GO:0043232~intracellular non-membrane-bounded organelle | 40 | 2.35E-04 | 2.97E-01 |
| GO:0006412~translation | 12 | 4.22E-04 | 6.54E-01 |
| GO:0006366~transcription from RNA polymerase II promoter | 10 | 5.04E-04 | 7.81E-01 |
| GO:0005643~nuclear pore | 6 | 6.36E-04 | 8.01E-01 |
| GO:0000377~RNA splicing, via transesterification reactions with bulged adenosine as nucleophile | 8 | 7.82E-04 | 1.21E+00 |
| GO:0000375~RNA splicing, via transesterification reactions | 8 | 7.82E-04 | 1.21E+00 |
| GO:0000398~nuclear mRNA splicing, via spliceosome | 8 | 7.82E-04 | 1.21E+00 |
| GO:0005829~cytosol | 24 | 1.06E-03 | 1.33E+00 |
| GO:0044451~nucleoplasm part | 14 | 1.12E-03 | 1.41E+00 |
| GO:0012505~endomembrane system | 17 | 1.26E-03 | 1.58E+00 |
| GO:0046930~pore complex | 6 | 1.46E-03 | 1.83E+00 |
| GO:0000502~proteasome complex | 5 | 1.99E-03 | 2.48E+00 |
| GO:0006414~translational elongation | 6 | 3.18E-03 | 4.82E+00 |
| GO:0000178~exosome (RNase complex) | 3 | 4.74E-03 | 5.83E+00 |
| GO:0016591~DNA-directed RNA polymerase II, holoenzyme | 5 | 5.54E-03 | 6.78E+00 |
| GO:0000175~3'-5'-exoribonuclease activity | 3 | 5.88E-03 | 7.44E+00 |
| GO:0004532~exoribonuclease activity | 3 | 6.90E-03 | 8.68E+00 |
| GO:0016896~exoribonuclease activity, producing 5'-phosphomonoesters | 3 | 6.90E-03 | 8.68E+00 |

**4.5 Gene age estimation**

Phylostratigraphy employs BLASTP-scored sequence similarity to estimate the minimal age of every protein-coding gene. The protein sequence is used to query the NCBI non-redundant database and detect the most distant species in which a sufficiently similar sequence is present, and positing that the gene is at least as old as the age of the common ancestor [50]. For every species, we use the NCBI taxonomy. The timing of most divergence events is estimated using TimeTree [51] and the Encyclopedia of Life [52]. To facilitate detection of sequence similarity, we use the e-value threshold of 10^-3^. We evaluate the age of all proteins whose length is equal or greater than 40 amino-acids. First, we counted the number of genes in each phylostratum, from most ancient (PS 1) to most recent (PS 11). Most genes are ancient (PS 1-2) and a substantial number appear young (PS 11; Figure S10). The large number of young PS 11 genes may in part reflect the small number of closely related species with sequenced genomes. If more closely related species were fully sequenced, PS 11 genes may become more evenly distributed between PS 8-11. Second, to understand broad evolutionary patterns, we aggregated the counts from several phylostrata into 3 broad evolutionary eras: ancient (PS 1-5, cellular organisms to Eumetazoa, 4,204 million years ago (Mya) - 741 Mya), middle (PS 6-7, Cnidaria to Scyphozoa, 741 Mya - 239 Mya) and young (PS 8-11, Rhizostomeae to *Nemopilema nomurai*, 239 Mya to present).


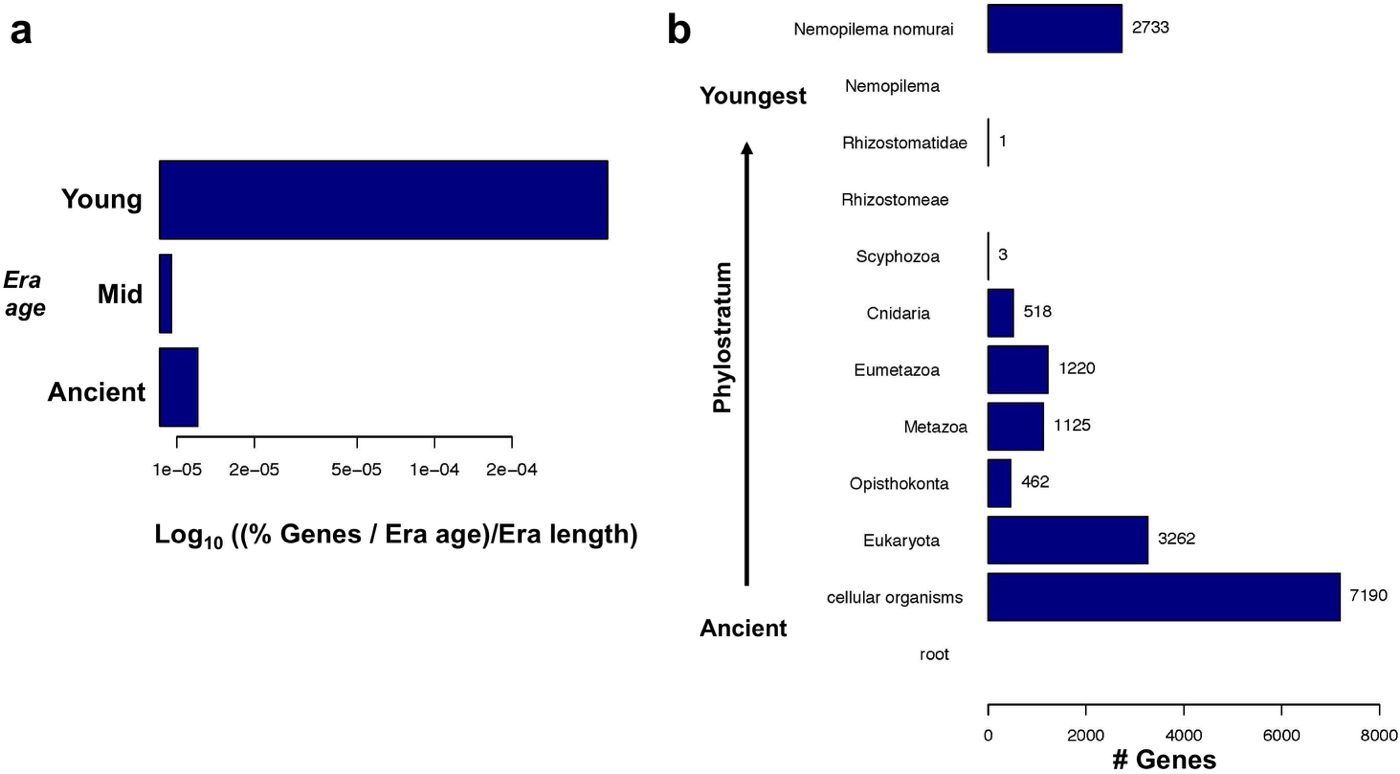


**Figure S10: Gene age estimation for *Nemopilema nomurai*. a**, When accounting for the age and length of evolutionary eras (average era ages: ancient, ~1,877 Mya; mid, ~659 Mya; young, ~147 Mya), the number of genes in every era increases steadily as the genes are more recent, suggesting gene turnover is highest in recent ages. **b**, The number of genes in every phylostratum, from most ancient to youngest, shows that most genes in the genome of Nomura’s jellyfish are ancient (7,190 in all cellular organisms and 3,262 in Eukaryota). The relatively large number of genes that appear species-specific (2,733) likely reflects the paucity of sequenced genomes since the emergence of Cnidaria.

**5 Genomic and transcriptomic signatures of jellyfish mobility**

The development of muscle contractions is one of the most important and distinctive adaptations of Eumetazoans. Cnidarian muscles are involved in various activities in close association with the nervous system [20]. Jellyfish possess a unique form of mobility and propel by contracting the striated muscle of the subumbrella. Bell contractions are regulated by a complex nervous system, including neural nets and concentrations of nerve cells at the bell margin (nerve rings) [86].

Among cnidarians, the medusae of Scyphozoa and its sister-group Cubozoa uniquely possess rhopalia at their bell margin. Rhopalia are small sensory structures to sense light (ocelli) and perceive gravity (statoliths), as well as controlling the pace of swimming-muscle contraction [87]. A previous study proposed that rhopalia evolved from preexisting sensory structures through the differential expression of POU genes within Otx oral-neuroectodermal domains across distinct populations of sensory cells [88]. Furthermore, Otx is expressed in developing tentacles of the anthozoan *Nematostella vectensis* [89]. To further explore the role of these genes in the jellyfish, we compared Otx and POU genes from three scyphozoans (*Nemopilema*, *Aurelia sp.1*, and *Sanderia malayensis*) and other cnidarians (*Nematostella*, *Hydra*, and *Acropora*). *Nemopilema* has two Otx and four POU genes that clustered with those of other cnidarians (Figures S11 and S12). Of these, *OtxA* shows an extremely conserved homeodomain among scyphozoans, while anthozoans and hydrozoans show relatively different pattern (Figure S11). In *Sanderia*, the expression levels of *OtxA* gradually increased from the polyp to the ephyra. Furthermore, *OtxA* was also expressed in both the oral arms and bell from the *Sanderia* medusa, but there was no observed expression in the tentacle (Table S24). Together, these findings suggest that Otx gene may play an evolutionarily important role in developing rhopalia in scyphozoans.


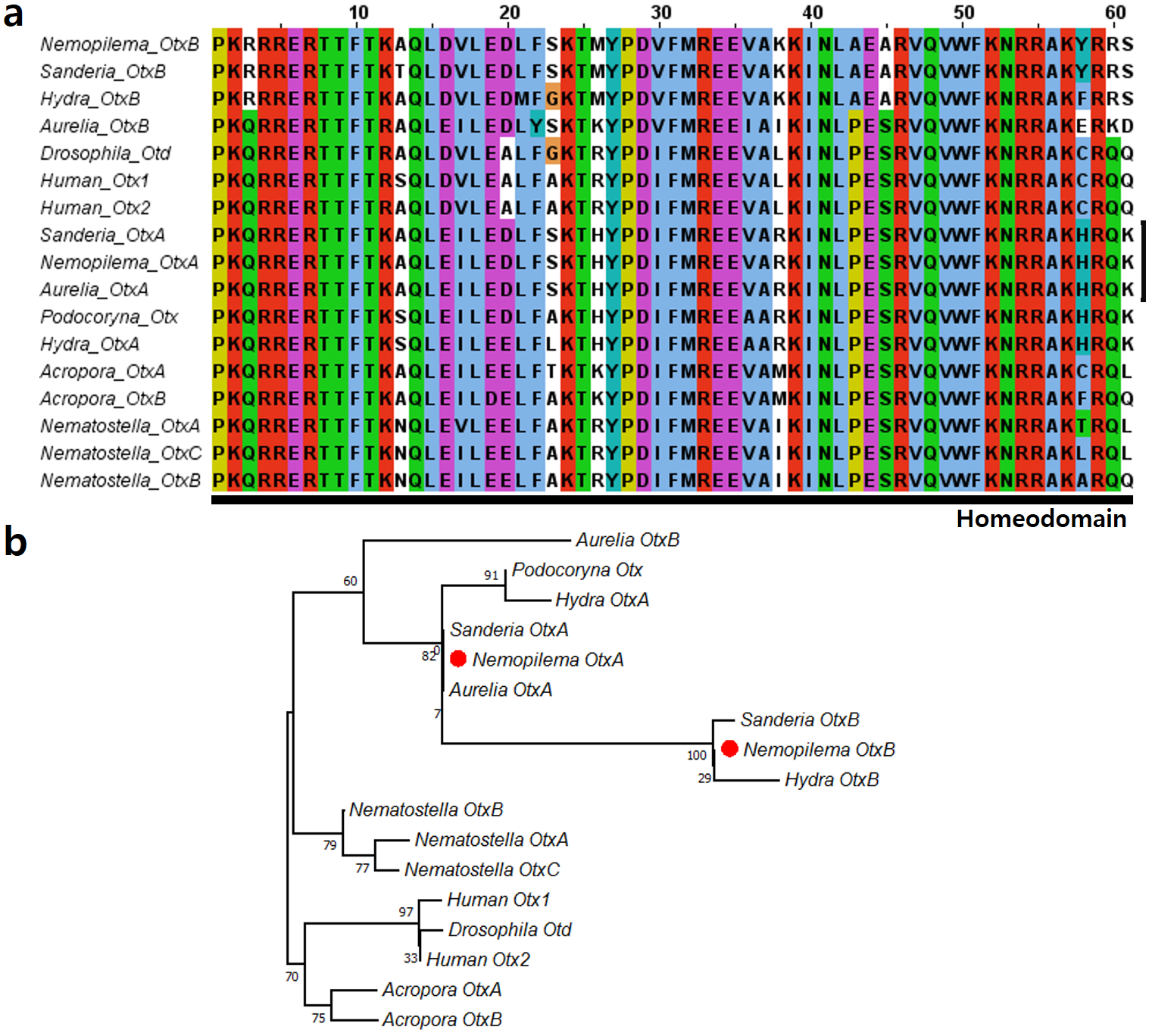


**Figure S11: Analysis of the Otx gene family protein sequences*.* a***,* Partial Otx protein sequence alignment with cnidarians, human and *drosophila.* The black line indicates the sites that correspond to the homeodomain. **b**, Maximum likelihood phylogenetic tree of Otx gene family. Numbers on nodes denote bootstrap values based on 100 replicates.


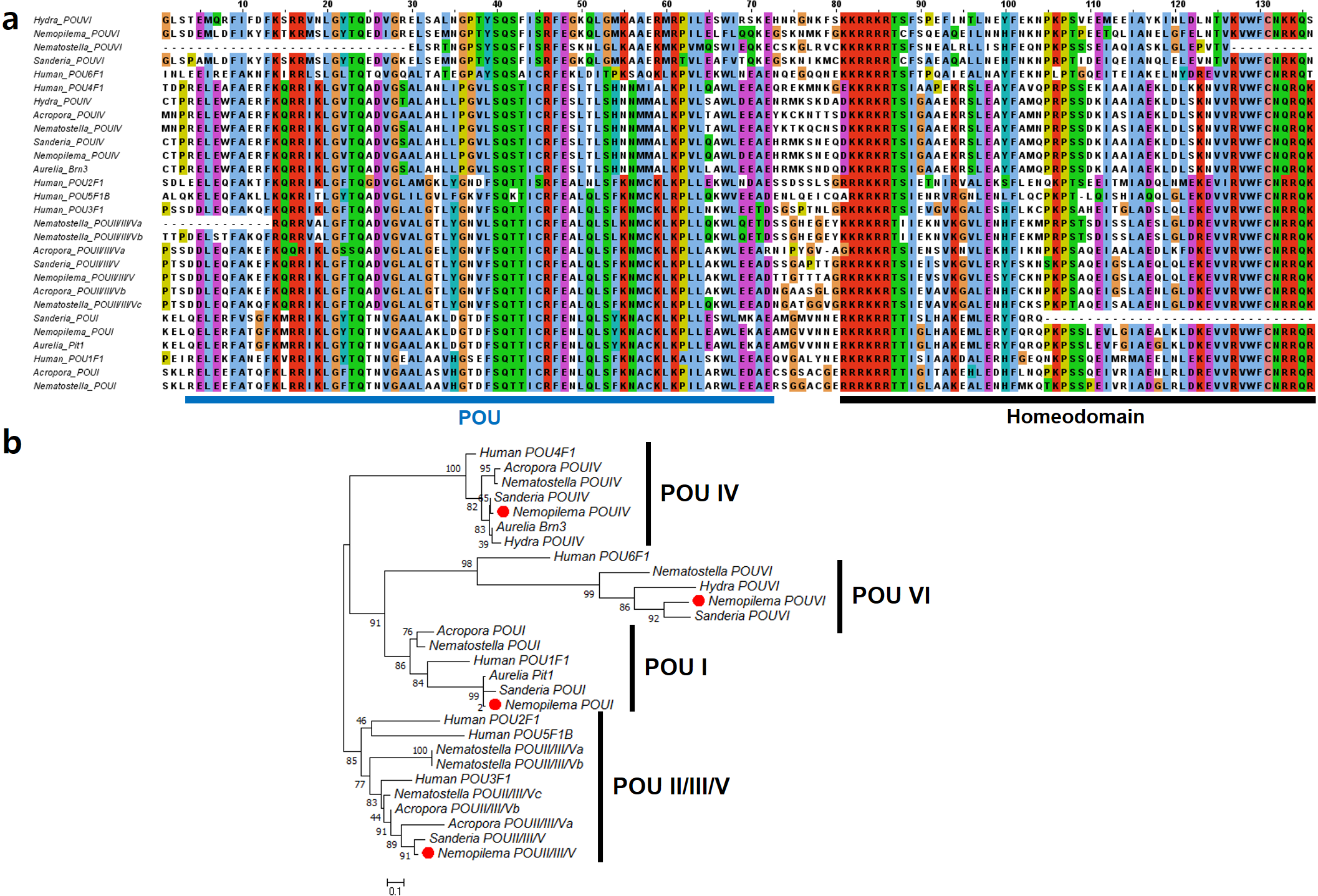


**Figure S12: Analysis of the POU gene family protein sequences*.* a***,* Partial POU protein sequence alignment with cnidarians, human and *drosophila.* The black line indicates the sites that correspond to the homeodomain, and the blue line corresponds to the POU domain (POU). **b**, Maximum likelihood phylogenetic tree of POU gene family. Numbers on nodes denote bootstrap values based on 100 replicates.

**Table S24: Expression values of Otx and POU genes in *S. malayensis* transcriptome.** The unit of expression values is FPKM (Fragment Per Kilobase of transcript per Million mapped reads).

| Gene | Polyp | Strobila | Ephyra | Medusa | | | |
| --- | --- | --- | --- | --- | --- | --- | --- |
|  |  |  |  | Bell | Tentacles | Oral arms | Merged |
| *OtxA* | 225.1 | 975.5 | 1168.0 | 833.4 | 0.0 | 529.6 | 502.4 |
| *OtxB* | 1247.0 | 2419.1 | 1891.4 | 1638.7 | 3956.0 | 44.7 | 2010.1 |
| *POUI* | 105.7 | 59.3 | 56.8 | 62.6 | 102.9 | 48.5 | 79.6 |
| *POUII/III/V* | 96.1 | 327.7 | 223.5 | 323.6 | 249.2 | 642.9 | 136.9 |
| *POUIV* | 565.4 | 231.7 | 284.0 | 150.3 | 558.0 | 341.0 | 402.4 |
| *POUVI* | 264.8 | 214.5 | 192.1 | 115.0 | 115.7 | 128.6 | 143.3 |

**5.1 GC content at third-codon positions**

Guanine and cytosine content at the third codon position (GC3) can be used as a good indicator of codon bias [14]. Amino acids encoded by codons having G or C in the third position appear more tightly linked to cell function and survival than are those encoded by codons having A or T as third position [90]. Thus, we calculated GC3 from eleven metazoans and one unicellular holozoan gene-sets (Figure S13). We extracted 100 and 500 genes with the top and bottom GC3 ranks from each genome. A functional enrichment test was then conducted using the ClueGO application v2.3.2 [91] of the Cytoscape platform v3.4.0 [92]. We compared the GO terms from *Nemopilema* with those of the other eleven species to find *Nemopilema* specific functions. *Nemopilema* shows high-scoring top 100 and 500 GC3 biased genes that include enriched terms for muscle contraction, neuropeptide signaling pathway, homeostasis, and renal system (Additional file 4: Tables S25-S28). The elevated codon bias in these systems in *Nemopilema* may be related to its active mobility and rapid osmotic adaptation to local aquatic environments. This is critical for jellyfish, which migrate diurnally across depths and travel rather passively, but extensively, through environments with varying levels of salinity. Although jellyfish are osmoconformers, which maintain an internal environment that is isotonic to their external environment, they also need to generate ion gradients [93]. Therefore, we speculate that they have evolved to maintain homeostasis of ion gradients that are essential for the activation of muscle contractions that power the jellyfish’s mobile predation, by efficiently minimizing the osmotic gradient onto a given external environment. Taken together, jellyfish have an interesting combination of active mobility, neuronal signal processing, and a relatively rapid osmosis response that have combined to allow it to colonize diverse and highly variable aquatic environments.


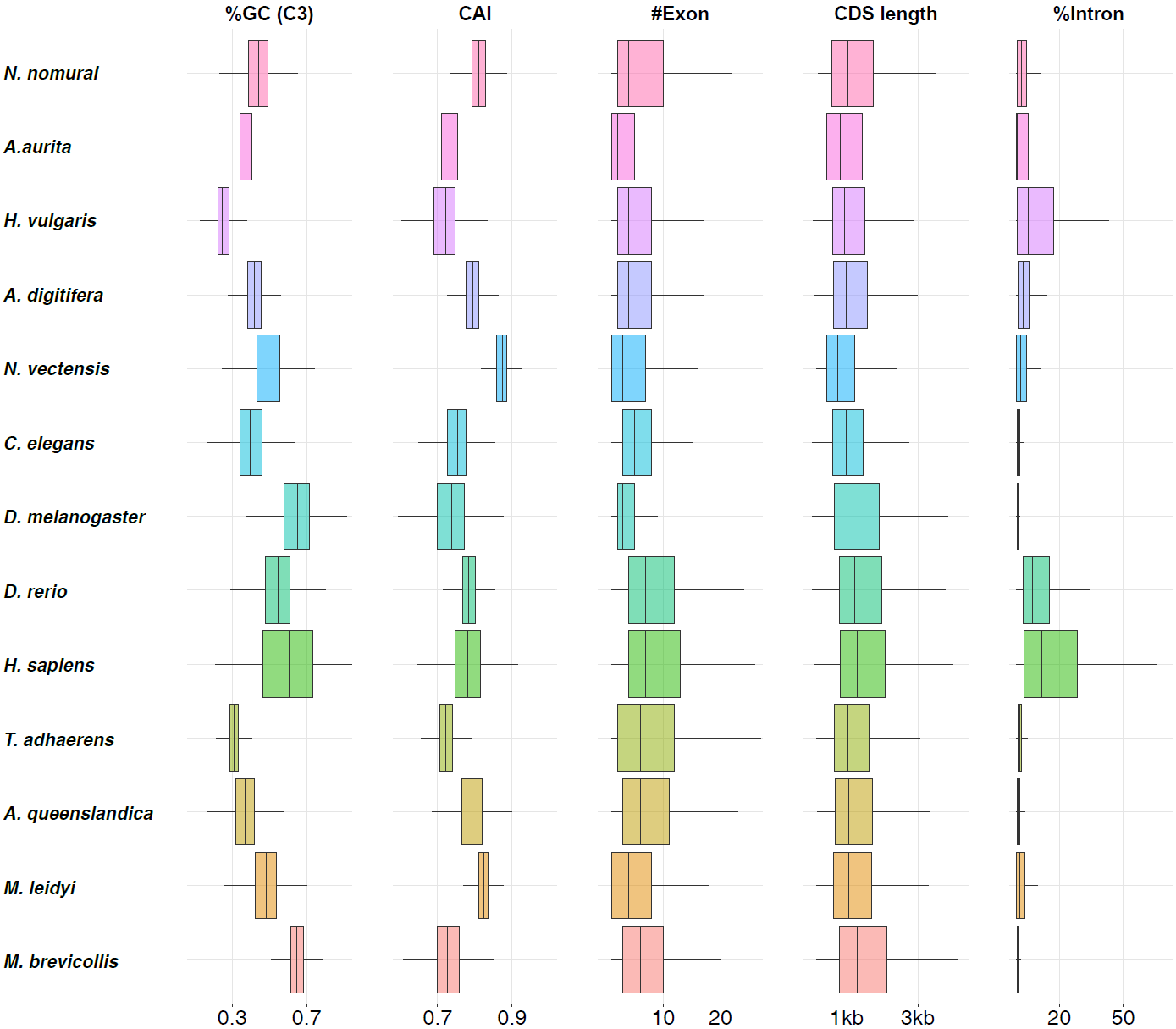


**Figure S13: Genomic context among twelve species.** Box plot distribution of genomic context corresponding to twelve species are shown. The %GC3 means the percentage of the guanine and cytosine content at the third codon position. The %intron means the length of all introns in CDS divided by CDS length. CAI: Codon Adaptation Index.

**5.2 Gene expression profiling of the jellyfish medusa bell and tentacles**

Illumina RNA libraries were prepared from the medusa bell and tentacles of one *Nemopilema nomurai* individual and were sequenced using a HiSeq2500 (Table S29). Reads were filtered out when the Q20 base content was lower than 70%, using the IlluQCPRLL.pl script from the NGSQCToolkit (version 2.3.3). The filtered reads were mapped to the *Nemopilema* genome assembly using TopHat. To avoid mapping ambiguity, only one unique hit (--max-multihits 1) was permitted. Gene expression of each sample was calculated using the Fragments Per Kilobase of transcript per Million mapped reads (FPKM) method in the program Cufflinks (v2.2.1) [58] and the resulting expression values were normalized using the upper quartile normalization method [94]. Differentially expressed genes (DEGs) between the tissues were identified by DEGseq [59]. Functional enrichment tests of the DEGs were conducted using the ClueGO application, with GO term (date 02.11.2016) and KEGG pathway (date 08.11.2016). Only terms with Bonferroni corrected *P*-value ≤ 0.01 were considered significant (Additional file 5: Tables S30-S37). The transcriptome sequencing and analysis of the *S. malayensis* medusa bell and tentacles are presented in Additional file 1: Section 7.1.

**Table S29: Transcriptome sequence statistics of the *Nemopilema nomurai* and *Sanderia malayensis* jellyfish.**

| **Species** | **Stage** | **Tissue** | **Number of  raw read pairs** | **Read  length (bp)** | **Total  bases (bp)** | **Number of clean  reads pairs** | **% of  clean reads** |
| --- | --- | --- | --- | --- | --- | --- | --- |
| *Nemopilema nomurai* | Medusa | Tentacles | 30,909,026 | 100 | 6,181,805,200 | 29,262,691 | 94.7% |
|  |  | Bell | 33,570,784 | 100 | 6,714,156,800 | 31,656,737 | 94.3% |
| *Sanderia malayensis* | Polyp | Mixed | 32,077,844 | 100 | 6,415,568,800 | 30,319,459 | 94.5% |
|  | Strobila | Mixed | 35,098,977 | 101 | 7,089,993,354 | 33,370,656 | 95.1% |
|  | Ephyra | Mixed | 34,706,479 | 100 | 6,941,295,800 | 32,956,730 | 95.0% |
|  | Medusa | Tentacles | 31,171,089 | 101 | 6,296,559,978 | 30,161,518 | 96.8% |
|  |  | Bell | 34,319,587 | 101 | 6,932,556,574 | 32,910,816 | 95.9% |
|  |  | Oral arms | 39,318,413 | 101 | 7,942,319,426 | 38,207,052 | 97.2% |

**5.3 Expansion of myosin heavy chain and light chain genes**

Myosins comprise a superfamily of motor proteins and play a critical role in muscle contraction and are involved in a wide range of motility processes in Eukaryotes. Critically, the Myosin II family proteins, found in cells of both striated muscle tissue and smooth muscle tissue, are responsible for producing contraction in muscle cells. Cnidarians possess both epitheliomuscular cells and striated muscle cells. Striated muscle is a critical component of the subumbrella of the medusa bell, where its fast contractions power the unique propulsion-based swimming of the jellyfish.

To compare motility-related genes, such as the Myosin II gene family, we aligned protein sequences of eight species to the well characterized human genes. We used two criteria for downstream analysis as follows:

1) BLAST reciprocal best hit to the human Myosin heavy chain and Myosin light chain genes.

2) BLAST best hit to the human Myosin heavy chain and Myosin light chain genes.

We adapted the BLAST reciprocal best hit method to identify the existence of a target gene, and the BLAST best hit method was used to identify additional gene copies that related to the Myosin heavy chain and Myosin light chain genes (Tables S38–S40). Using the BLAST reciprocal best hit method we found two Myosin heavy chain genes and five Myosin light chain genes in *Nemopilema,* the largest number of Myosin heavy and light chain genes in basal metazoans (Figures S14 and S15). Using the BLAST best hit method, *Nemopilema* were also shown to possess the most Myosin heavy and light related genes in basal metazoans (Figure S16). The domain structure of the MYH genes of *Nemopilema* is shown in Figure S17.

**Table S38: Number of reciprocal best hit and standard best hit genes.**

| Species | # of filtered protein coding genes | Reciprocal best  hit genes | Best hit  genes |
| --- | --- | --- | --- |
| *N. nomurai* | 18,962 | 6,499 | 11,890 |
| *A. aurita* | 24,818 | 5,289 | 13,119 |
| *H. vulgaris* | 17,331 | 5,329 | 11,457 |
| *N. vectensis* | 24,567 | 6,910 | 17,573 |
| *A. digitifera* | 25,295 | 6,506 | 16,616 |
| *T. adhaerens* | 11,491 | 5,799 | 9,257 |
| *A. queenslandica* | 12,811 | 5,454 | 10,731 |
| *M. leidyi* | 15,922 | 4,802 | 8,853 |
| *M. brevicollis* | 9,153 | 3,800 | 5,743 |

**Table S39: Copy number of Myosin heavy chain and Myosin light chain genes.** The numbers in each column indicate the number of similar gene copies based on standard blast best hit search. Numbers in parentheses mean high confidence gene copies that protein using a reciprocal best hit search strategy.

| **Phylum** | **Species** | **Number of Myosin heavy**  **chain genes** | **Number of Myosin light**  **chain genes** |
| --- | --- | --- | --- |
| Cnidaria | *N. nomurai* | 7 (2) | 21 (5) |
|  | *A. aurita* | 9 (2) | 19 (5) |
|  | *N. vectensis* | 5 (1) | 18 (4) |
|  | *A. digitifera* | 4 (2) | 22 (4) |
|  | *H. vulgaris* | 3 (1) | 11 (4) |
| Placozoa | *T. adhaerens* | 4 (1) | 4 (3) |
| Porifera | *A. queenslandica* | 2 (2) | 5 (3) |
| Ctenophora | *M. leidyi* | 5 (2) | 6 (1) |
| Holozoa | *M. brevicollis* | 3 (1) | 4 (2) |
| Bilateria | *H. sapiens* | 14 | 18 |

**Table S40: Structural and regulatory muscle proteins in cnidarians.** The gene symbols in each column are human gene symbols that satisfy BLAST reciprocal best hits with structural and regulatory muscle genes from human.

| **Catetory** | **Protein name** | ***Acropora*** | ***Nematostella*** | ***Nemopilema*** | ***Aurelia*** | ***Hydra*** |
| --- | --- | --- | --- | --- | --- | --- |
| Integrin-binding costamere proteins | melusin/chord/integrin-β1  binding protein | - | - | - | - | - |
|  | Talin | *TLN1* | *TLN2* | *TLN2* | *TLN2* | *TLN2* |
|  | Vinculin | *VCL* | *VCL* | *VCL* | *VCL* | *VCL** |
| Motor and regulatory proteins | Caldesmon | - | - | - | - | - |
|  | Calmodulin | *CALM3* | *CALM3* | *CALM3* | *CALM1* | *CALM3* |
|  | Calponin | *CNN3* | - | *CNN3* | CNN3 | - |
|  | Myosin essential light chain | - | - | *MYL4* | - | - |
|  | Myosin heavy chain type II | *MYH7\|MYH10* | *MYH6* | *MYH7\|MYH10* | *MYH6\|MYH10* | *MYH9* |
|  | Myosin regulatory light chain | *MYLIP* | *MYLIP* | *MYL9\|MYLIP* | *MYL9\|MYLIP* | *MYLIP* |
|  | Tropomyosin | *TPM1* | *TPM3* | *TPM3* | *TPM1* | *TPM3* |
|  | Titin | - | - | - | - | - |
|  | Troponin C | - | - | - | - | - |
|  | Troponin I | - | - | - | - | - |
|  | Troponin T | - | - | - | - | - |
| Dystroglycan-associated costamere proteins | α/β-Dystrobrevin | *DTNB* | *DTNB* | *DTNB* | *DTNB* | - |
|  | α/β-Dystroglycan | *DAG1* | *DAG1* | *DAG1* | *DAG1* | *DAG1* |
|  | α/ε-Sarcoglycan | *SGCE* | *SGCE* | *SGCE* | *SGCE*** | - |
|  | β-Sarcoglycan | *SGCB* | *SGCB* | *SGCB* | *SGCB** | *SGCB* |
|  | γ-syntrophin | *SNTG1* | *SNTG1* | - | - | - |
|  | Dystrophin | *DMD* | *DMD* | *DMD* | *DMD* | *DMD* |
|  | δ/γ/ζ-Sarcoglycan | *SGCG* | *SGCZ* | *SGCZ* | *SGCZ* | *SGCD* |
|  | α/β-syntrophin | *SNTB2* | *SNTB2* | *SNTB1* | *SNTB1* | *SNTB1* |

* This gene is present in the gene-set but was not a BLAST reciprocal best hit.

** This gene is absent in the gene-set but is present in the genome through TblastN search.


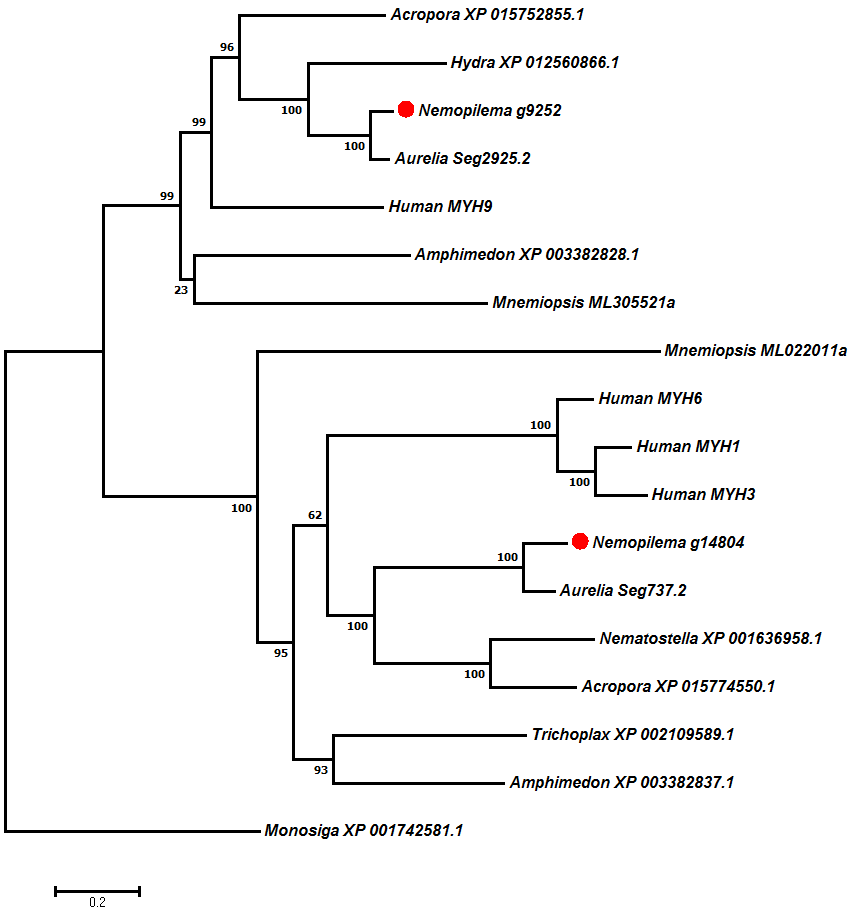


**Figure S14: Phylogenetic tree of Myosin heavy chain genes using the BLAST reciprocal best hit method.** The scale bar indicates the branch lengths measured in the number of amino acid substitutions per site.


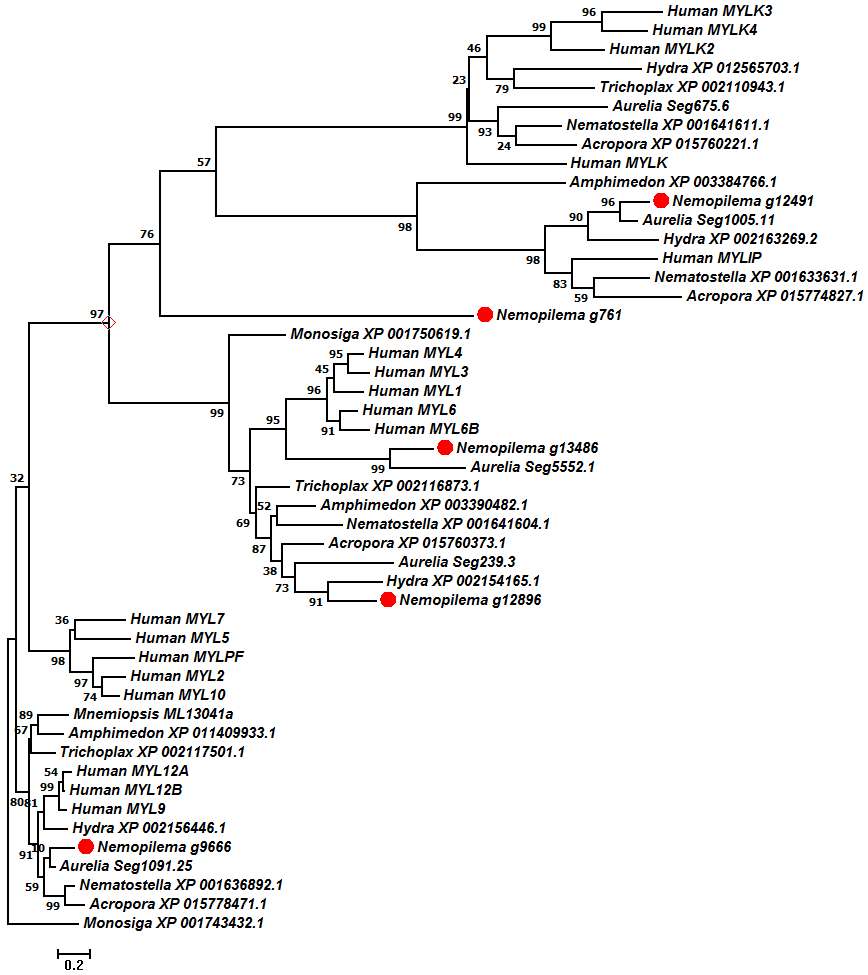


**Figure S15: Phylogenetic tree of Myosin light chain genes using the reciprocal best hit method.** The scale bar indicates the branch lengths measured in the number of amino acid substitutions per site.


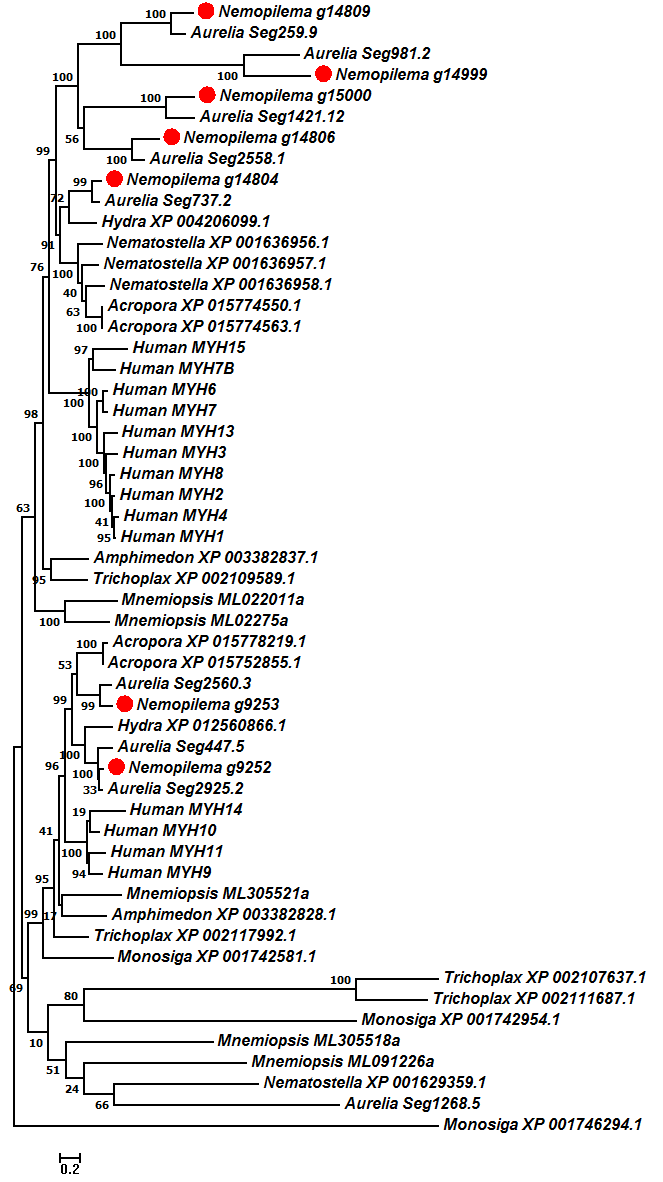


**Figure S16: Phylogenetic tree of Myosin heavy chain genes from the BLAST best hit method.** The scale bar indicates the branch lengths measured in the number of amino acid substitutions per site.


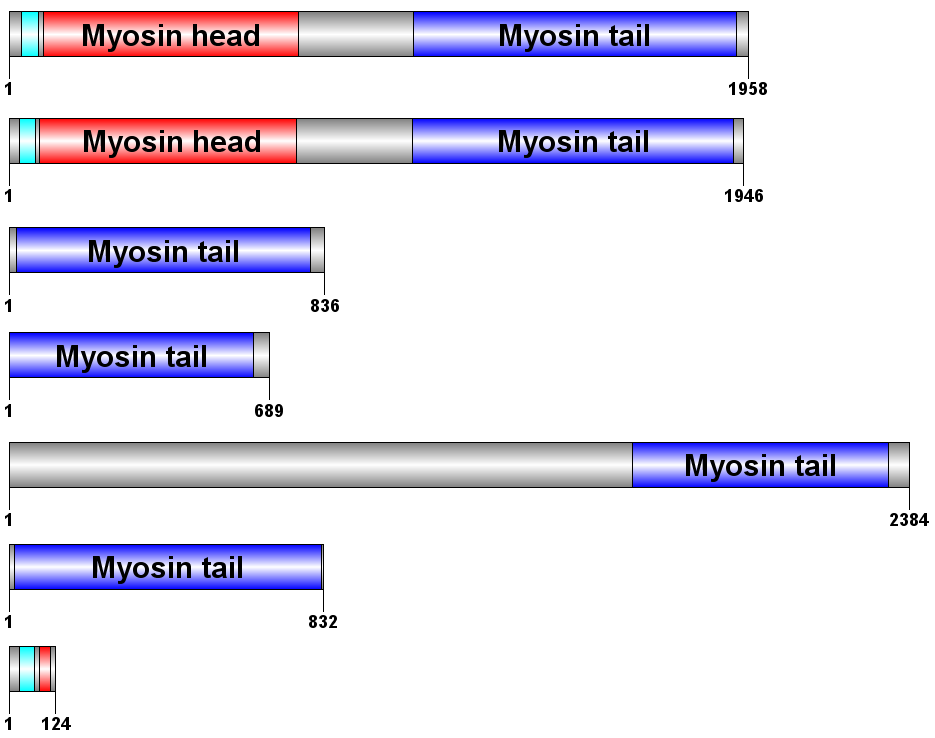


**Figure S17: Schematic representation of the Pfam domain structure of Myosin heavy chain genes using the BLAST best hit method.** Cyan denotes Myosin N-terminal SH3-like domain, red denotes Myosin head domain, and blue denotes Myosin tail domain.**6 Evolution of homeobox domain and *Wnt* gene subfamilies in cnidarians**

**6.1. Homeodomain evolution**

A homeobox is a DNA sequence, found within genes that are involved in the regulation of patterns of anatomical development (morphogenesis) in animals, fungi and plants [95]. These sequences encode a homeodomain protein that consists of 60-amino acid helix-turn-helix structure, which is highly conserved among animals. To analyze homeodomain evolution, cnidarian homeodomains were extracted from Pfam domain annotations. Homeodomains from ParaHox, Hox, and Hox related genes of human and fruit fly were used to investigate cnidarians homeodomain evolution using BLASTP best hits. We constructed multiple sequence alignments using MUSCLE and ran FastTree to generate phylogenies using the Jones–Taylor–Thornton (JTT) model. In total, 83 homeodomains were found in *Nemopilema*, while 41, 120, and 148 were found in *Hydra*, *Acropora*, and *Nematostella*, respectively (Table S41). Interestingly, the sum number of homeodomains of *Nemopilema*, *Acropora*, and *Nematostella* possess are about two, three, and four times more than those of *Hydra*. The most parsimonious explanation is that the common ancestor of Cnidaria had a similar number of homeodomains as *Acropora* and *Nematostella* [96, 97]. Thus, *Nemopilema* and *Hydra* have reduced numbers of homeodomains compared to those of the Cnidarian ancestor.

**Table S41: Presence of Hox, Hox-related, and ParaHox homeobox domains in Cnidaria.**

| Category | Genes | Species | | | | |
| --- | --- | --- | --- | --- | --- | --- |
|  |  | *Nemopilema* | *Aurelia* | *Hydra* | *Acropora* | *Nematostella* |
| Hox-related | *EVX* | O | O | - | O | O |
|  | *EMX* | O | O | - | O | O |
|  | *MOX* | O | O | O | O | O |
|  | *GBX* | - | - | - | O | O |
|  | *MNX* | - | - | - | O | O |
|  | *DLX* | O | O | O | O | O |
|  | *MSX* | O | O | O | O | O |
| ParaHox | *GSX* | O | O | O | O | O |
|  | *XLOX*/*CDX* (*PDX*) | O | O | - | - | O |
| Number of Hox genes | | 8 | 7 | 6 | 6 | 7 |
| Total number of  homeobox domain | | 83 | 82 | 41 | 120 | 148 |

**6.2 ParaHox, Hox, and Hox related genes in cnidarians**

Hox genes are a subset of homeobox genes and are essential metazoan genes that determine the identity of embryonic regions along the anterior-posterior axis [98]. We searched Hox genes in cnidarian genomes using the Hox gene homeodomains of human and fruit fly (Figure S18). Eight Hox genes were found in *Nemopilema* that are in similar clusters as other cnidarians. *HOX3* and central Hox genes were missing in *Nemopilema* as other cnidarians; however, interestingly, five of the eight Hox genes in *Nemopilema* are posterior Hox genes (Figures S19 and S20). This supports previous reports of the absence of central Hox genes within Cnidaria, providing further evidence that these genes arose in the bilateral metazoans [23, 24, 60]. The five *Nemopilema* posterior Hox genes were clustered with *Nematostella’s* posterior Hox genes, *HOXE* and *HOXF*. Other cnidarian species, *Nematostella* (Anthozoa) [23, 24, 60], *Clytia hemisphaerica* (Hydrozoa) [61], *Cassiopeia xamachana* (Scyphozoa) [99], and *Hydra* [23] have two, three, four and five copies of posterior Hox genes, respectively. Posterior Hox genes are related to the development of aboral pole, and *Nemopilema* and *Hydra* have the largest number of posterior Hox genes among Cnidaria. ParaHox genes, *GSX,* and a gene related to *XLOX* and *CDX* are also found in *Nemopilema*, though *Hydra* and *Acropora* possess only the *GSX* gene (see Table S41). These two *Nemopilema* ParaHox genes are phylogenetically clustered with other ParaHox genes in Cnidaria and Bilateria. Synteny analyses show that the candidate *XLOX/CDX* gene is located immediately downstream of *GSX*, in the same tandem orientation as those in *Nematostella* (Figure S21). Hox related genes, *EVX* and *EMX,* are also present in *Nemopilema*, although they are absent in *Hydra*. Importantly, together these results suggest that *XLOX/CDX, EVX*, and *EMX* were present in the cnidarian common ancestor, and were subsequently lost in some cnidarian lineages.


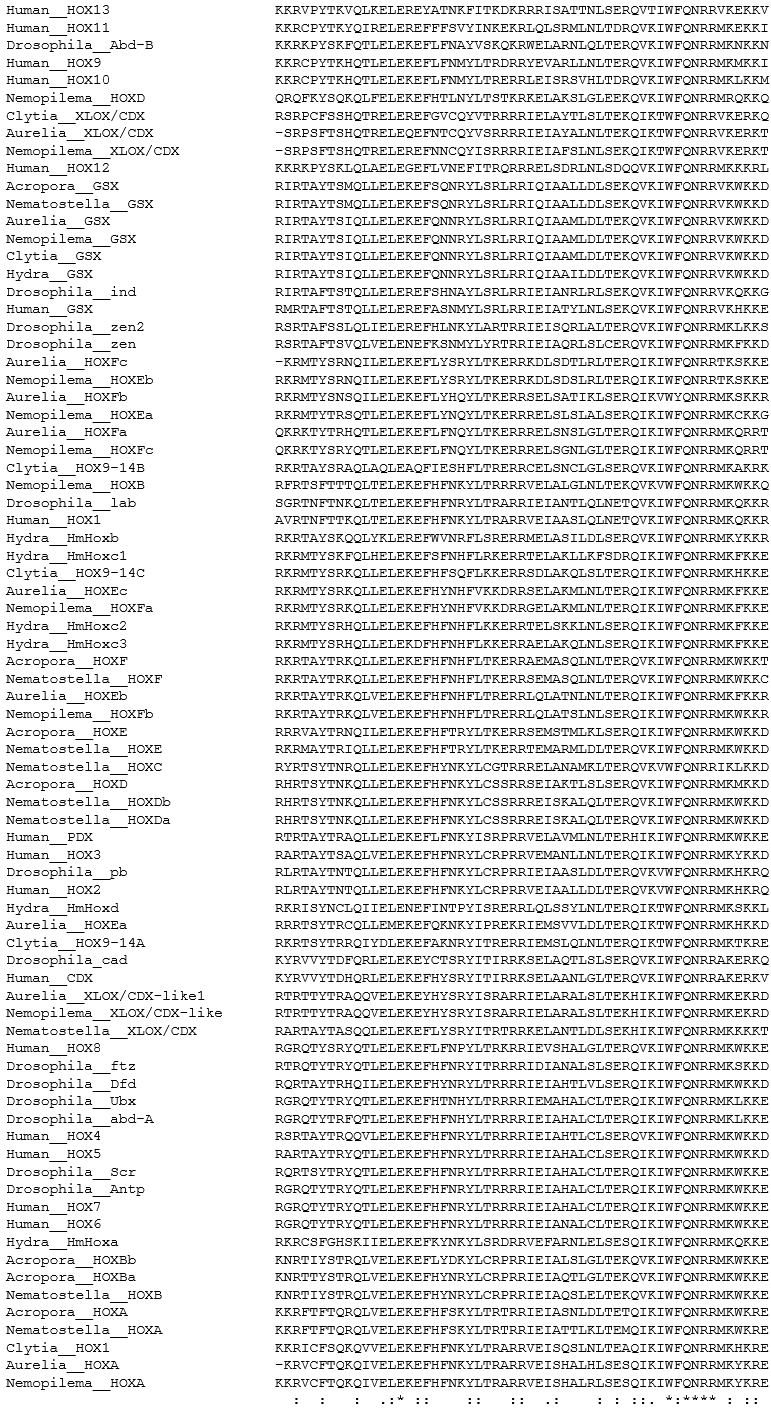


**Figure S18: Multiple sequence alignment of homeodomains for Hox and ParaHox genes with human, fruit fly, and cnidarians.**

**
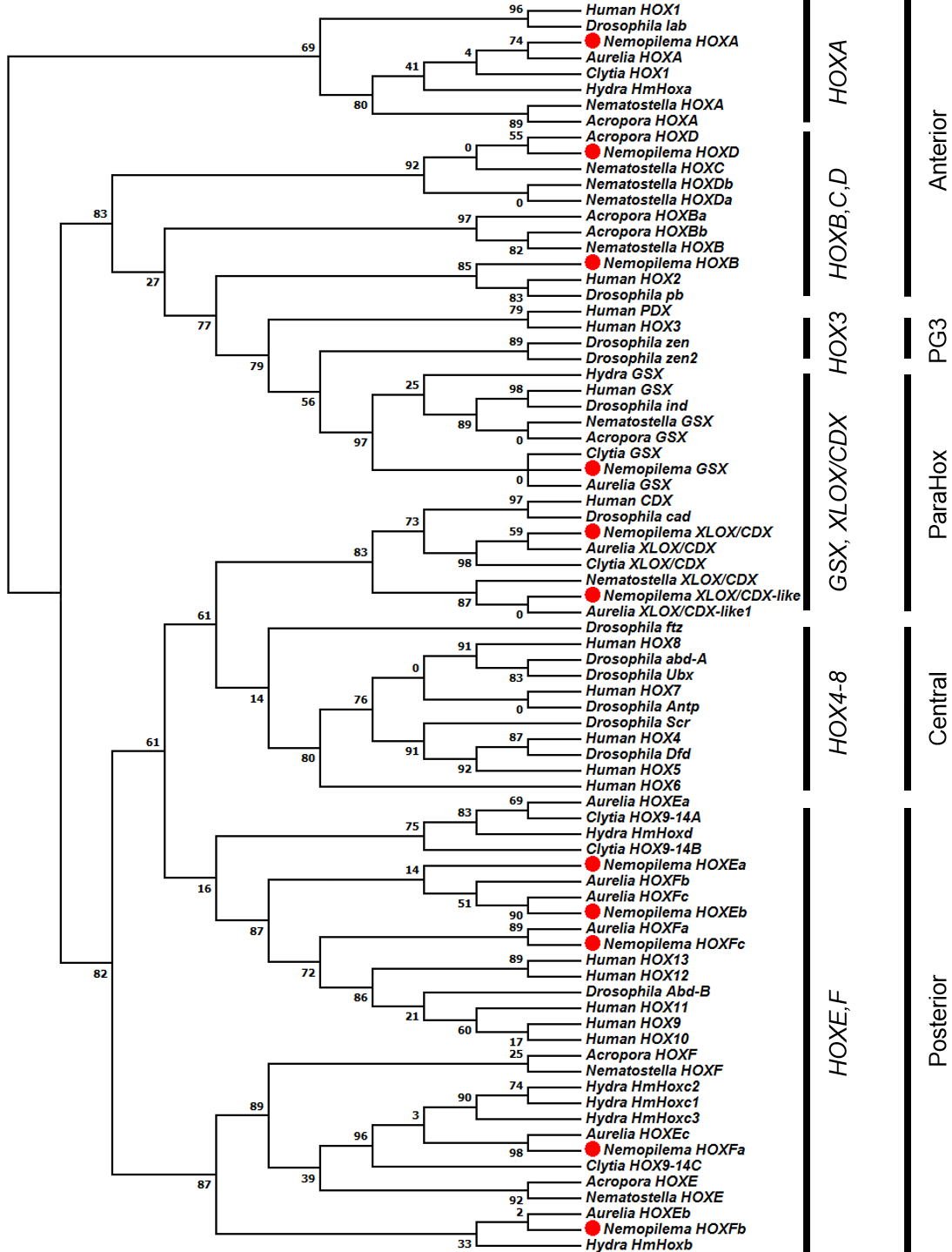
**

**Figure S19: Phylogenetic analysis of Hox and ParaHox homeodomains with human, fruit fly, and cnidarians.** Numbers on nodes denote bootstrap values based on 100 iterations.


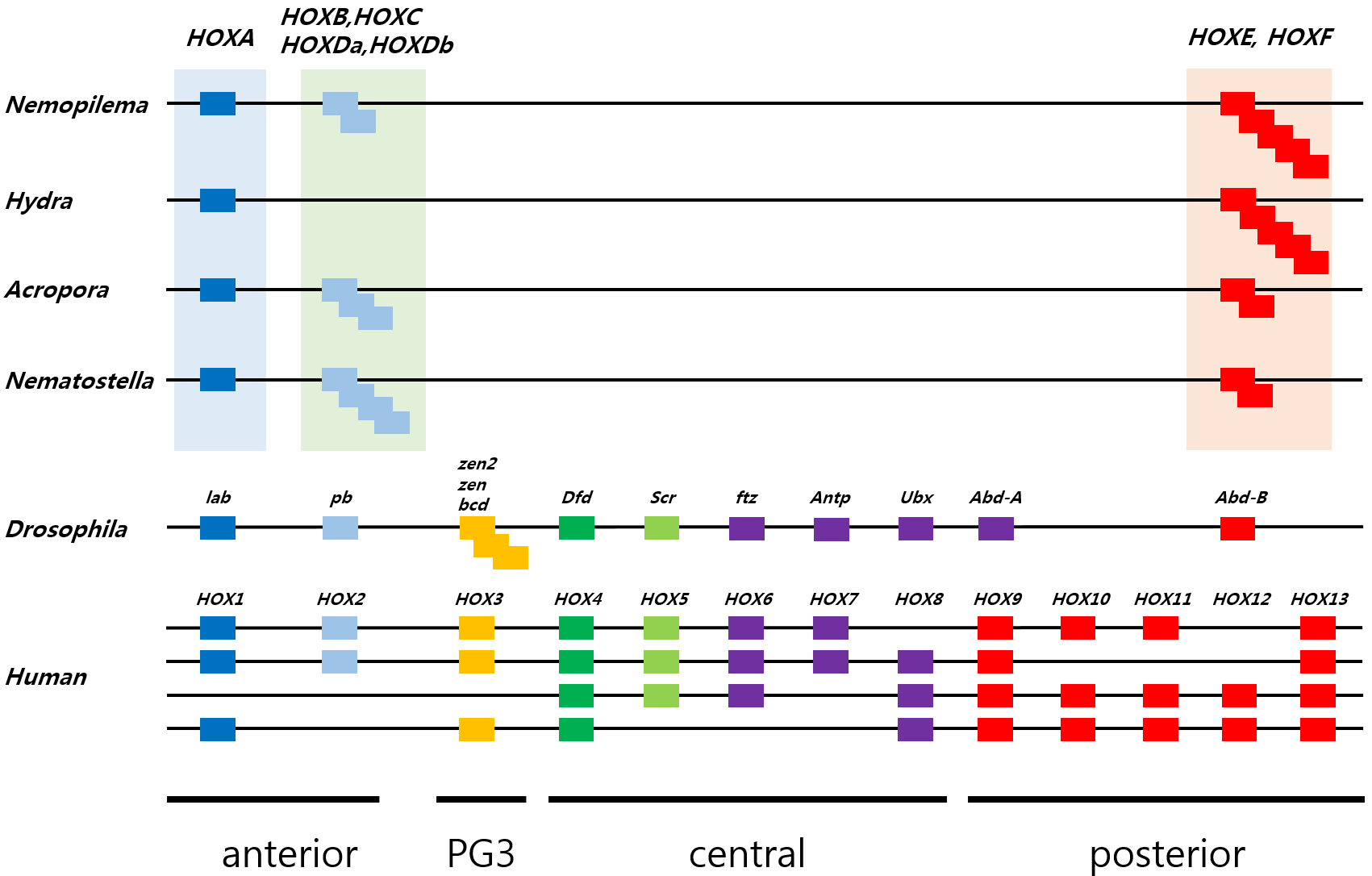


**Figure S20: Presence and absence of Hox genes in cnidarians.** Blue denotes anterior Hox genes, yellow denotes paralogue group 3 (PG3) Hox genes, green and purple denote central Hox genes and red denotes posterior Hox genes.


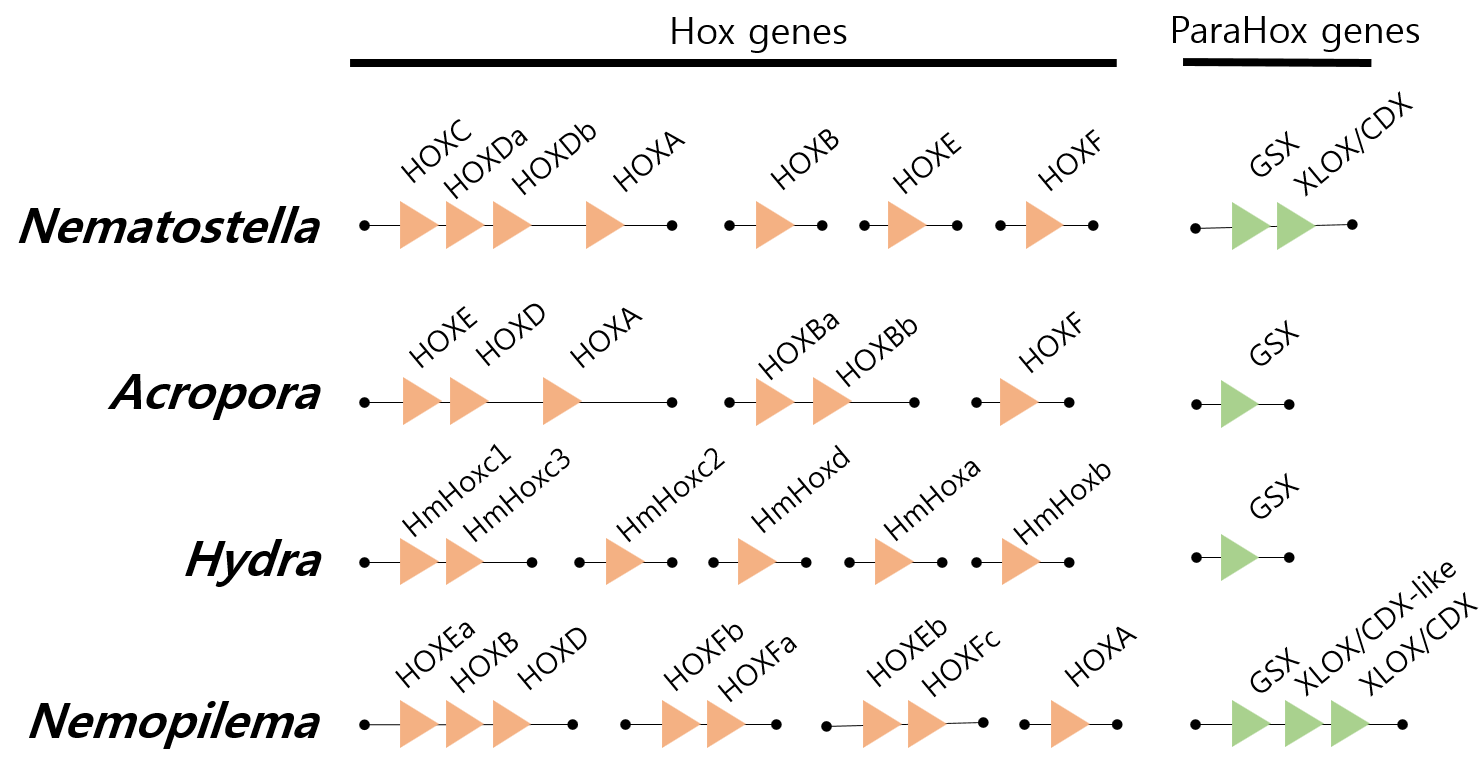


**Figure S21: Arrangements of Hox and ParaHox genes in cnidarians.** Orange denotes Hox genes, and green denotes ParaHox genes.

**6.3 *Wnt* gene subfamilies in cnidarians**

The Wnt signal transduction pathway is an evolutionarily conserved signaling pathway that regulates many aspects of metazoan development [100], and has been proposed to have controlled body plan development in the early metazoans (Kusserow *et al.* 2005). The *Wnt* genes encode Wnt ligands which are involved pathways related to pattern formation, morphogenesis, and organogenesis. In Cnidaria, previous analyses of the *Wnt* genes have focused on *Hydra* and *Nematostella*, which have 11 and 13 *Wnt* genes, respectively [25, 63]. Analyses of *Wnt* genes in *Nemopilema* revealed clusters of ten of the thirteen known *Wnt* gene subfamilies (Table S42). *Wnt6* is present in *Acropora* and *Nematostella*, but appears to have been lost in the common ancestor of *Hydra* and *Nemopilema* (Figure S22)*.* Absent in *Hydra* and bilaterians, but present in *Nemopilema*, *WntA* is expressed in the early gastrula as a broad expression domain defining the site of gastrulation, and in *Nematostella* extends into the involuting ectodermal epithelial layer at late gastrula stages [25]. Data from several taxa indicate that cnidarians possess most *Wnt* gene families found in bilaterians, and in many cases expansion of the gene families occurred before the cnidarians diverged [25, 63].


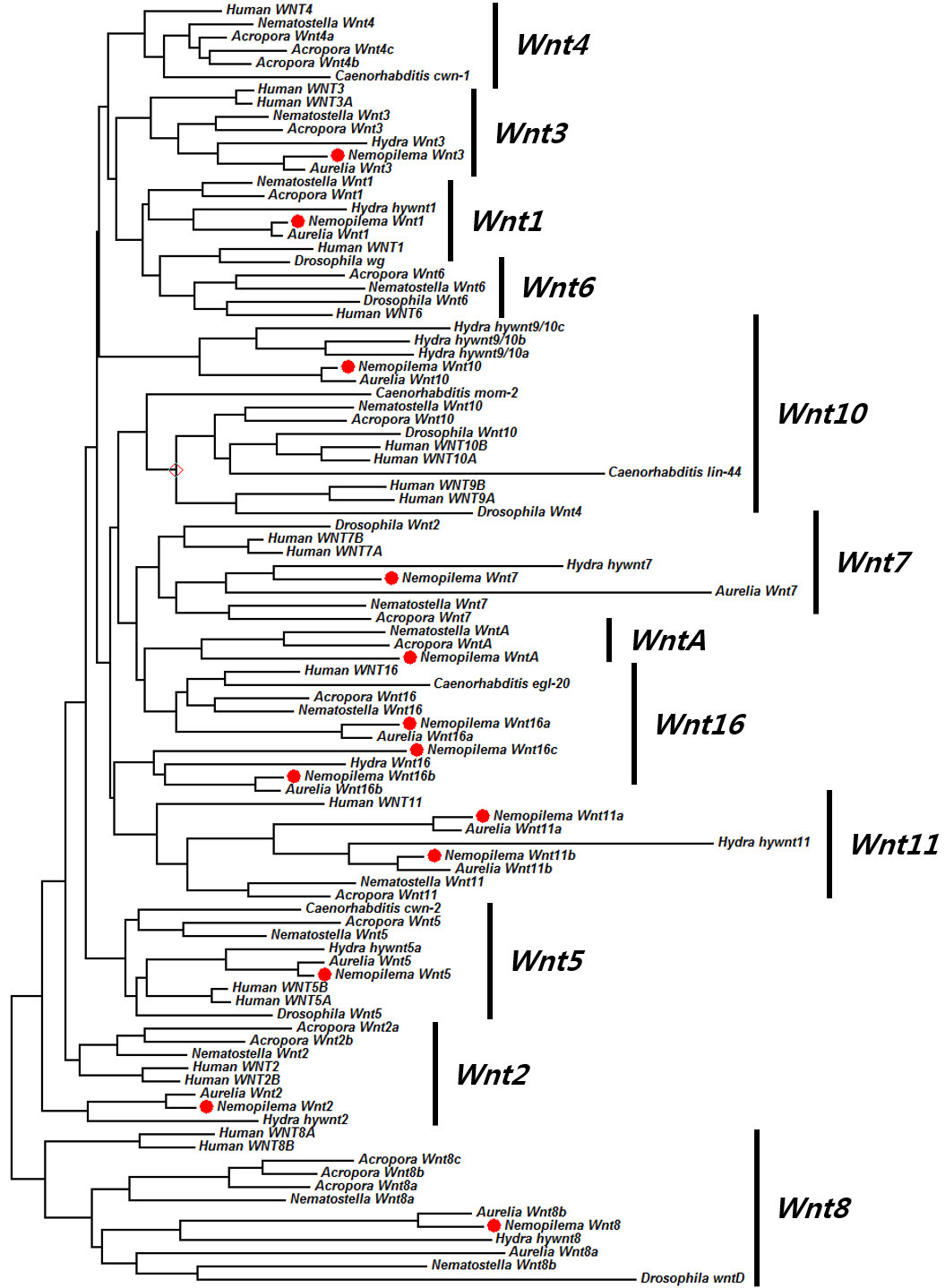


**Figure S22:** **Phylogenetic tree using Maximum likelihood of Wnt proteins.**


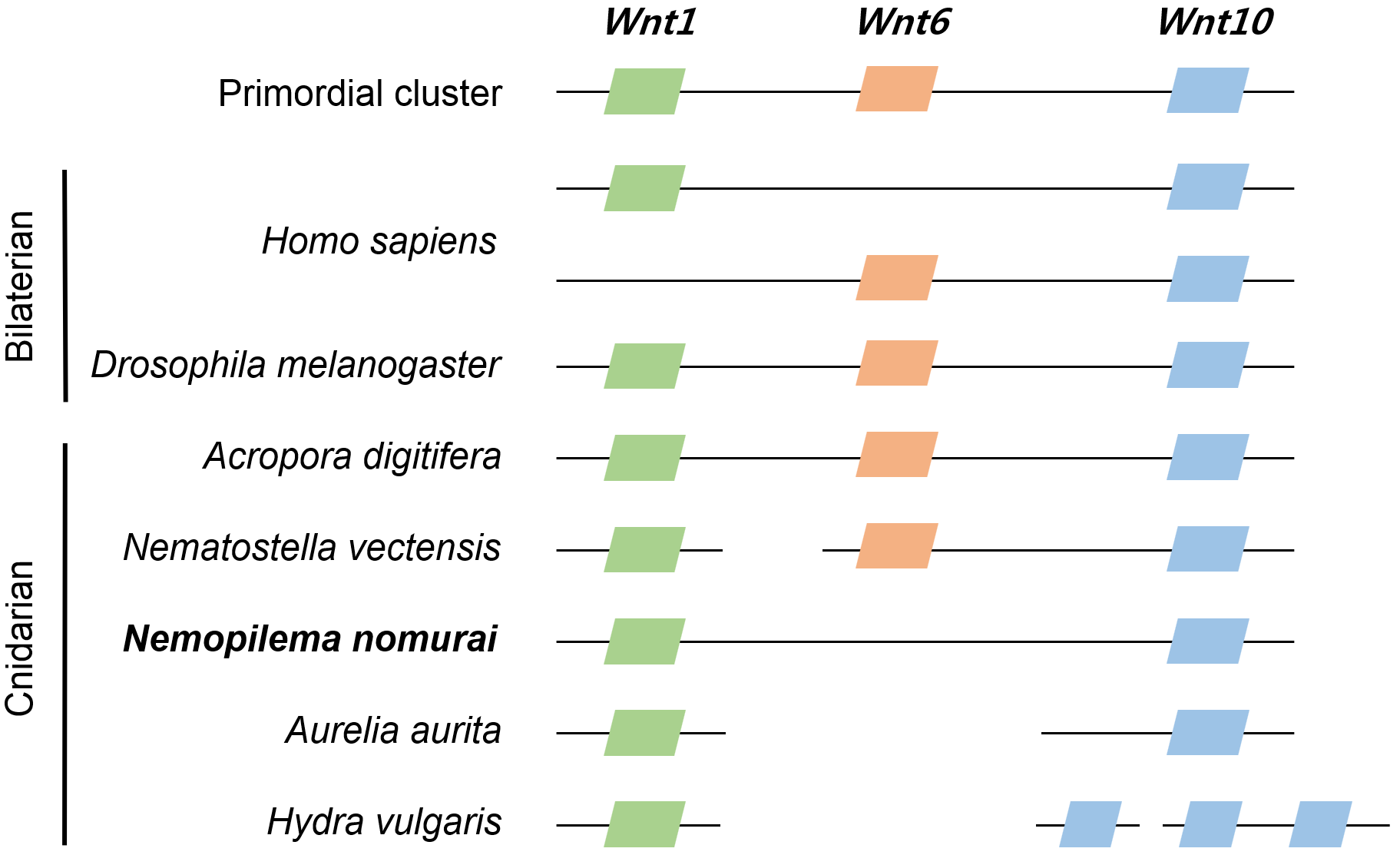


**Figure S23: A primordial cluster of three *Wnt* gene (*Wnt1*–*Wnt6*–*Wnt10*) pattern of cnidarians.**

**Table S42: Distribution of *Wnt* genes among cnidarians.**

|  | Cnidaria | | | | | Arthropoda | Chordata |
| --- | --- | --- | --- | --- | --- | --- | --- |
|  | Scyphozoa | | Hydrozoa | Anthozoa | | Insecta | Mammalia |
| **Gene** | ***Nemopilema*** | ***Aurelia*** | ***Hydra*** | ***Nematostella*** | ***Acropora*** | ***Drosophila*** | **Human** |
| *Wnt1* | 1 | 1 | 1 | 1 | 1 | 1 | 1 |
| *Wnt2* | 1 | 1 | 1 | 1 | 2 | 0 | 2 |
| *Wnt3* | 1 | 1 | 1 | 1 | 1 | 0 | 2 |
| *Wnt4* | 0 | 0 | 0 | 1 | 3 | 0 | 1 |
| *Wnt5* | 1 | 1 | 1 | 1 | 1 | 1 | 2 |
| *Wnt6* | 0 | 0 | 0 | 1 | 1 | 1 | 1 |
| *Wnt7* | 1 | 1 | 1 | 1 | 1 | 1 | 2 |
| *Wnt8* | 1 | 2 | 1 | 2 | 3 | 0 | 2 |
| *Wnt9* | 0 | 0 | 0 | 0 | 0 | 1 | 2 |
| *Wnt10* | 1 | 1 | 3 | 1 | 1 | 1 | 2 |
| *Wnt11* | 2 | 2 | 1 | 1 | 1 | 0 | 1 |
| *Wnt16* | 3 | 2 | 1 | 1 | 1 | 0 | 1 |
| *WntA* | 1 | 0 | 0 | 1 | 1 | 0 | 0 |
| Other | 0 | 0 | 0 | 0 | 0 | 1 | 0 |
| **Total** | **13** | **12** | **11** | **13** | **17** | **7** | **19** |

**7 Jellyfish developmental stage and retinoic acid signaling**

**7.1 RNA sequencing and assembly**

To understand the developmental basis of molecular function in jellyfish, we performed transcriptome profiling of the four developmental stages (polyp, strobila, ephyra, and three medusa stage tissues of bell, tentacles, and oral arms) of *Sanderia malayensis*. Because of the large body size of *Nemopilema*, it is difficult to raise in the laboratory. Therefore, we conducted transcriptome experiments using *S. malayensis*, which belongs to the same class Scyphozoa and provides an additional species for verification of our evolutionary findings and allows us to extend our inferences more broadly to jellyfish. *S. malayensis* Illumina RNA libraries were sequenced using HiSeq2500 with 100bp and 101bp read lengths (see Table S29). Reads were filtered out when the Q20 base content was lower than 70%, using the IlluQCPRLL.pl script in the NGSQCToolkit. All downstream transcriptome analyses were based on this filter passed data.

*De novo* transcriptome assembly was conducted using Trinity v2.2.0 with default options [57]. The filtered RNA reads from five *S. malayensis* transcriptome samples were used for assembly (Table S43). Assembled transcripts had a total length of 61 Mb and resulted in 58,290 transcript isoforms and 43,541 unique transcripts, with a N50 of 2,325 bp. Transcripts annotated to non-metazoan or *Artemia* (the captive jellyfish food source) were considered as possible contamination and filtered out. The filtered transcripts were translated by TransDecoder program [101], and we obtained 27,915 peptide sequences that were used in downstream analysis. The peptide sequences were annotated by InterProScan-5.13.52.0 program with Pfam database. To calculate the expression value for each stage, the filtered RNA reads from each sample were mapped to the filtered transcriptome assembly using TopHat with the “--max-multihits 1” option. To assess the quality of the transcript assembly, we aligned the input reads to the assembly. On average, 87% of the reads were aligned to into the assembled transcripts (Table S44), indicating that the transcript assembly represented the majority of sequenced reads. Furthermore, the composition of the protein domains contained in the top 20 ranks was quite similar between *Nemopilema* and *Sanderia* (Table S45)**.**

**Table S43: Transcriptome assembly statistics of *S. malayensis.***

|  | Raw transcript  assembly | Redundant filtered  transcripts assembly |
| --- | --- | --- |
| Total number of transcripts | 58,290 | 43,541 |
| Total assembled bases | 61,042,797 bp | 41,667,470 bp |
| Average length | 1,047.23 bp | 956.97 bp |
| Longest length | 25,200 bp | 25,200 bp |
| Shortest length | 201 bp | 201 bp |
| N50 | 2,399 bp | 2,325 bp |
| GC contents | 39.94% | 39.74% |

**Table S44: Quality assessment of *S. malayensis* transcript assembly using self-alignment.**

| **Stage** | **Tissue** | **Number of clean**  **read pairs** | **Mapping rate  (left / right read)** | **% of concordant pairs** |
| --- | --- | --- | --- | --- |
| Polyp | Mixed | 30,319,459 | 25,850,143 / 26,428,010 (85.3/87.2%) | 86.2% |
| Strobila | Mixed | 33,370,656 | 29,819,262 / 28,675,253 (89.4/85.9%) | 87.6% |
| Ephyra | Mixed | 32,956,730 | 28,474,944 / 29,141,521 (86.4/88.4%) | 87.4% |
| Medusa | Tentacles | 30,161,518 | 26,257,062 / 26,322,083 (87.1/87.3%) | 87.2% |
|  | Bell | 32,910,816 | 29,727,094 / 29,794,976 (90.3/90.5%) | 90.4% |
|  | Oral arms | 38,207,052 | 33,535,658 / 33,653,438 (87.8/88.1%) | 87.9% |

**Table S45: The most abundant protein domains in *Nemopilema* and *Sanderia*.** Only the top 20 abundant domains are shown.

|  | *Nemopilema* (from Gene set) | | | *Sanderia* (from Transcriptome) | | |
| --- | --- | --- | --- | --- | --- | --- |
| Rank | Pfam ID | Pfam domain name | Domain  count | Pfam ID | Pfam domain name | Domain  count |
| 1 | PF00400 | WD domain, G-beta repeat | 839 | PF00400 | WD domain, G-beta repeat | 855 |
| 2 | PF00001 | 7 transmembrane receptor  (rhodopsin family) | 422 | PF00001 | 7 transmembrane receptor  (rhodopsin family) | 386 |
| 3 | PF12796 | Ankyrin repeats (3 copies) | 353 | PF00008 | EGF-like domain | 328 |
| 4 | PF00008 | EGF-like domain | 293 | PF12796 | Ankyrin repeats (3 copies) | 316 |
| 5 | PF13465 | Zinc-finger double domain | 280 | PF07679 | Immunoglobulin I-set domain | 246 |
| 6 | PF00078 | Reverse transcriptase  (RNA-dependent DNA polymerase) | 280 | PF07645 | Calcium-binding EGF domain | 234 |
| 7 | PF07679 | Immunoglobulin I-set domain | 277 | PF00028 | Cadherin domain | 234 |
| 8 | PF00041 | Fibronectin type III domain | 257 | PF13465 | Zinc-finger double domain | 226 |
| 9 | PF00028 | Cadherin domain | 233 | PF00090 | Thrombospondin type 1 domain | 225 |
| 10 | PF00069 | Protein kinase domain | 231 | PF00069 | Protein kinase domain | 218 |
| 11 | PF07645 | Calcium-binding EGF domain | 221 | PF01549 | ShK domain-like | 213 |
| 12 | PF00090 | Thrombospondin type 1 domain | 215 | PF00041 | Fibronectin type III domain | 205 |
| 13 | PF00431 | CUB domain | 187 | PF01391 | Collagen triple helix repeat (20 copies) | 196 |
| 14 | PF00665 | Integrase core domain | 178 | PF00076 | RNA recognition motif.  (a.k.a. RRM, RBD, or RNP domain) | 188 |
| 15 | PF01391 | Collagen triple helix repeat (20 copies) | 168 | PF00431 | CUB domain | 158 |
| 16 | PF00076 | RNA recognition motif.  (a.k.a. RRM, RBD, or RNP domain) | 167 | PF00053 | Laminin EGF-like (Domains III and V) | 145 |
| 17 | PF00053 | Laminin EGF-like (Domains III and V) | 153 | PF01344 | Kelch motif | 144 |
| 18 | PF13499 | EF-hand domain pair | 147 | PF00595 | PDZ domain  (Also known as DHR or GLGF) | 136 |
| 19 | PF01549 | ShK domain-like | 141 | PF00435 | Spectrin repeat | 134 |
| 20 | PF00092 | von Willebrand factor type A domain | 141 | PF13499 | EF-hand domain pair | 132 |

**7.2 Gene expression profiling and differentially expressed genes in developmental stages**

Gene expression values for each sample was calculated as described in Additional file 1: 5.2. To obtain differentially expressed genes for each stage, we compared each stage with the previous stage in the life cycle of the jellyfish. For example, polyp stage was compared with strobila stage, and strobila stage was compared with previous polyp stage and next ephyra stage. A gene ontology (GO) functional enrichment test was performed for each combination. Only shared GO terms for medusa tissues were considered as medusa-stage DEGs (Tables S46–S49).

**Table S46: GO enrichment of highly expressed genes in polyp compared to strobila stage.**

| GO Id | GO Term | *P*-value | FDR |
| --- | --- | --- | --- |
| GO:0099600 | transmembrane receptor activity | 5.38E-08 | 4.54E-05 |
| GO:0004872 | receptor activity | 7.64E-08 | 4.54E-05 |
| GO:0060089 | molecular transducer activity | 1.33E-07 | 5.27E-05 |
| GO:0015267 | channel activity | 5.59E-07 | 9.50E-05 |
| GO:0005216 | ion channel activity | 5.59E-07 | 9.50E-05 |
| GO:0022838 | substrate-specific channel activity | 5.59E-07 | 9.50E-05 |
| GO:0022803 | passive transmembrane transporter activity | 5.59E-07 | 9.50E-05 |
| GO:0016020 | membrane | 3.09E-06 | 4.59E-04 |
| GO:0005230 | extracellular ligand-gated ion channel activity | 1.76E-05 | 2.33E-03 |
| GO:0015276 | ligand-gated ion channel activity | 3.52E-05 | 3.80E-03 |
| GO:0022834 | ligand-gated channel activity | 3.52E-05 | 3.80E-03 |
| GO:0043565 | sequence-specific DNA binding | 4.19E-05 | 4.05E-03 |
| GO:0038023 | signaling receptor activity | 4.43E-05 | 4.05E-03 |
| GO:0003700 | DNA binding transcription factor activity | 5.15E-05 | 4.32E-03 |
| GO:0140110 | transcription regulator activity | 5.45E-05 | 4.32E-03 |
| GO:0004871 | signal transducer activity | 6.06E-05 | 4.44E-03 |
| GO:0022836 | gated channel activity | 6.35E-05 | 4.44E-03 |
| GO:0005509 | calcium ion binding | 8.04E-05 | 5.31E-03 |
| GO:0010468 | regulation of gene expression | 8.50E-05 | 5.32E-03 |
| GO:0004888 | transmembrane signaling receptor activity | 2.00E-04 | 1.19E-02 |
| GO:0016021 | integral component of membrane | 2.49E-04 | 1.35E-02 |
| GO:0031224 | intrinsic component of membrane | 2.49E-04 | 1.35E-02 |
| GO:0060255 | regulation of macromolecule metabolic process | 2.72E-04 | 1.41E-02 |
| GO:0015318 | inorganic molecular entity transmembrane transporter activity | 2.97E-04 | 1.47E-02 |
| GO:0050789 | regulation of biological process | 3.35E-04 | 1.59E-02 |
| GO:0019222 | regulation of metabolic process | 5.18E-04 | 2.37E-02 |
| GO:0004930 | G-protein coupled receptor activity | 6.64E-04 | 2.85E-02 |
| GO:2001141 | regulation of RNA biosynthetic process | 7.46E-04 | 2.85E-02 |
| GO:0006355 | regulation of transcription, DNA-templated | 7.46E-04 | 2.85E-02 |
| GO:1903506 | regulation of nucleic acid-templated transcription | 7.46E-04 | 2.85E-02 |
| GO:0051252 | regulation of RNA metabolic process | 7.46E-04 | 2.85E-02 |
| GO:0015075 | ion transmembrane transporter activity | 7.67E-04 | 2.85E-02 |
| GO:0022892 | substrate-specific transporter activity | 8.00E-04 | 2.88E-02 |
| GO:0006811 | ion transport | 8.28E-04 | 2.89E-02 |
| GO:0005215 | transporter activity | 8.95E-04 | 2.89E-02 |
| GO:0065007 | biological regulation | 9.68E-04 | 2.89E-02 |
| GO:0022857 | transmembrane transporter activity | 1.00E-03 | 2.89E-02 |
| GO:0010556 | regulation of macromolecule biosynthetic process | 1.01E-03 | 2.89E-02 |
| GO:2000112 | regulation of cellular macromolecule biosynthetic process | 1.01E-03 | 2.89E-02 |
| GO:0009889 | regulation of biosynthetic process | 1.01E-03 | 2.89E-02 |
| GO:0031326 | regulation of cellular biosynthetic process | 1.01E-03 | 2.89E-02 |
| GO:0019219 | regulation of nucleobase-containing compound metabolic process | 1.02E-03 | 2.89E-02 |
| GO:0022891 | substrate-specific transmembrane transporter activity | 1.08E-03 | 2.99E-02 |
| GO:0006022 | aminoglycan metabolic process | 1.16E-03 | 3.13E-02 |
| GO:1901071 | glucosamine-containing compound metabolic process | 1.29E-03 | 3.26E-02 |
| GO:0006030 | chitin metabolic process | 1.29E-03 | 3.26E-02 |
| GO:0006040 | amino sugar metabolic process | 1.29E-03 | 3.26E-02 |
| GO:0050794 | regulation of cellular process | 1.36E-03 | 3.37E-02 |
| GO:0008083 | growth factor activity | 1.94E-03 | 4.71E-02 |
| GO:0051171 | regulation of nitrogen compound metabolic process | 2.02E-03 | 4.80E-02 |

**Table S47: GO enrichment of highly expressed genes in strobila compared to polyp stage.**

| GO Id | GO Term | *P*-value | FDR |
| --- | --- | --- | --- |
| GO:0003735 | structural constituent of ribosome | 2.45E-24 | 2.16E-21 |
| GO:0005198 | structural molecule activity | 1.42E-23 | 6.26E-21 |
| GO:1901566 | organonitrogen compound biosynthetic process | 1.39E-22 | 4.08E-20 |
| GO:0005840 | ribosome | 6.37E-22 | 1.40E-19 |
| GO:0006412 | translation | 1.46E-21 | 2.14E-19 |
| GO:0043043 | peptide biosynthetic process | 1.46E-21 | 2.14E-19 |
| GO:0043604 | amide biosynthetic process | 6.86E-21 | 8.63E-19 |
| GO:0006518 | peptide metabolic process | 8.27E-21 | 9.11E-19 |
| GO:0043603 | cellular amide metabolic process | 8.38E-20 | 8.20E-18 |
| GO:0030529 | intracellular ribonucleoprotein complex | 2.46E-17 | 1.97E-15 |
| GO:1990904 | ribonucleoprotein complex | 2.46E-17 | 1.97E-15 |
| GO:0009058 | biosynthetic process | 4.62E-17 | 3.39E-15 |
| GO:0044271 | cellular nitrogen compound biosynthetic process | 9.50E-17 | 6.44E-15 |
| GO:0044249 | cellular biosynthetic process | 3.64E-16 | 2.29E-14 |
| GO:0034645 | cellular macromolecule biosynthetic process | 9.06E-16 | 5.32E-14 |
| GO:1901576 | organic substance biosynthetic process | 1.57E-15 | 8.64E-14 |
| GO:0009059 | macromolecule biosynthetic process | 2.69E-14 | 1.39E-12 |
| GO:0043232 | intracellular non-membrane-bounded organelle | 1.07E-12 | 4.96E-11 |
| GO:0043228 | non-membrane-bounded organelle | 1.07E-12 | 4.96E-11 |
| GO:0044444 | cytoplasmic part | 8.36E-12 | 3.68E-10 |
| GO:0005622 | intracellular | 2.46E-08 | 1.03E-06 |
| GO:0032991 | macromolecular complex | 6.67E-06 | 2.67E-04 |
| GO:0034641 | cellular nitrogen compound metabolic process | 3.25E-05 | 1.24E-03 |
| GO:0098800 | inner mitochondrial membrane protein complex | 3.88E-05 | 1.37E-03 |
| GO:0098798 | mitochondrial protein complex | 3.88E-05 | 1.37E-03 |
| GO:1901564 | organonitrogen compound metabolic process | 5.05E-05 | 1.71E-03 |
| GO:0043565 | sequence-specific DNA binding | 1.02E-04 | 3.33E-03 |
| GO:0044455 | mitochondrial membrane part | 1.82E-04 | 5.73E-03 |
| GO:0044281 | small molecule metabolic process | 2.50E-04 | 7.52E-03 |
| GO:0055086 | nucleobase-containing small molecule metabolic process | 2.56E-04 | 7.52E-03 |
| GO:0045263 | proton-transporting ATP synthase complex, coupling factor F(o) | 3.32E-04 | 8.63E-03 |
| GO:0009199 | ribonucleoside triphosphate metabolic process | 4.21E-04 | 8.63E-03 |
| GO:0006754 | ATP biosynthetic process | 4.21E-04 | 8.63E-03 |
| GO:0046034 | ATP metabolic process | 4.21E-04 | 8.63E-03 |
| GO:0015985 | energy coupled proton transport, down electrochemical gradient | 4.21E-04 | 8.63E-03 |
| GO:0015986 | ATP synthesis coupled proton transport | 4.21E-04 | 8.63E-03 |
| GO:0009141 | nucleoside triphosphate metabolic process | 4.21E-04 | 8.63E-03 |
| GO:0009142 | nucleoside triphosphate biosynthetic process | 4.21E-04 | 8.63E-03 |
| GO:0009144 | purine nucleoside triphosphate metabolic process | 4.21E-04 | 8.63E-03 |
| GO:0009145 | purine nucleoside triphosphate biosynthetic process | 4.21E-04 | 8.63E-03 |
| GO:0009206 | purine ribonucleoside triphosphate biosynthetic process | 4.21E-04 | 8.63E-03 |
| GO:0009205 | purine ribonucleoside triphosphate metabolic process | 4.21E-04 | 8.63E-03 |
| GO:0009201 | ribonucleoside triphosphate biosynthetic process | 4.21E-04 | 8.63E-03 |

**Table S48: GO enrichment of highly expressed genes in ephyra compared to medusa stage.**

| GO Id | GO Term | *P*-value | FDR |
| --- | --- | --- | --- |
| GO:0003735 | structural constituent of ribosome | 5.64E-28 | 4.93E-25 |
| GO:1901566 | organonitrogen compound biosynthetic process | 2.76E-26 | 1.21E-23 |
| GO:0043604 | amide biosynthetic process | 2.77E-25 | 8.07E-23 |
| GO:0006412 | translation | 4.69E-25 | 8.20E-23 |
| GO:0043043 | peptide biosynthetic process | 4.69E-25 | 8.20E-23 |
| GO:0005840 | ribosome | 1.08E-24 | 1.57E-22 |
| GO:0005198 | structural molecule activity | 4.89E-23 | 6.11E-21 |
| GO:0006518 | peptide metabolic process | 5.75E-23 | 6.28E-21 |
| GO:0043603 | cellular amide metabolic process | 9.30E-23 | 9.03E-21 |
| GO:0034645 | cellular macromolecule biosynthetic process | 1.71E-22 | 1.49E-20 |
| GO:1901576 | organic substance biosynthetic process | 1.02E-20 | 8.11E-19 |
| GO:0044271 | cellular nitrogen compound biosynthetic process | 2.66E-20 | 1.94E-18 |
| GO:0044249 | cellular biosynthetic process | 7.15E-20 | 4.72E-18 |
| GO:0030529 | intracellular ribonucleoprotein complex | 8.10E-20 | 4.72E-18 |
| GO:1990904 | ribonucleoprotein complex | 8.10E-20 | 4.72E-18 |
| GO:0009059 | macromolecule biosynthetic process | 1.51E-19 | 8.25E-18 |
| GO:0009058 | biosynthetic process | 4.63E-19 | 2.38E-17 |
| GO:0044444 | cytoplasmic part | 1.16E-17 | 5.63E-16 |
| GO:0043232 | intracellular non-membrane-bounded organelle | 1.99E-15 | 8.70E-14 |
| GO:0043228 | non-membrane-bounded organelle | 1.99E-15 | 8.70E-14 |
| GO:0005622 | intracellular | 4.53E-11 | 1.89E-09 |
| GO:0034641 | cellular nitrogen compound metabolic process | 3.25E-08 | 1.29E-06 |
| GO:0032991 | macromolecular complex | 5.18E-08 | 1.97E-06 |
| GO:0044424 | intracellular part | 1.84E-06 | 6.70E-05 |
| GO:1901564 | organonitrogen compound metabolic process | 2.21E-06 | 7.73E-05 |
| GO:0044391 | ribosomal subunit | 3.58E-06 | 1.20E-04 |
| GO:0043226 | organelle | 3.87E-06 | 1.25E-04 |
| GO:0043229 | intracellular organelle | 7.58E-06 | 2.37E-04 |
| GO:0044267 | cellular protein metabolic process | 2.83E-05 | 8.53E-04 |
| GO:0044464 | cell part | 4.30E-05 | 1.25E-03 |
| GO:0005852 | eukaryotic translation initiation factor 3 complex | 4.17E-04 | 1.18E-02 |
| GO:0019538 | protein metabolic process | 5.19E-04 | 1.42E-02 |
| GO:0015934 | large ribosomal subunit | 5.48E-04 | 1.45E-02 |
| GO:0031072 | heat shock protein binding | 7.68E-04 | 1.97E-02 |
| GO:0008152 | metabolic process | 8.36E-04 | 2.08E-02 |
| GO:1901137 | carbohydrate derivative biosynthetic process | 8.56E-04 | 2.08E-02 |
| GO:0006807 | nitrogen compound metabolic process | 9.18E-04 | 2.17E-02 |

**Table S49: GO enrichment of highly expressed genes in medusa compared to ephyra stage.**

| GO Id | GO Term | *P*-value | FDR |
| --- | --- | --- | --- |
| GO:0005576 | extracellular region | 5.73E-07 | 6.91E-04 |
| GO:0030246 | carbohydrate binding | 1.40E-05 | 8.44E-03 |
| GO:0000145 | exocyst | 7.85E-05 | 2.37E-02 |
| GO:0044448 | cell cortex part | 7.85E-05 | 2.37E-02 |
| GO:0044421 | extracellular region part | 1.19E-04 | 2.77E-02 |
| GO:0002376 | immune system process | 1.47E-04 | 2.77E-02 |
| GO:0005509 | calcium ion binding | 2.18E-04 | 2.77E-02 |
| GO:0006952 | defense response | 2.30E-04 | 2.77E-02 |
| GO:0008237 | metallopeptidase activity | 2.42E-04 | 2.77E-02 |
| GO:0031012 | extracellular matrix | 2.51E-04 | 2.77E-02 |
| GO:0005201 | extracellular matrix structural constituent | 2.53E-04 | 2.77E-02 |
| GO:0007160 | cell-matrix adhesion | 4.91E-04 | 4.55E-02 |
| GO:0031589 | cell-substrate adhesion | 4.91E-04 | 4.55E-02 |

**7.3 Retinoic acid signaling in cnidarians**

Retinoic acid (RA) is a metabolite of vitamin A (retinol) that influences a range of essential biological processes such as growth and development [102]. RA acts by binding to the retinoic acid receptor (RAR), which is bound to DNA as a heterodimer with the retinoid X receptor (RXR). The RXR is a type of nuclear receptor that is activated by 9-cis retinoic acid. In a previous study, the RXR was found to belong to the core molecular module that regulates metamorphosis from polyp to medusa in the jellyfish [31]. Despite its biological importance, only one *RXR* was previously identified in Cnidaria while *RAR* was reported to be absent [103]. Interestingly, we found the *RXR* gene in *Nemopilema* using the BLAST reciprocal best hit method, although it is absent in other non-medusa-forming cnidarians (*Hydra*, *Acropora*, and *Nematostella*). Furthermore, the *RXR* genes in other medusa-forming cnidarians, including *Tripedalia cystophora* (Cubozoa), *Clytia hemisphaerica* (Hydrozoa), and *Aurelia aurita* (Scyphozoa), show remarkable homology to the vertebrate *RXR* [31, 104], suggesting retinoid signaling was an important regulatory mechanism already in early metazoans. Multiple sequence alignment and phylogenetic analysis of *RXR* genes show that the *Nemopilema* *RXR* gene forms a monophyletic clade with *Aurelia* and *Tripedalia* (Figure S24). Given this sequence similarity and presence only in cnidarian species that have a medusa life stage, it is possible that the *RXR* gene plays a prominent role in the metamorphosis from polyp to medusa.

**
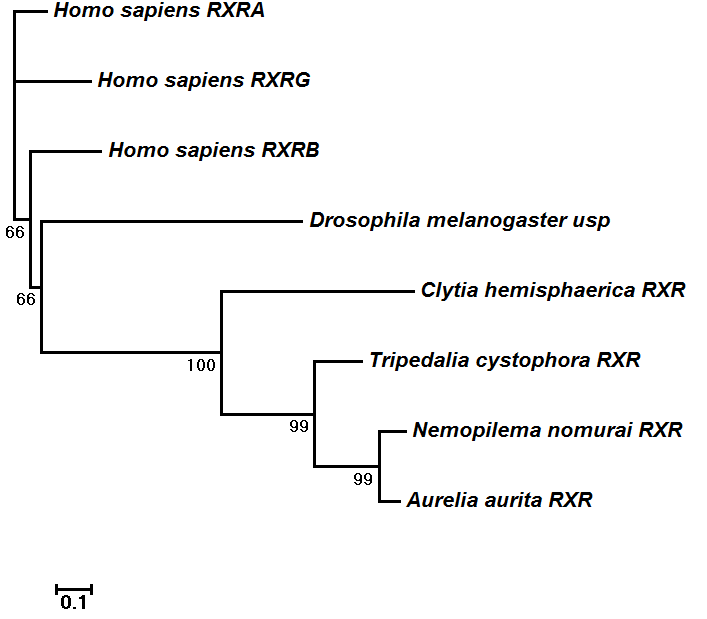
**

**Figure S24: Unrooted tree of *Nemopilema* *RXR* gene with other medusa forming cnidarians.** The scale bar indicates the branch lengths measured in the number of amino acid substitutions per site.

**7.4 Retinoic acid response elements and nearby genes**

Retinoic acid response elements (RAREs) are DNA regions that bind the RAR-RXR protein complex [33]. RXR enhances the binding of the RAR protein to RAREs sequences [105] and binding of the retinoic acid ligand to RAR alters the conformation of the RAR, which in turn affects the binding of other proteins that either induce or repress transcription of a nearby target genes, including Hox [106]. The RAREs are composed of two direct repeats of a core hexameric DNA motif “(A/G)G(G/T)TCA” with 5 bp spaced between the two repeats [107]. We searched this hexameric DNA motif in *Nemopilema* as well as all other species in this study. While the RAR gene was absent in *Nemopilema* and all other members of Cnidaria, we did find 1,630 RAREs regions in *Nemopilema* with an average distance of 13 Kbp to the nearest gene (Table S50). Furthermore, the identified RAREs regions were localized with high abundance of transcription start sites (TSS) in the four cnidarians, indicating that cnidarians may use RAREs to regulate nearby genes (Figure S25). Numerous genes within the retinoid pathway and Hox gene family were found to harbor RAREs [106]. To further identify functional and/or evolutionarily conserved genes that have nearby RAREs, we used the descriptions from Rhinn *et al*. (2012) were used. The presence or absence of a gene was determined by using the BLAST reciprocal best hit method. *Nemopilema* and *Aurelia* possessed the largest number of genes within ±20 Kbp from RAREs among non-bilaterian metazoans (Tables S51 and S52).


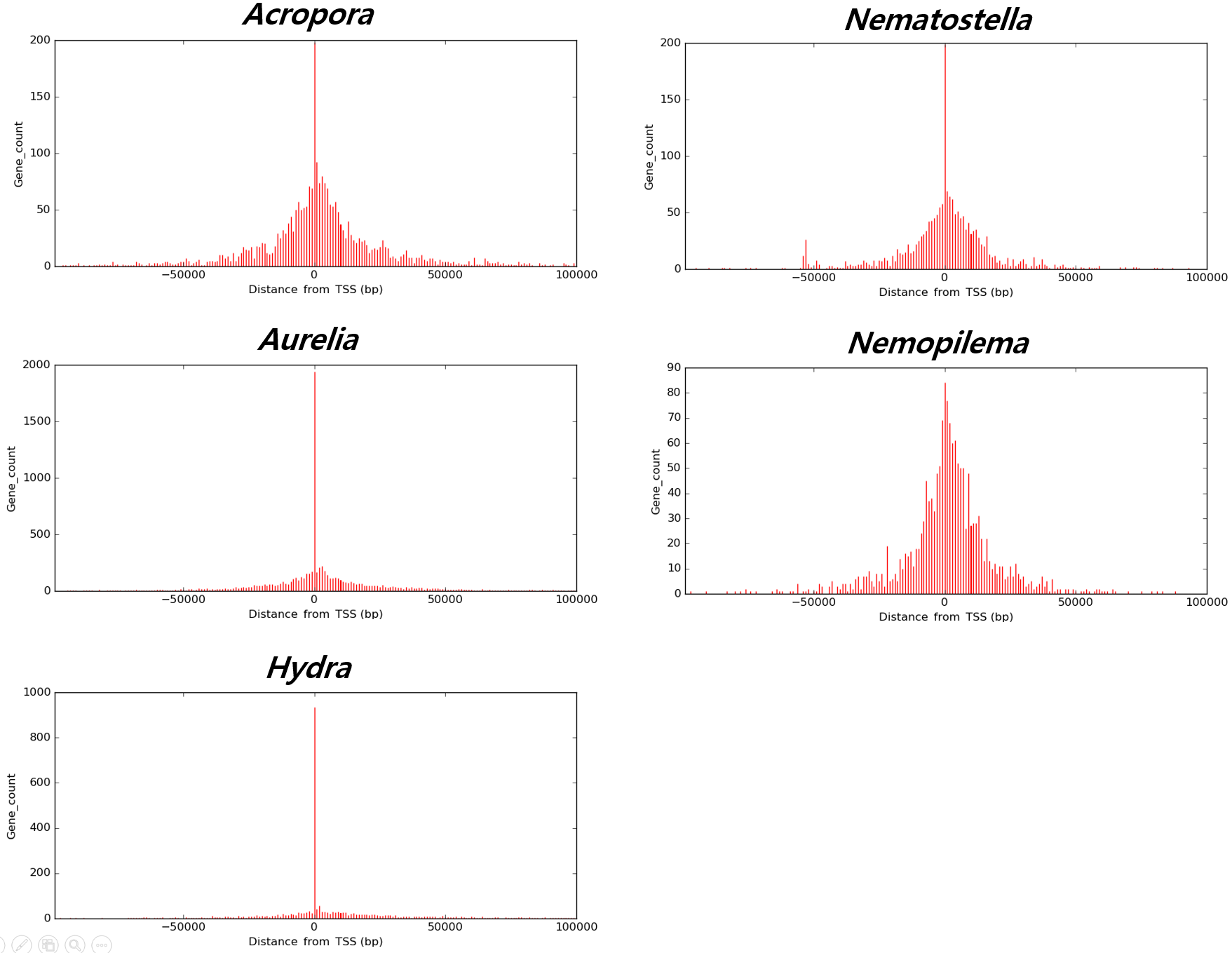


**Figure S25: Genome-wide distribution of the identified RAREs in Cnidarians.**

X-axis denote regions between -100Kbp and +100Kbp from TSS, Y-axis denote number of genes in RAREs.

**Table S50: RAREs in metazoan and holozoan genomes.** TSS = transcription start site.

| Species | Genome size | # of filtered genes | # of RAREs regions | # of genes (TSS) within ±20 Kbp from RAREs | Avg. distance to nearest gene (bp) | Normalized avg. distance to nearest gene |
| --- | --- | --- | --- | --- | --- | --- |
| *A. digitifera* | 447,497,157 | 25,295 | 2,606 | 5,242 | 20,423 | 1.15 |
| *A. queenslandica* | 166,699,561 | 12,811 | 819 | 2,112 | 18,048 | 1.39 |
| *A. aurita* | 757,170,055 | 25,174 | 8,246 | 9,821 | 18,187 | 0.60 |
| *C. elegans* | 100,286,401 | 20,256 | 500 | 3,661 | 4,976 | 1.01 |
| *D. rerio* | 1,371,719,383 | 25,654 | 7,836 | 5,016 | 83,360 | 1.56 |
| *D. melanogaster* | 143,726,002 | 13,864 | 666 | 2,408 | 34,103 | 3.29 |
| *H. sapiens* | 3,209,286,105 | 19,797 | 20,098 | 5,101 | 297,133 | 1.83 |
| *H. vulgaris* | 852,170,992 | 17,331 | 2,663 | 1,862 | 21,010 | 0.43 |
| *M. leidyi* | 155,865,547 | 15,922 | 1,509 | 4,457 | 10,776 | 1.10 |
| *M. brevicollis* | 41,709,928 | 9,153 | 436 | 3,279 | 3,992 | 0.88 |
| *N. nomurai* | 213,630,333 | 18,962 | 1,630 | 4,751 | 13,170 | 1.17 |
| *N. vectensis* | 356,613,585 | 24,567 | 1,803 | 4,021 | 13,073 | 0.90 |
| *T. adhaerens* | 105,631,681 | 11,491 | 290 | 1,211 | 8,324 | 0.91 |

**Table S51: Genes within ±20 Kbp from RAREs in non-bilaterian metazoans.** Gene symbols in table are based on human gene.

| Genes from Rhinn *et al*. | *A. digitifera* | *A. queenslandica* | *A.aurita* | *H. vulgaris* | *M. leidyi* | *M. brevicollis* | *N. nomurai* | *N. vectensis* | *T. adhaerens* |
| --- | --- | --- | --- | --- | --- | --- | --- | --- | --- |
| *ADH7* | *-* | *-* | *-* | *-* | *-* | *ADH5* | *-* | *-* | *-* |
| *ADRB1* | *-* | *-* | *-* | *-* | *-* | *-* | *ADRB2* | *-* | *-* |
| *AFP* | *-* | *-* | *-* | *-* | *-* | *-* | *-* | *-* | *-* |
| *ALDH1A1* | *-* | *-* | *-* | *-* | *-* | *ALDH1A2* | *-* | *-* | *-* |
| *APOA1* | *-* | *-* | *-* | *-* | *-* | *-* | *-* | *-* | *-* |
| *APOA2* | *-* | *-* | *-* | *-* | *-* | *-* | *-* | *-* | *-* |
| *APOC3* | *-* | *-* | *-* | *-* | *-* | *-* | *-* | *-* | *-* |
| *CD38* | *CD207* | *-* | *CD207* | *-* | *-* | *-* | *-* | *CD109* | *CD109* |
| *CDX1* | *-* | *-* | *-* | *-* | *-* | *-* | *-* | *-* | *-* |
| *CEBPE* | *-* | *-* | *-* | *-* | *-* | *-* | *-* | *-* | *-* |
| *CRABP2* | *-* | *-* | *-* | *-* | *-* | *-* | *-* | *-* | *-* |
| *CRYAB* | *-* | *-* | *-* | *CRYAB* | *-* | *-* | *-* | *-* | *-* |
| *CSH1* | *-* | *-* | *-* | *-* | *-* | *-* | *-* | *-* | *-* |
| *CYP24A1* | *CYP24A1* | *-* | *CYP24A1* | *-* | *-* | *-* | *CYP24A1* | *-* | *-* |
| *CYP26A1* | *-* | *-* | *-* | *-* | *-* | *-* | *-* | *-* | *-* |
| *DRD2* | *-* | *-* | *-* | *-* | *-* | *-* | *DRD2* | *DRD1* | *-* |
| *EGR1* | *-* | *-* | *-* | *-* | *-* | *-* | *-* | *-* | *-* |
| *EPO* | *-* | *-* | *-* | *-* | *-* | *-* | *-* | *-* | *-* |
| *ETS1* | *-* | *-* | *-* | *-* | *-* | *-* | *-* | *-* | *-* |
| *FGF8* | *FGF2,*  *FGF18* | *-* | *FGF18* | *-* | *-* | *-* | *FGF16* | *-* | *-* |
| *FOXA1* | *-* | *-* | *FOXA1* | *-* | *-* | *-* | *FOXA1* | *-* | *-* |
| *GH1* | *-* | *-* | *-* | *-* | *-* | *-* | *-* | *-* | *-* |
| *GH2* | *-* | *-* | *-* | *-* | *-* | *-* | *-* | *-* | *-* |
| *GNRH1* | *-* | *-* | *-* | *-* | *-* | *-* | *-* | *-* | *-* |
| *HNF1A* | *-* | *-* | *-* | *-* | *-* | *-* | *-* | *-* | *-* |
| *HOXA1* | *-* | *-* | *HOXA6* | *-* | *-* | *-* | *HOXA10* | *-* | *-* |
| *HOXA4* | *-* | *-* | *HOXA6* | *-* | *-* | *-* | *HOXA10* | *-* | *-* |
| *HOXB1* | *-* | *-* | *HOXB7* | *-* | *-* | *-* | *-* | *-* | *-* |
| *HOXB4* | *-* | *-* | *HOXB7* | *-* | *-* | *-* | *-* | *-* | *-* |
| *HOXB5* | *-* | *-* | *HOXB7* | *-* | *-* | *-* | *-* | *-* | *-* |
| *HOXD4* | *-* | *-* | *-* | *-* | *-* | *-* | *-* | *-* | *-* |
| *HSD17B1* | *HSD17B4,*  *HSD17B14* | *HSD17B7* | *HSD17B10* | *-* | *HSD17B10* | *HSD17B10,*  *HSD17B4* | *-* | *HSD17B4* | *-* |
| *ICAM1* | *-* | *-* | *-* | *-* | *-* | *-* | *-* | *-* | *-* |
| *IL2RA* | *-* | *-* | *-* | *-* | *-* | *-* | *-* | *-* | *-* |
| *ITGB3* | *-* | *-* | *-* | *-* | *-* | *-* | *-* | *-* | *-* |
| *LAMB1* | *-* | *-* | *LAMB1* | *LAMB1* | *-* | *-* | *-* | *LAMB1* | *-* |
| *LEFTY1* | *-* | *-* | *-* | *-* | *-* | *-* | *-* | *-* | *-* |
| *MDK* | *-* | *-* | *-* | *-* | *-* | *-* | *-* | *-* | *-* |
| *MMP11* | *-* | *-* | *MMP1* | *-* | *-* | *-* | *MMP1* | *MMP25,*  *MMP14* | *-* |
| *NES* | *-* | *-* | *-* | *-* | *-* | *-* | *-* | *-* | *-* |
| *NEUROG2* | *NEUROG2* | *-* | *-* | *-* | *-* | *-* | *-* | *NEUROG1* | *-* |
| *NGFR* | *-* | *-* | *-* | *-* | *-* | *-* | *-* | *-* | *-* |
| *NODAL* | *-* | *-* | *-* | *-* | *-* | *-* | *-* | *-* | *-* |
| *NR2C1* | *-* | *-* | *-* | *-* | *-* | *-* | *-* | *-* | *-* |
| *OLIG2* | *-* | *-* | *-* | *-* | *-* | *-* | *-* | *-* | *-* |
| *OXT* | *-* | *-* | *-* | *-* | *-* | *-* | *-* | *-* | *-* |
| *PAX6* | *-* | *-* | *PAX5* | *-* | *PAX2* | *-* | *PAX2* | *PAX6* | *-* |
| *PCK1* | *-* | *-* | *-* | *-* | *-* | *-* | *-* | *-* | *-* |
| *PCP2* | *-* | *-* | *-* | *-* | *-* | *-* | *-* | *-* | *-* |
| *PDX1* | *-* | *-* | *-* | *-* | *-* | *-* | *-* | *-* | *-* |
| *PITX2* | *-* | *-* | *-* | *-* | *-* | *-* | *-* | *-* | *-* |
| *PLAT* | *-* | *-* | *-* | *-* | *-* | *-* | *-* | *-* | *-* |
| *POU1F1* | *-* | *POU1F1* | *-* | *-* | *POU1F1* | *-* | *POU1F1* | *-* | *-* |
| *POU5F1* | *-* | *-* | *-* | *-* | *-* | *-* | *-* | *-* | *-* |
| *PRKCA* | *-* | *-* | *PRKCA* | *-* | *-* | *-* | *PRKCA* | *-* | *-* |
| *PTAFR* | *-* | *-* | *-* | *-* | *-* | *-* | *-* | *-* | *-* |
| *PTH1R* | *-* | *-* | *-* | *-* | *-* | *-* | *-* | *-* | *-* |
| *RARA* | *-* | *-* | *-* | *-* | *-* | *-* | *-* | *-* | *-* |
| *RARB* | *-* | *-* | *-* | *-* | *-* | *-* | *-* | *-* | *-* |
| *RARG* | *-* | *-* | *-* | *-* | *-* | *-* | *-* | *-* | *-* |
| *RBP1* | *-* | *-* | *-* | *-* | *-* | *-* | *-* | *-* | *-* |
| *SFTPB* | *-* | *-* | *-* | *-* | *-* | *-* | *-* | *-* | *-* |
| *SHH* | *-* | *-* | *-* | *-* | *-* | *-* | *-* | *-* | *-* |
| *STAT1* | *-* | *-* | *-* | *-* | *-* | *-* | *-* | *-* | *-* |
| *TGM2* | *-* | *TGM1* | *-* | *-* | *-* | *-* | *-* | *-* | *-* |
| *TLX2* | *-* | *-* | *-* | *-* | *-* | *-* | *-* | *-* | *-* |
| *TSHB* | *-* | *-* | *-* | *-* | *-* | *-* | *-* | *-* | *-* |
| *UCP1* | *-* | *-* | *-* | *-* | *UCP2* | *UCP2* | *-* | *-* | *-* |

**Table S52: Hox genes that are located nearby RAREs in *Aurelia*.**

| Scaffold | RARE  start | RARE  end | RARE  sequence | Gene ID | Distance to RAREs | HOX gene  type |
| --- | --- | --- | --- | --- | --- | --- |
| Seg1063 | 125,353 | 125,369 | TGAACTTGAATTGAACT | Seg1063.3 | 8,098 | *HOXA* |
| Seg2155 | 97,263 | 97,279 | AGGTGACGGAGAGGTCA | Seg2155.5 | -4,188 | *HOXEc* |

**8 Venom proteins in cnidarians**

Cnidarians are known to produce complex mixtures of proteinaceous venoms used for prey capture and defense [108]. Although Scyphozoa are known to be less dangerous to humans than jellyfish of the class Cubozoa, *Nemopilema* can lead to systemic symptoms and have caused human fatalities [19]. Phospholipase A_2_ (PLA_2_) and metalloproteases are well-known toxin-related enzymes found in Scyphozoa [39]. The PLA_2_ enzyme hydrolyzes the sn-2 acyl bond of glycerophospholipids to produce fatty acids including arachidonic acid and lysophospholipid [109, 110]. Toxic functions of PLA_2_ in cnidarian venoms have been proposed to include defense and immobilization and digestion of prey [111].

To understand the evolution of venom in jellyfish, we scanned non-bilaterian metazoan gene sets for all toxin related domains in the Tox-Prot database [35]. A total of 6,523 proteins containing 136 unique domains were included in this analysis. We conducted a multiple sequence alignment using MUSCLE for each venom related domain, which was then used to generate profile Hidden Markov Models (profile HMMs). A total of 136 domain profile HMMs were generated and aligned into eight non-bilaterian metazoan gene sets using the hmmsearch command of HMMER [112]. Domains with *P*-values <0.05 were considered significant. When two or more domains aligned to the same position, the domain with the highest score was chosen. In total, 52 out of 136 toxin domains were found in *Nemopilema* (Table S53).

**Table S53: Venom related domains in non-bilaterian metazoans.**

| Pfam ID | Pfam description | *A. digitifera* | *A. queenslandica* | *A. aurita* | *C. hemisphaerica* | *H. vulgaris* | *M. leidyi* | *N. nomurai* | *N. vectensis* | *T. adhaerens* |
| --- | --- | --- | --- | --- | --- | --- | --- | --- | --- | --- |
| PF12796 | Ankyrin repeats (3 copies) | 276 | 398 | 187 | 209 | 151 | 129 | 173 | 161 | 84 |
| PF00008 | EGF-like domain | 191 | 41 | 98 | 79 | 36 | 14 | 85 | 232 | 84 |
| PF13499 | EF-hand domain pair | 76 | 36 | 56 | 72 | 42 | 81 | 77 | 83 | 36 |
| PF01400 | Astacin (Peptidase family M12A) | 32 | 20 | 68 | 86 | 53 | 13 | 64 | 84 | 6 |
| PF13637 | Ankyrin repeats (many copies) | 101 | 125 | 57 | 47 | 57 | 27 | 58 | 33 | 19 |
| PF00147 | Fibrinogen beta and gamma chains, C-terminal globular domain | 80 | 78 | 99 | 125 | 16 | 0 | 52 | 87 | 1 |
| PF00089 | Trypsin | 75 | 3 | 65 | 89 | 23 | 17 | 51 | 85 | 22 |
| PF00754 | F5/8 type C domain | 214 | 0 | 46 | 26 | 33 | 0 | 46 | 305 | 1 |
| PF01391 | Collagen triple helix repeat (20 copies) | 111 | 26 | 54 | 31 | 15 | 19 | 30 | 44 | 2 |
| PF01421 | Reprolysin (M12B) family zinc metalloprotease | 20 | 7 | 21 | 20 | 16 | 10 | 23 | 18 | 19 |
| PF07648 | Kazal-type serine protease inhibitor domain | 19 | 1 | 14 | 24 | 9 | 9 | 20 | 20 | 2 |
| PF00059 | Lectin C-type domain | 113 | 0 | 12 | 65 | 38 | 17 | 16 | 44 | 30 |
| PF00188 | Cysteine-rich secretory protein family | 38 | 2 | 27 | 43 | 35 | 17 | 15 | 60 | 4 |
| PF00050 | Kazal-type serine protease inhibitor domain | 19 | 1 | 14 | 15 | 13 | 10 | 14 | 18 | 6 |
| PF01562 | Reprolysin family propeptide | 16 | 0 | 4 | 10 | 11 | 0 | 13 | 8 | 5 |
| PF05826 | Phospholipase A2 | 2 | 0 | 4 | 3 | 4 | 0 | 11 | 3 | 0 |
| PF00135 | Carboxylesterase family | 6 | 1 | 8 | 4 | 1 | 18 | 8 | 24 | 24 |
| PF01593 | Flavin containing amine oxidoreductase | 14 | 8 | 6 | 4 | 3 | 3 | 8 | 13 | 5 |
| PF13833 | EF-hand domain pair | 6 | 1 | 8 | 4 | 3 | 8 | 8 | 8 | 2 |
| PF00200 | Disintegrin | 5 | 1 | 12 | 10 | 3 | 1 | 8 | 2 | 8 |
| PF06607 | Prokineticin | 3 | 0 | 5 | 7 | 5 | 0 | 8 | 1 | 0 |
| PF01549 | ShK domain-like | 1 | 0 | 11 | 3 | 3 | 4 | 8 | 0 | 0 |
| PF01663 | Type I phosphodiesterase / nucleotide pyrophosphatase | 7 | 2 | 10 | 12 | 6 | 1 | 7 | 10 | 6 |
| PF14670 | Coagulation Factor Xa inhibitory site | 4 | 16 | 5 | 8 | 5 | 4 | 7 | 6 | 5 |
| PF00151 | Lipase | 13 | 0 | 6 | 4 | 4 | 0 | 6 | 7 | 5 |
| PF00014 | Kunitz/Bovine pancreatic trypsin inhibitor domain | 11 | 6 | 9 | 7 | 6 | 0 | 5 | 24 | 0 |
| PF00095 | WAP-type (Whey Acidic Protein) 'four-disulfide core' | 9 | 1 | 8 | 8 | 2 | 1 | 5 | 11 | 1 |
| PF08516 | ADAM cysteine-rich | 4 | 1 | 6 | 6 | 7 | 2 | 5 | 3 | 7 |
| PF00023 | Ankyrin repeat | 43 | 26 | 5 | 11 | 7 | 8 | 5 | 2 | 3 |
| PF00219 | Insulin-like growth factor binding protein | 3 | 1 | 5 | 5 | 15 | 0 | 4 | 1 | 2 |
| PF00149 | Calcineurin-like phosphoesterase | 2 | 2 | 7 | 2 | 2 | 3 | 4 | 1 | 3 |
| PF02872 | 5'-nucleotidase, C-terminal domain | 1 | 1 | 8 | 3 | 2 | 2 | 4 | 1 | 3 |
| PF07732 | Multicopper oxidase | 5 | 0 | 4 | 0 | 0 | 0 | 4 | 0 | 0 |
| PF00326 | Prolyl oligopeptidase family | 5 | 2 | 1 | 3 | 2 | 3 | 3 | 4 | 5 |
| PF07678 | A-macroglobulin complement component | 5 | 0 | 4 | 2 | 2 | 2 | 3 | 4 | 2 |
| PF10569 | Alpha-macro-globulin thiol-ester bond-forming region | 4 | 0 | 2 | 2 | 2 | 1 | 3 | 4 | 2 |
| PF00207 | Alpha-2-macroglobulin family | 4 | 0 | 4 | 2 | 2 | 2 | 3 | 3 | 2 |
| PF07703 | Alpha-2-macroglobulin family N-terminal region | 4 | 0 | 3 | 1 | 1 | 0 | 3 | 1 | 2 |
| PF00031 | Cystatin domain | 0 | 0 | 2 | 2 | 4 | 2 | 3 | 1 | 0 |
| PF04916 | Phospholipase B | 3 | 1 | 0 | 1 | 2 | 3 | 2 | 4 | 1 |
| PF01223 | DNA/RNA non-specific endonuclease | 1 | 0 | 1 | 2 | 1 | 0 | 2 | 3 | 5 |
| PF07677 | A-macroglobulin receptor | 5 | 0 | 3 | 1 | 2 | 0 | 2 | 1 | 1 |
| PF00068 | Phospholipase A2 | 8 | 0 | 0 | 0 | 2 | 0 | 1 | 10 | 5 |
| PF01823 | MAC/Perforin domain | 14 | 0 | 4 | 9 | 6 | 4 | 1 | 9 | 0 |
| PF01826 | Trypsin Inhibitor like cysteine rich domain | 1 | 6 | 1 | 3 | 4 | 6 | 1 | 4 | 1 |
| PF01630 | Hyaluronidase | 2 | 0 | 2 | 0 | 1 | 0 | 1 | 4 | 0 |
| PF00930 | Dipeptidyl peptidase IV (DPP IV) N-terminal region | 2 | 1 | 0 | 2 | 2 | 2 | 1 | 2 | 4 |
| PF00838 | Translationally controlled tumour protein | 2 | 1 | 1 | 1 | 2 | 1 | 1 | 1 | 3 |
| PF04389 | Peptidase family M28 | 1 | 1 | 2 | 1 | 1 | 1 | 1 | 1 | 1 |
| PF01835 | MG2 domain | 0 | 0 | 0 | 2 | 1 | 2 | 1 | 1 | 0 |
| PF00341 | PDGF/VEGF domain | 1 | 0 | 1 | 4 | 1 | 0 | 1 | 0 | 0 |
| PF10279 | Latarcin precursor | 0 | 0 | 0 | 0 | 0 | 0 | 1 | 0 | 0 |
| PF00706 | Anenome neurotoxin | 0 | 0 | 0 | 0 | 0 | 0 | 0 | 9 | 0 |
| PF06369 | Sea anemone cytotoxic protein | 5 | 0 | 1 | 3 | 4 | 0 | 0 | 1 | 0 |
| PF02950 | Conotoxin | 0 | 0 | 1 | 2 | 0 | 0 | 0 | 1 | 0 |
| PF08086 | Ergtoxin family | 0 | 0 | 0 | 0 | 0 | 0 | 0 | 1 | 0 |
| PF03318 | Clostridium epsilon toxin ETX/Bacillus mosquitocidal toxin MTX2 | 0 | 0 | 0 | 6 | 2 | 0 | 0 | 0 | 0 |
| PF00622 | SPRY domain | 1 | 0 | 1 | 1 | 2 | 0 | 0 | 0 | 1 |
| PF13653 | Glycerophosphoryl diester phosphodiesterase family | 1 | 0 | 0 | 1 | 1 | 0 | 0 | 0 | 1 |
| PF00087 | Snake toxin | 0 | 0 | 0 | 0 | 0 | 1 | 0 | 0 | 0 |
| PF00184 | Neurohypophysial hormones, C-terminal Domain | 1 | 0 | 0 | 0 | 0 | 0 | 0 | 0 | 0 |
| PF02819 | Spider toxin | 1 | 0 | 0 | 0 | 0 | 0 | 0 | 0 | 0 |
| PF03032 | Brevenin/esculentin/gaegurin/rugosin family | 0 | 0 | 0 | 1 | 0 | 0 | 0 | 0 | 0 |
| PF08116 | PhTx neurotoxin family | 0 | 0 | 0 | 0 | 0 | 1 | 0 | 0 | 0 |
| PF08562 | Crisp | 0 | 0 | 0 | 0 | 0 | 1 | 0 | 0 | 0 |
| PF13574 | Metallo-peptidase family M12B Reprolysin-like | 0 | 0 | 0 | 1 | 0 | 0 | 0 | 0 | 0 |
| PF15658 | Latrotoxin C-terminal domain | 1 | 0 | 0 | 0 | 0 | 0 | 0 | 0 | 0 |
